# An anionic human protein mediates cationic liposome delivery of genome editing proteins into mammalian cells

**DOI:** 10.1038/s41467-019-10828-3

**Published:** 2019-07-02

**Authors:** Y. Bill Kim, Kevin T. Zhao, David B. Thompson, David R. Liu

**Affiliations:** 1 Merkin Institute of Transformative Technologies in Healthcare, Broad Institute of Harvard and MIT, Cambridge, MA 02142 USA; 2 Department of Chemistry and Chemical Biology, Harvard University, Cambridge, MA 02138 USA; 3 Howard Hughes Medical Institute, Harvard University, Cambridge, MA 02138 USA; 4 Department of Genetics, Harvard Medical School, Boston, MA 02115 USA; 5 Wyss Institute for Biologically Inspired Engineering, Boston, MA 02115 USA

**Keywords:** Genetic engineering, Biotechnology, Protein delivery

## Abstract

Delivery into mammalian cells remains a significant challenge for many applications of proteins as research tools and therapeutics. We recently reported that the fusion of cargo proteins to a supernegatively charged (–30)GFP enhances encapsulation by cationic lipids and delivery into mammalian cells. To discover polyanionic proteins with optimal delivery properties, we evaluate negatively charged natural human proteins for their ability to deliver proteins into cultured mammalian cells and human primary fibroblasts. Here we discover that ProTα, a small, widely expressed, intrinsically disordered human protein, enables up to ~10-fold more efficient cationic lipid-mediated protein delivery compared to (–30)GFP. ProTα enables efficient delivery at low- to mid-nM concentrations of two unrelated genome editing proteins, Cre recombinase and zinc-finger nucleases, under conditions in which (–30)GFP fusion or cationic lipid alone does not result in substantial activity. ProTα may enable mammalian cell protein delivery applications when delivery potency is limiting.

## Abstract (web summary)

Inefficient delivery of proteins into mammalian cells limits their use in research and therapy. Here, the authors discover that ProTα, a small, intrinsically disordered human protein can facilitate efficient cationic lipid-mediated protein delivery of genome editing proteins into mammalian cells.

## Introduction

Proteins including genome editing agents represent an increasing proportion of biomedical research tools and new human therapeutics^1^. Due to their inability to spontaneously cross the lipid bilayer, however, current uses of protein tools and therapeutic agents mostly target extracellular components^2^. Technologies to facilitate cytosolic access by proteins are critical to expanding the potential targets that can be accessed efficiently by exogenous proteins.

Researchers have developed many approaches to facilitate protein translocation into the cytosol. Positively charged cell-penetrating peptides, such as the HIV-1 transactivator of transcription (Tat) peptide^3,4^, poly-arginine^5^, or superpositively charged proteins^2,6,7^, allow a fused cargo protein to reach the cytosol via interaction with the highly anionic proteoglycans on the cell surface, followed by endocytosis^8^. However, escape from endosomes is generally inefficient, and unencapsulated proteins are susceptible to proteolytic degradation and neutralization by serum proteins and the extracellular matrix^9^.

Lipid nanoparticles can encapsulate proteins to prevent degradation and neutralization by antibodies^10^. While cationic lipids are routinely used to transfect polyanionic nucleic acids into mammalian cells, they have not been widely used for protein delivery, as proteins vary widely in their ability to be encapsulated by cationic lipids^11^. To impart nucleic-acid-like properties onto proteins, we previously demonstrated that fusing an anionic protein, such as an engineered (–30)GFP with many surface-exposed negative charges, to a protein of interest enables its efficient encapsulation and delivery into mammalian cells^12^.

While (–30)GFP enables cationic lipids to deliver a variety of fused cargo proteins^12^, its discovery from protein engineering efforts unrelated to delivery^13^ suggests that more potent anionic proteins that mediate lipid-based protein delivery may exist. Moreover, the non-mammalian origin of (–30)GFP will likely result in immunogenicity, potentially compromising the safety or efficacy of using this protein in vivo^14^.

To address these limitations, we sought to discover an improved protein fusion partner for lipid-mediated protein delivery from the human proteome. We hypothesize that the human proteome, which contains many proteins with high theoretical net charge, likely includes some native highly anionic proteins that would mediate efficient lipid-based delivery of fused cargo proteins. Here we identify ProTα as a small (111-residue) supernegatively charged protein that enables potent delivery of fused cargo proteins into mammalian cells.

## Results

### Identification of highly anionic human proteins

We generated a candidate list of human proteins with >0.75 net theoretical negative charges per kDa from the UniProt protein database (Supplementary Fig. 1)^15^. To focus on proteins that are more likely to be easily expressed and purified as fusion partners with a wide range of potential cargo proteins, we narrowed down the list to proteins with published structures, known bacterial expression, and no annotated disulfide bonds. Based on these criteria, we identified 12 candidate proteins for further analysis (Table 1).

**Table 1:** Protein delivery using Lipofectamine RNAiMAX in HeLa-DsRed cell line

Protein fused to Cre	Concentration at which 50% of cells are recombined (nM)
ProTα	1.1 ± 0.18
Polyadenylate-binding protein-interacting protein 2	290 ± 60
Troponin C	57 ± 19
DPH3 homolog	36 ± 5
RNA Polymerase II Subunit F	110 ± 58
Multiple coagulation factor deficiency protein 2	140 ± 170
ADP-ribosylation factor-like protein 2-binding protein	20 ± 9
NF-kappa-B inhibitor alpha	63 ± 7
DNA damage-inducible transcript 3 protein	89 ± 5
Carbonic anhydrase VIII	84 ± 22
Protein S100-B	47 ± 60
Sirtuin-1	49 ± 18
(–30)GFP	11 ± 3.0
No fusion	73 ± 12

12 human proteins were chosen as candidates for lipid nanoparticle delivery. EC_50_ is defined as the concentration at which 50 % of the cells contain red fluorescence

The genes encoding the candidate human proteins were cloned as N-terminal fusions to Cre recombinase, overexpressed in *E. coli*, and purified by affinity chromatography. Each purified fusion protein was encapsulated with Lipofectamine RNAiMAX, a commercially available lipid formulation we previously used to deliver anionic proteins and protein complexes^12^. We used HeLa cells containing a genomically integrated DsRed gene preceded by a floxed transcriptional terminator (HeLa-DsRed)^6^ to screen the fusion proteins for Cre delivery activity. Functional Cre delivery catalyzes recombination to remove the terminator, resulting in DsRed expression and red fluorescence. We defined Cre delivery potency as the protein concentration at which 50% of analyzed cells contain red fluorescence (EC_50_). While unfused Cre with RNAiMAX lipid induced red fluorescence in our assay at an EC_50_ of 73 ± 12 nM, fusion of (−30)GFP to Cre lowered the EC_50_ with RNAiMAX to 11 ± 3.0 nM (Table 1), consistent with our previous findings^12^.

**Fig. 1:**
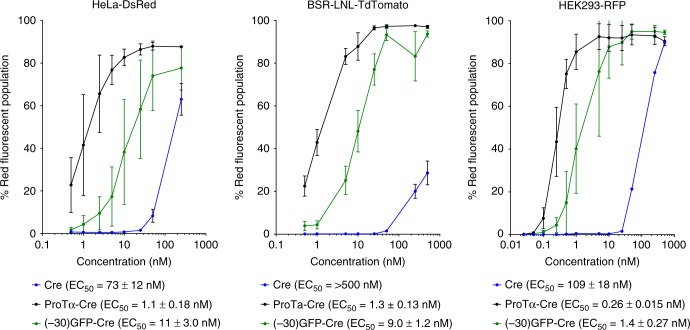
ProTα enables efficient cationic lipid-mediated protein delivery into HeLa-DsRed, BSR-tdTomato, and HEK293-RFP cell lines. EC_50_ is defined as the protein concentration at which 50% of analyzed cells contain red fluorescence. In all cell lines tested, ProTα delivers Cre at low nM concentrations, ~10-fold more potent than (–30)GFP and ~100-fold more efficient than unmodified Cre. Values and error bars represent the mean and standard deviation of three independent biological replicates performed on different days. EC_50_ values were determined by nonlinear fit to the Hill equation, and are followed by the standard errors of the fit. Source data are available in the Source Data file

Seven of the 12 anionic human proteins tested from the set of 12 candidates did not substantially improve the efficacy of protein delivery when fused to the N-terminus of Cre recombinase compared to that of Cre alone (Table 1). Their failure to enhance functional delivery of Cre may be due to an inability to support lipid encapsulation, or due to interference with recombinase activity when fused to Cre. Four human proteins showed compatibility with lipid complexation and enhanced Cre delivery compared to that of unfused Cre, but their delivery potency did not exceed that of (–30)GFP (Table 1). One protein, human prothymosin alpha (ProTα), a small, intrinsically disordered protein^16^ with 111 amino acids and a net theoretical charge of –44, greatly improved the potency of Cre protein delivery into HeLa-DsRed cells 10-fold compared to that of (–30)GFP and 73-fold compared to Cre alone, to an EC_50_ of 1.1 ± 0.18 nM (Table 1 and Fig. 1).

ProTα is universally expressed in human tissues and contains multiple domains that are hypothesized to be modular in function^16^. Researchers have associated multiple biological functions with ProTα, including histone chaperone activity implicated in nucleosome exchange and extracellular immune modulation^16^. A stretch of highly anionic residues in the central part of ProTα was demonstrated to interact with cationic regions of histones^17^.

To verify the highly potent lipid encapsulation and delivery capabilities of ProTα, we delivered ProTα-Cre using lipid nanoparticles into BSR-tdTomato and HEK293-RFP cells, two additional human cell lines containing genomically integrated RFP reporters based on BSR and HEK293^2^ cells. Consistent with the delivery improvement observed in HeLa-DsRed cells, ProTα-Cre also resulted in a large, ~10-fold improvement in delivery potency compared to (–30)GFP-Cre in BSR-tdTomato and HEK293-RFP cells (Fig. 1 and Supplementary Fig. 2). We visually confirmed Cre-mediated fluorescent activation via microscopy to be consistent with these quantified changes in delivery potency (Supplementary Fig. 3). We did not observe any apparent cellular toxicity during ProTα-mediated delivery (Supplementary Fig. 4).

To complement the above Cre recombinase functional delivery experiments, we also quantified the delivery of fluorescent proteins by ProTα fusion. We expressed and purified mCherry, (−30)GFP-mCherry, and ProTα-mCherry, performed cationic lipid-mediated protein delivery into HEK293T cells across a range of protein concentrations, and analyzed the total amount of fluorescent protein in cells by flow cytometry. Compared to the delivery of mCherry alone, delivery of (−30)GFP–mCherry and ProTα–mCherry resulted in more potent delivery of mCherry (Supplementary Fig. 5). To test if ProTα-tagging allows protein delivery into primary cells, many of which suffer from poor liposome-mediated transfection efficiency^18^, we performed cationic lipid-mediated protein delivery using mCherry, (−30)GFP-mCherry, and ProTα-mCherry into human primary fibroblasts. Without any fusion, lipid-mediated mCherry protein delivery exceeded 10% only at concentrations ≥2.5 µM (Supplementary Fig. 6). In contrast, lipid-mediated ProTα-mCherry and (−30)GFP-mCherry delivery was efficient at all concentrations tested (as low as 50 nM; see Supplementary Fig. 6). These results demonstrate that ProTα greatly facilitates cationic lipid-mediated protein delivery even in human primary cells that resist lipofection. In addition, these findings are consistent with a model in which both (−30)GFP and ProTα enable similarly efficient complexation and initial endocytosis into the cell, but ProTα allows proteins such as Cre to more efficiently reach its substrate (in the nucleus) in functional form than (−30)GFP.

### Structure function analysis of ProTα

**Fig. 2:**
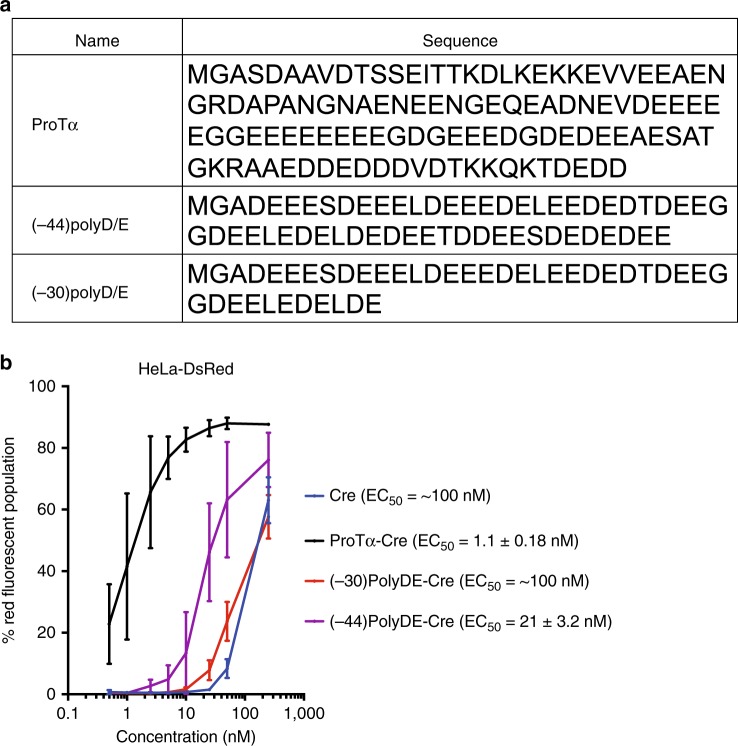
ProTα delivery potency cannot be recapitulated by simple oligomers of acidic residues. **a** Sequences of ProTα and charged peptide analogs that contain –30 or –44 net theoretical charge for comparison. All proteins contain a Gly-Ala inserted after the initial Met to minimize expression level differences. **b** Despite sharing the same net theoretical charge as that of ProTα, (−44)PolyD/E-Cre does not promote potent protein delivery. EC_50_ is defined as the protein concentration at which 50% of analyzed HeLa-DsRed cells contain red fluorescence. Values and error bars represent the mean and standard deviation of three independent biological replicates performed on different days. EC_50_ values were determined by nonlinear fit to the Hill equation, and are followed by the standard errors of the fit. Source data are available in the Source Data file

ProTα contains a stretch of aspartate and glutamate residues that provide a concentrated region of anionic charge^16^. To investigate if a simple stretch of acidic residues of similar negative charge magnitude is sufficient to confer potent delivery with cationic liposome delivery, we generated two protein constructs that have the same theoretical net charge with those of (–30)GFP and ProTα, (–30 and –44, respectively) but with sequences containing only scrambled Glu and Asp residues: (–30)polyD/E and (-44)polyD/E (Fig. 2a). Importantly, Cre fusions to (–30)polyD/E or (−44)polyD/E resulted in much poorer protein delivery potencies (EC_50_ = ~100 nM and 21 ± 3.2 nM, respectively) than fusions with (–30)GFP or ProTα (Fig. 2b). These results show that additional features beyond net theoretical charge within ProTα strongly contribute to its ability to enable highly potent lipid-mediated protein delivery.

**Fig. 3:**
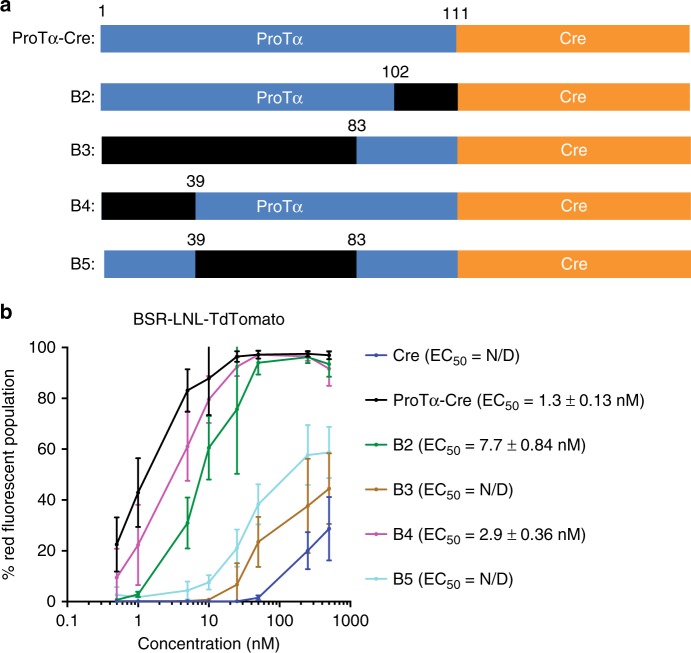
Truncation of ProTα. **a** Primary structure of several truncated variants of ProTα-Cre. Deleted regions are shown in black. **b** Lipid-mediated delivery efficiency of Cre fused to each truncated ProTα variant into the BSR-TdTomato cell line. Removal of the central region containing stretches of acidic residues (B3, B5) results in a >100-fold reduction in protein delivery potency. Deletion of either the N-terminus or the C-terminus of ProTα (B4, B2) also reduces delivery efficiency, suggesting that the termini also play a role in the delivery process. EC_50_ is defined as the protein concentration at which 50% of analyzed cells contain red fluorescence. Values and error bars represent the mean and standard deviation of three independent biological replicates performed on different days. EC_50_ values were determined by nonlinear fit to the Hill equation, and are followed by the standard errors of the fit. Source data are available in the Source Data file

To identify other domains within ProTα responsible for cationic lipid-mediated protein delivery, we generated a series of truncated ProTα variants and repeated the Cre fusion protein delivery titrations in the presence of RNAiMAX lipid (Fig. 3a).

**Fig. 4:**
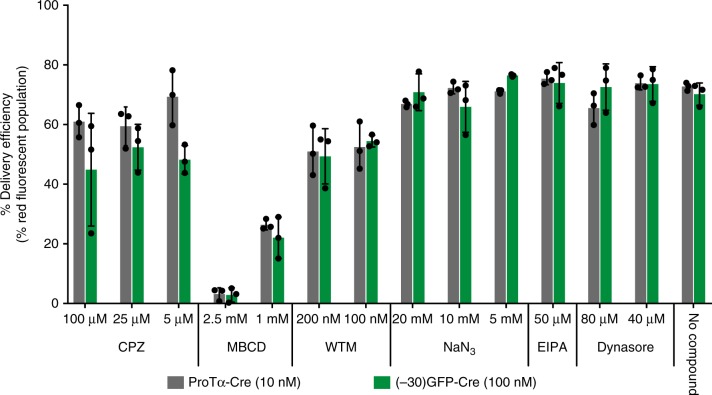
Effect of various endocytosis inhibitors on ProTα- and (–30)GFP-mediated lipid nanoparticle delivery. Cationic lipid nanoparticles combined with either 10 nM ProTα-Cre or 100 nM (–30)GFP-Cre were incubated with HEK293-RFP cells that had been pre-treated with inhibitors that block various endocytosis pathways. After 2 day, delivery efficiency was measured via flow cytometry, and the percent of cells containing red fluorescence was quantified and normalized against a control sample lacking any inhibitor. Chloropramazine (CPZ) and wortmannin (WTM) modestly decreased the delivery efficiency, whereas MBCD strongly blocked Cre recombination. Values and error bars represent the mean and standard deviation of three independent biological replicates performed on different days. Source data are available in the Source Data file

Deletion of the central anionic region almost completely abolished delivery enhancement by ProTα, consistent with the hypothesis that negative charges are critical for complexation with cationic lipids (Fig. 3b). Deletion of the N-terminal region, hypothesized to have receptor-binding properties^16^, also slightly increased (by 3-fold) the EC_50_ to 2.9 ± 0.39 nM, suggesting that ProTα might mediate liposome-cell membrane interaction via surface-exposed receptors, in addition to simple electrostatic charge attraction (Fig. 3b). The deletion of the C-terminal region resulted in an 8-fold impairment of delivery potency (EC_50_ = 7.7 ± 0.84 nM, Fig. 4b). The C-terminus of ProTα contains a putative NLS^16^. To test the possibility that this NLS is responsible for loss of delivery potency by ProTα lacking the C-terminus, we cloned, expressed, and delivered Cre containing an SV40 NLS. Cre delivery potency did not benefit from the addition of this NLS, consistent with the known ability of Cre to localize to the nucleus spontaneously, and suggesting that the C-terminal domain augments ProTα-mediated protein delivery potency through mechanisms beyond simply providing an additional nuclear localization signal (Supplementary Fig. 7). Taken together, these results indicate that multiple domains within ProTα contribute to its unusual cationic lipid-mediated protein delivery potency.

Next we probed potential mechanisms of ProTα-mediated protein delivery compared to those of canonical cationic liposomes, which are thought to enter cells through clathrin-dependent endocytosis^19,20^. We applied several inhibitors of various pathways involved in endocytosis and micropinocytosis to observe possible effects on delivery, including sodium azide, which depletes cellular ATP;^21^ ethylisopropyl amiloride (EIPA), a micropinocytosis inhibitor;^22^ Dynasore, a dynamin inhibitor;^23^ methyl-β-cyclodextrin (MBCD), a cholesterol-depleting molecule that prevents lipid raft formation;^24^ chloropramazine (CPZ), a clathrin-dependent endocytosis inhibitor^25^, and wortmannin (WTM), a PI3-kinase inhibitor that prevents clathrin-dependent endocytosis^26^. After pre-incubating each of these inhibitors with HEK293 cells containing the RFP Cre reporter, we combined either (–30)GFP-Cre or ProTα-Cre with RNAiMAX lipid and treated cells with the resulting complex for 4 h. Excess liposomes and inhibitors were removed using several washes with buffer containing heparin, and the cells were further incubated for 2 days.

Flow cytometry analysis showed that, in HEK293-RFP cells, MBCD almost completely negated cytosolic access, while CPZ and WTM caused mild inhibition of delivery (Fig. 4). These findings are consistent with those of a previous study^27^ that examined the mechanism of functional siRNA delivery using cationic lipids and implicated direct liposome fusion, which is strongly inhibited by MBCD, rather than clathrin-mediated endocytosis followed by endosomal escape, as the major source of functional cargo in the cytoplasm. As endocytosis inhibitors are known to widely differ in their degree of delivery inhibition across different cell types^25^, further studies are needed to conclusively implicate a mechanism for cationic nanoparticle protein delivery. Moreover, (–30)GFP-Cre and ProTα-Cre were inhibited by the same molecules to nearly identical degrees, suggesting that the large delivery potency increase enabled by ProTα is predominantly due to its apparent ability to engage cationic lipids at ~10-fold lower concentrations than (–30)GFP (Fig. 4). Minimal cell toxicity was observed for the inhibitors and doses used, other than moderate toxicity from sodium azide (Supplementary Fig. 8).

### Delivery of zinc-finger nucleases

Next we tested the ability of ProTα to enhance the lipid-mediated delivery of zinc-finger nucleases (ZFNs), chimeric genome editing proteins composed of a modular DNA-binding zinc-finger domains and a heterodimeric FokI nuclease^28^. Two ZFNs (left and right) when targeted to adjacent half-sites of a genomic locus will bind together, enabling their fused FokI nuclease domains to dimerize and induce a double stranded DNA cut, initiating end-joining processes that result in indels at the target locus^28^. ZFNs are promising research tools and therapeutics, and are in multiple clinical trials for the treatment of diseases including HIV infection^29^.

**Fig. 5:**
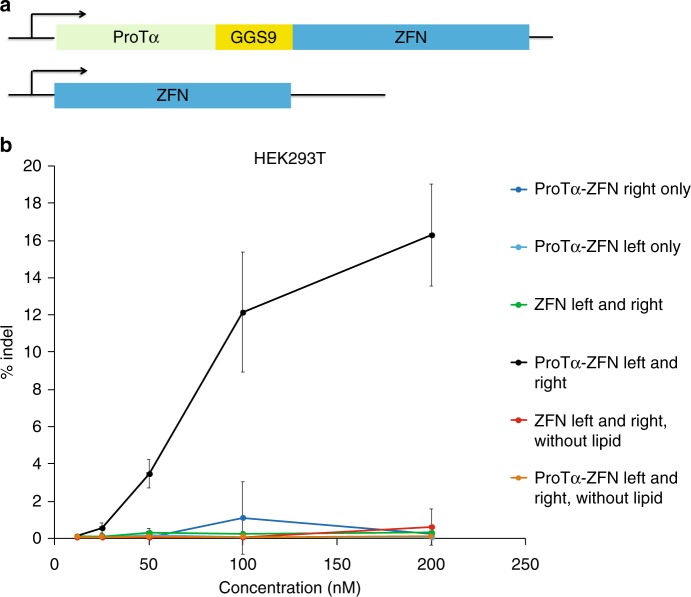
Delivery of zinc-finger nucleases (ZFNs) using ProTα. **a** Structure of ProTα-fused ZFN versus ZFN alone. **b** ProTα-fused ZFNs or unfused ZFNs targeting the *AAVS1* site in HEK293T cells were delivered using Lipofectamine RNAiMAX. ProTα enables efficient delivery of ZFNs and induce indels at a mid-nanomolar concentrations. Both left and right ZFN components, as well as lipid, are required for efficient indel generation. Values and error bars represent the mean and standard deviation of three independent biological replicates performed on different days. Source data are available in the Source Data file

ZFNs have been shown to enter cells spontaneously at high concentrations under some conditions^30^. We sought to test the ability of ProTα to mediate potent delivery of ZFNs with cationic lipids. We expressed both left and right ZFNs targeting the *AAVS1* safe harbor site in the human genome fused with ProTα at the N-terminus (Fig. 5a). Before conducting protein delivery, we confirmed that ProTα did not affect the activity of ZFNs in DNA cleavage assays in vitro conducted using purified substrates (Supplementary Fig. 9a), and also did not affect in HEK293T editing levels following plasmid transfection of ZFN variants (Supplementary Fig. 9b). We optimized the concentration of protein and lipid for ZFN delivery into HEK293T cells (Supplementary Fig. 10).

Finally, we delivered ProTα–ZFN fusions or unmodified ZFNs complexed with RNAiMAX lipid into HEK293T cells in the presence of 10% serum and measured the resulting levels of *AAVS1* target site genome editing. After 2 days, high-throughput sequencing (HTS) showed substantial ZFN-mediated indel formation at the target site only in cells treated with both pairs of ProTα–ZFN fusions and lipid in the mid-nM concentration regime (Fig. 5b). In contrast, cells treated with ZFNs lacking ProTα complexed with RNAiMAX resulted in no significant levels of genome editing (Fig. 5b). Neither the ZFNs alone nor the ProTα–ZFN fusions resulted in substantial cytotoxicity at the concentrations tested when complexed with RNAiMAX (Supplementary Fig. 11). Previously demonstrated self-delivery of ZFNs requires serum-free media and µM protein concentrations to generate moderate levels of indels, conditions that are prohibitive for some cell culture experiments and most in vivo applications^30^. Our result shows that ProTα can mediate delivery of ZFNs in cells media containing serum to generate even higher levels of indels using sub-µM concentrations of protein with a cationic lipid.

## Discussion

In this study we screened high anionic human proteins to identify ProTα, a small, intrinsically disordered protein that mediates efficient liposome-mediated delivery of fused cargo proteins. ProTα enables potent delivery of both Cre recombinase and ZFNs at nM concentrations into human cells when combined with a simple, commercially available cationic lipid. To our knowledge, ProTα represents the most potent protein reported to date that enables delivery of fused proteins via cationic liposomes. As ProTα expression is known in all human tissues tested^16^, it may serve as a less immunogenic domain for protein delivery than other non-human alternatives such as (–30)GFP.

Based on our previous work on the use of anionic proteins to mediate cationic lipid-based protein delivery^12,31^, we anticipate that ProTα will be compatible with a variety of lipid reagents, although optimizing the dose and concentrations of both the lipid and protein before delivery, as shown above, maximizes delivery potency. Reagent dose optimization is especially important as proteins, even when fused to ProTα, will vary in their ability to be encapsulated into cationic liposomes, and will also require different degrees of delivery depending on their desired function within the cell. We also envision that ProTα may be particularly enabling when delivering proteins with adverse properties that preclude naked protein delivery via conventional cell-penetrating peptides, that are not tolerated by the cell at higher concentrations, or that are difficult to generate in quantities needed for less-potent delivery methods.

## Methods

### Cloning

PCR was performed using Q5 Hot Start High-Fidelity DNA Polymerase (New England BioLabs). Candidate human protein DNAs were purchased from IDT as gBlock Gene Fragments. Bacterial expression plasmids encoding human protein fused to Cre were made using USER-cloning (New England BioLabs). Truncation of ProTα was done using blunt-end ligation to delete regions of ProTα. Following PCR, KLD enzyme mix (New England BioLabs) was used to phosphorylate and circularize the PCR product before transformation into NEB10beta cells (New England BioLabs).

### Protein expression

BL21 Star (DE3) chemically competent *E. coli* cells (ThermoFisher Scientific) were transformed with plasmids encoding the human proteins fused with Cre with a His_6_ C-terminal purification tag. A single colony was grown overnight in 2×YT broth containing 50 µg/ml Carbenicillin at 37 °C. The cells were diluted 1:20 into 1 L of the same media and grown until OD600 ~0.5. The cultures were incubated on ice for 60 min and protein expression was induced with 0.5 mM isopropyl-b-D-1-thiogalactopyranoside (IPTG, GoldBio Sciences). Protein was expressed for 14–16 h with shaking at 16 °C. Cells were centrifuged at 20,000 × *g* for 20 min, and then resuspended in a high salt buffer (100 mM tris(hydroxymethyl)-aminomethane (Tris)-HCl, pH 8.0, 1 M NaCl, 20% glycerol, 5 mM tris(2-carboxyethyl)phosphine (TCEP; GoldBio) with a protease inhibitor pellet (Roche). The cells were lysed using sonication and the supernatant was incubated with His-Pur nickel nitriloacetic acid (nickel-NTA) resin (ThermoFisher) with rotation at 4 °C for 30 min. The resin was washed with the high salt buffer before the protein was eluted with an elution buffer (high salt buffer supplemented with 200 mM imidazole). The eluent was purified on a 5 ml Hi-Trap Q (GE Healthcare) anion exchange column with an FPLC (AKTA Pure). The purified protein was quantified by a Pierce microplate BCA protein assay kit (Pierce Biotechnology) and snap-frozen in liquid nitrogen and stored at −80 °C until before use. ZFNs were purified as above with slight modification^30,32^. Briefly, the ZFN proteins were induced at 22 °C for 4 h with 0.7 mM IPTG. Buffers contained additional 0.1 mM ZnCl_2_ during Ni-column and FPLC purifications. Immediately after elution, the fractions were stored with 0.1 M L-Arg to improve protein stability. Amino acid sequences of proteins expressed in this study are provided in Supplementary Note 1.

### In vitro DNA cleavage assay

DNA containing the AAVS1 locus was amplified from purified HEK293T genomic DNA using PCR. ~350 bp PCR product was purified using Minelute columns (Qiagen). Hundred nanogram of DNA substrate was incubated with 300 nM of ZFN or ProTα-ZFN pairs in Cutsmart buffer with 1 mM Arginine and 100 µM ZnCl_2_ (New England Biolabs) at room temperature for 16 h. The cleavage product was detected by running the mixture on an agarose gel without further purification.

### Cell culture

HeLa-DsRed^6^, BSR-LNL-TdTomato^2^, HEK293-loxP-GFP-RFP (HEK293-RFP) (GenTarget), and HEK293T (ATCC) cells were cultured in Dulbecco’s Modified Eagle’s Medium plus GlutaMax (ThermoFisher Scientific) supplemented with 10% (v/v) FBS, at 37 °C with 5% CO_2_. Primary human fibroblasts (ThermoFisher Scientific C0045C) were cultured in Medium 106 (ThermoFisher Scientific M106500) supplemented with Low Serum Growth Supplement (ThermoFisher Scientific S00310) at 37 °C with 5% CO_2_.

### Protein delivery assays

For Cre delivery assays, proteins fused to Cre were serially diluted in OptiMem (ThermoFisher Scientific) at 12.5 µL volume. Equal volume of OptiMem containing 1.5 µL of Lipofectamine RNAiMAX (ThermoFisher Scientific) was mixed with the protein and incubated at 25 °C before delivery into cells that had been seeded on a 48-well collagen-coated BioCoat plate (Corning) at ~70% confluency (250 µL final volume). After 3 day, the cells were trypsinized using TrypLE reagent (ThermoFisher Scientific), and resuspended in culture media before being analyzed on the CytoFlex flow cytometer (Beckman Coulter). To facilitate quantitative comparison of delivery efficacy among human proteins, EC_50_ values were calculated by fitting the data for each protein to the Hill equation for proteins that resulted in greater than 50% Cre recombination at the doses assayed in the experiment.

For ZFN experiments, equimolar amounts of ‘left’ and ‘right’ ZFNs was diluted to 12.5 µL in OptiMem and was complexed with 12.5 µL of OptiMem containing 3.5 µL of Lipofectamine RNAiMAX (ThermoFisher Scientific). The resulting complex was delivered to cells that had been seeded on a 48-well collagen-coated BioCoat plate (Corning) at ~70% confluency at the final volume of 100 µL per well. After 4 h, the cells were incubated with fresh media for further 48 h. Then, the cells were lysed and DNA was purified DNA was isolated using the Agencourt DNAdvance Genomic DNA Isolation Kit (Beckman Coulter) according to the manufacturer’s instructions. AAVS1 site was amplified by PCR with flanking high-throughput sequencing primer pairs (Supplementary Table 1). Then, DNA was further amplified by PCR with primers containing Illumina sequencing adaptors. The products were gel-purified and quantified using KAPA Library Quantification Kit-Illumina (KAPA Biosystems). Samples were sequenced on an Illumina MiSeq following the manufacturer’s protocol. Indels were quantified within the 30-base window surrounding the cleavage site among the high-quality reads (Q > 30) using a previously reported Matlab script^33^.

For mCherry delivery assays into HEK293T and primary human fibroblasts, proteins fused to mCherry were serially diluted in OptiMem (ThermoFisher Scientific) at 12.5 µL volume. Equal volume of OptiMem containing 1.5 µL of Lipofectamine RNAiMAX (ThermoFisher Scientific) was mixed with the protein and incubated at 25 °C for 20 min before delivery into cells that had been seeded on a 48-well collagen-coated BioCoat plate (Corning) at 80% confluency (250 µL final volume). After 5 h, the cells were trypsinized using TrypLE reagent (ThermoFisher Scientific), and resuspended in culture media before being analyzed on the CytoFlex flow cytometer (Beckman Coulter).

### Inhibitor assays

HEK293-RFP cells at ~70% confluency were incubated with one of the following endocytosis inhibitors for 1 h: NaN_3_ (Sigma), 5-(N-ethyl-N-isopropyl)amiloride (EIPA, Santa Cruz Biotechnology), Dynasore (Abcam), Chlorpromazine (CPZ, Sigma), Methyl-β-cyclodextrin (MBCD, Sigma), Wortmannin (Sigma). Then, liposomes containing either (-30)GFP-Cre or ProTα-Cre were delivered and incubated at 37 °C with 5% CO_2_ for 4 h. The cells were washed three times with PBS containing heparin (Stem Cell Technologies) at 20 µg/ml. The cells were further recovered for 2 day, and the cells were analyzed by the CytoFlex flow cytometer (Beckman Coulter).

### Charge analog assays

To mimic the densely charged central region of ProTα, ‘DEEEE’ and ‘DEEE’ motifs were repeated until the appropriate amount of theoretical negative charge (−44 for (−44)poly D/E) was achieved. To enable the synthesis of the repeats as a gene fragment, the sequence was modified with serine, threonine, glycine, and leucine residues to eliminate sequence repetitiveness. (−30)poly D/E was created via C-terminal truncation of (−44)poly D/E to achieve a theoretical negative charge of −30. The charge variants were fused to the N-terminus of Cre via a (GGS)_9_ linker.

### ZFN transfections

HEK293T cells were plated on a 48-well collagen-coated BioCoat plate (Corning) 1 day prior to experiment. At ~70% confluency, 500 ng of “left” and “right” CMV-ZFNs and CMV-ProTα-ZFNs (1 µg total DNA content) were transfected using 1.5 µL Lipofectamine 2000 (ThermoFisher Scientific) according to the manufacturer’s protocol.

### Reporting summary

Further information on research design is available in the Nature Research Reporting Summary linked to this article.

## Supplementary information

Supplementary Information

Reporting Summary

Source data

## Data Availability

The source data underlying Figs. 1, 2a, b, 3a, b, 4, and 5b and Supplementary Figs. 1, 2, 4, 5, 6, 7, 8, 9a, b, 10, and 11 are provided as a Source Data file. High-throughput sequencing data generated during the current study have been deposited in the NCBI Sequence Read Archive repository under BioProject ID PRJNA507860. Other datasets generated during the current study are available from the corresponding author on reasonable request.
