# Supplementary material for: An anionic human protein mediates cationic liposome delivery of genome editing proteins into mammalian cells
Source: Nat Commun. 2019 Jul 2;10:2905. doi: 10.1038/s41467-019-10828-3 (PMC6606574; doi:10.1038/s41467-019-10828-3)
Supplement: Supplementary file 1 — Supplementary Information [file 41467_2019_10828_MOESM1_ESM.pdf]

# An Anionic Human Protein Mediates Cationic Liposome Delivery of Genome Editing Proteins into Mammalian Cells

Y. Bill Kim, Kevin T. Zhao, David B. Thompson, and David R. Liu

## SUPPLEMENTARY INFORMATION

Supplementary Figure 1. Identification of proteins from the human proteome with high negative charge-to-MW ratio.

Supplementary Figure 2. Representative flow cytometry analyses of HEK293-RFP Cre reporter cells treated with ProTα-Cre, (-30)GFP-Cre, or Cre at 0.25 nM and 5 nM concentrations.

Supplementary Figure 3. Microscopy of HEK293-RFP Cre reporter cells treated with ProTα-Cre, (-30)GFP-Cre, or Cre at 0.25 nM and 5 nM concentrations.

Supplementary Figure 4. Cytotoxicity of ProTα- and Lipofectamine RNAiMAX-mediated delivery of Cre to HEK293-RFP cells.

Supplementary Figure 5. Delivery of mCherry fluorescent protein into HEK293T cells.

Supplementary Figure 6. Delivery of mCherry fluorescent protein into human primary fibroblasts.

Supplementary Figure 7. Cre does not benefit from additional NLS.

Supplementary Figure 8. Cytotoxicity of HEK293-RFP cells with various endocytosis inhibitors after delivery of ProTα-Cre or (-30)GFP-Cre.

Supplementary Figure 9. Fusion of ProTα does not affect ZFN activity.

Supplementary Figure 10. Optimization of protein and lipid concentrations used for ZFN delivery.

Supplementary Figure 11. Cytotoxicity of ZFN protein delivery into HEK293T cells.

Supplementary Note 1. Amino acid sequences of proteins used in this study.

Supplementary Table 1. The list of primers used in this study.

Supplementary References

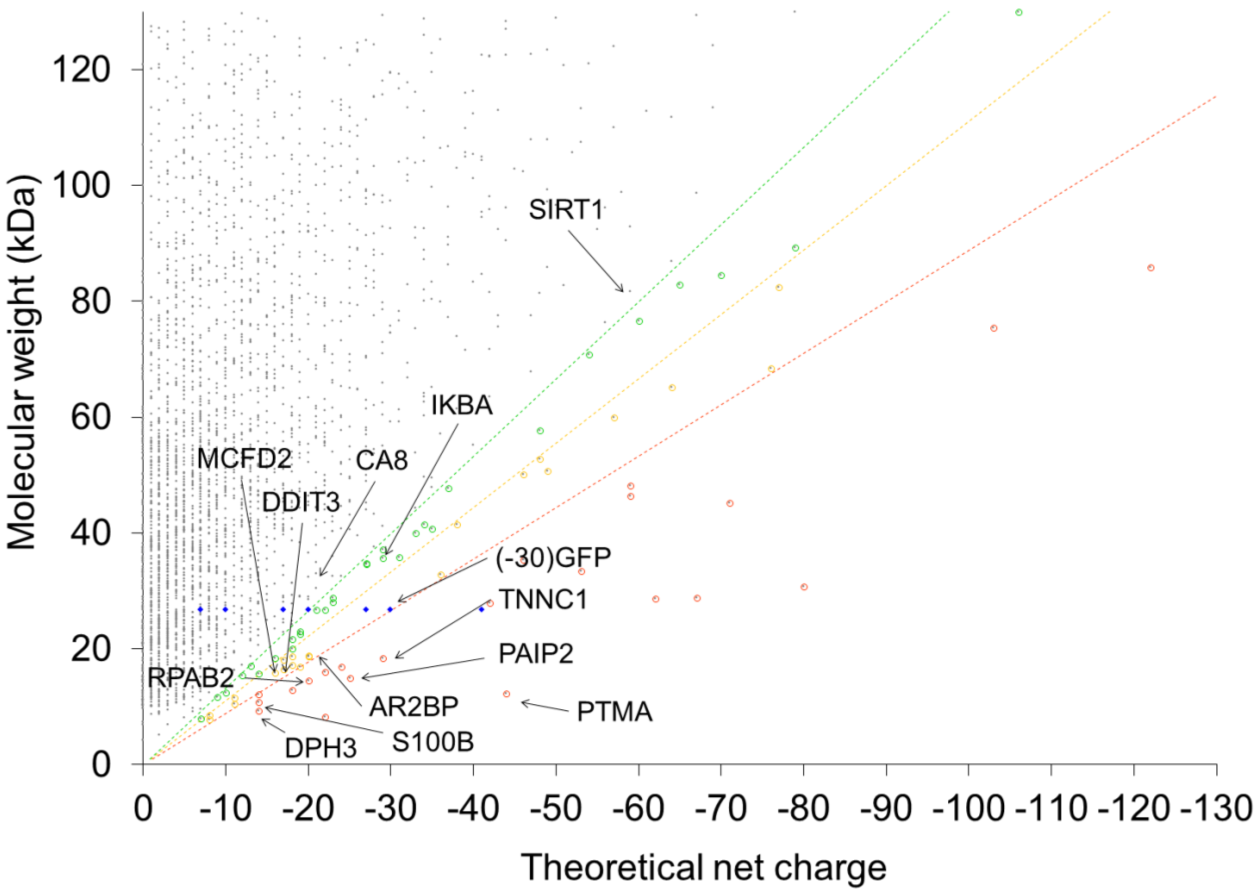


**Supplementary Figure 1. Identification of proteins from the human proteome with high negative charge-to-MW ratio.** Blue dots represent various engineered supercharged GFPs with -7, -10, -17, -20, -27, -30, and -40 theoretical net charges[1]. Green, yellow, and red lines represent charge-to-MW (kDa) ratios of 0.75, 0.9, and 1.1, respectively. Proteins with charge-to-MW ratio greater than 0.75 were considered for further study, as described in the main text.

[(-30)GFP-Cre] = 0.25 nM

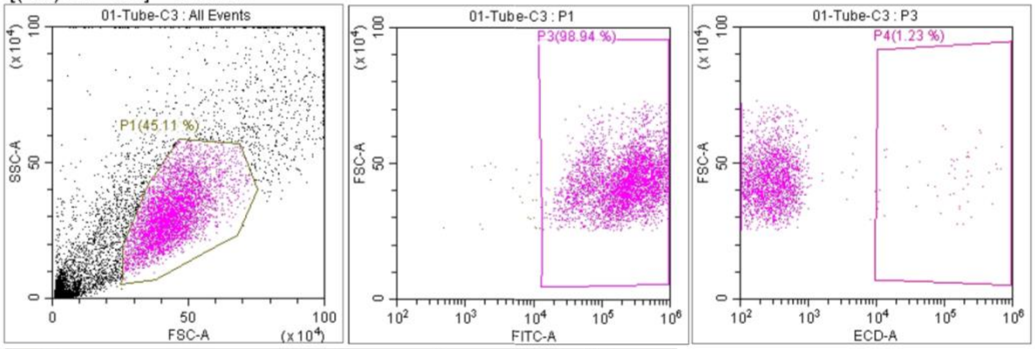


| Population | Events | % Total | % Parent |
|---|---|---|---|
| All Events | 10000 | 100.00 % | 100.00 % |
| P1 | 4511 | 45.11 % | 45.11 % |
| P3 | 4463 | 44.63 % | 98.94 % |
| P2 | 56 | 0.56 % | 1.25 % |
| P4 | 55 | 0.55 % | 1.23 % |

[ProTα-Cre] = 0.25 nM

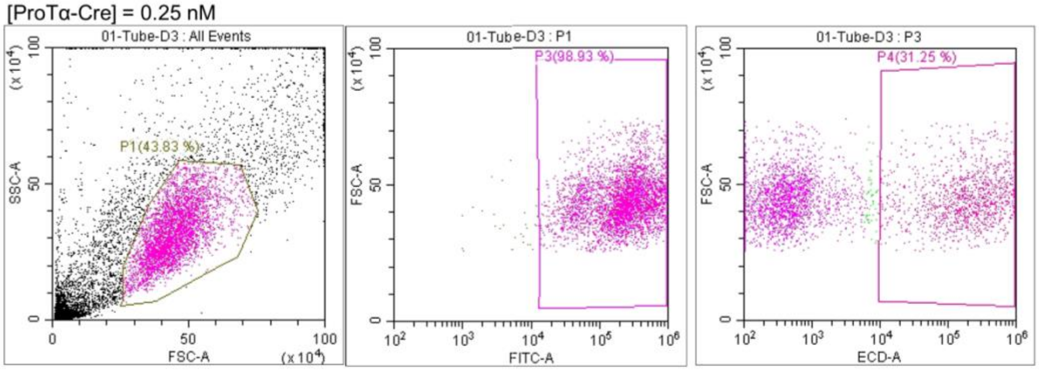


| Population | Events | % Total | % Parent |
|---|---|---|---|
| All Events | 10000 | 100.00 % | 100.00 % |
| P1 | 4383 | 43.83 % | 43.83 % |
| P3 | 4336 | 43.36 % | 98.93 % |
| P2 | 1405 | 14.05 % | 32.40 % |
| P4 | 1355 | 13.55 % | 31.25 % |

[Cre] = 0.25 nM

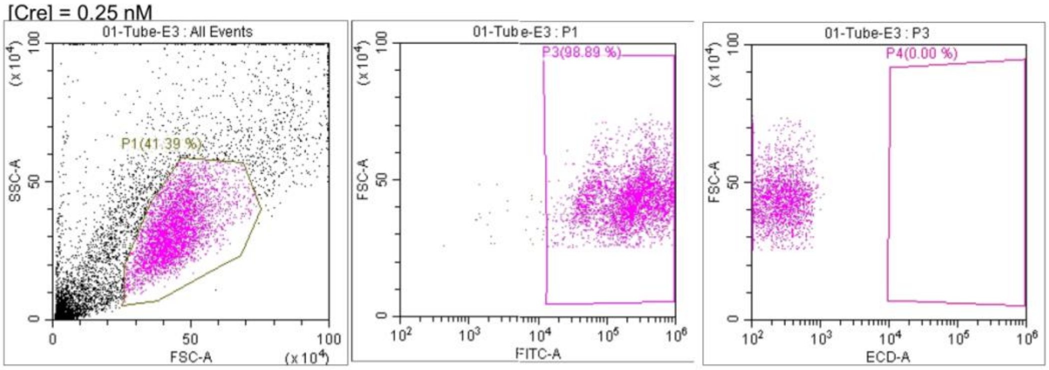


| Population | Events | % Total | % Parent |
|---|---|---|---|
| All Events | 10000 | 100.00 % | 100.00 % |
| P1 | 4139 | 41.39 % | 41.39 % |
| P3 | 4093 | 40.93 % | 98.89 % |
| P2 | 0 | 0.00 % | 0.00 % |
| P4 | 0 | 0.00 % | 0.00 % |

[(-30)GFP-Cre] = 5 nM

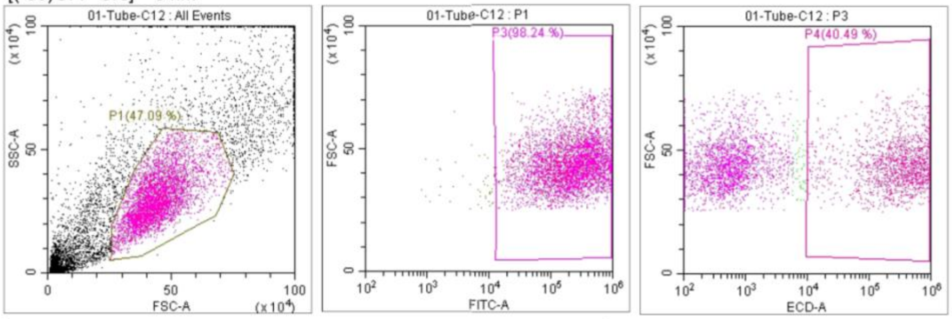


| Population | Events | % Total | % Parent |
|---|---|---|---|
| All Events | 10000 | 100.00 % | 100.00 % |
| P1 | 4709 | 47.09 % | 47.09 % |
| P3 | 4626 | 46.26 % | 98.24 % |
| P2 | 1929 | 19.29 % | 41.70 % |
| P4 | 1873 | 18.73 % | 40.49 % |

[ProTα-Cre] = 5 nM

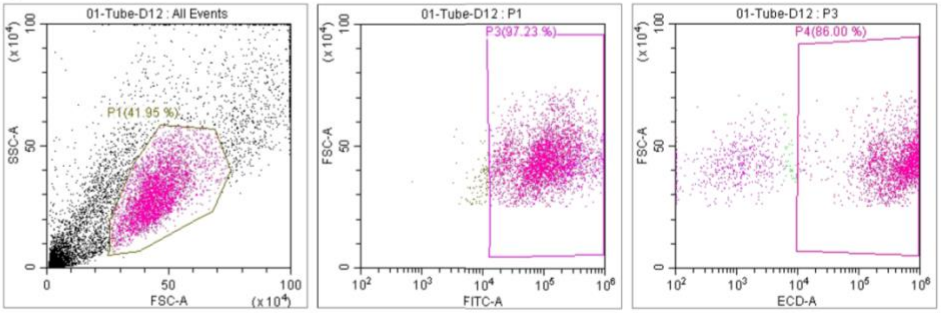


| Population | Events | % Total | % Parent |
|---|---|---|---|
| All Events | 10000 | 100.00 % | 100.00 % |
| P1 | 4195 | 41.95 % | 41.95 % |
| P3 | 4079 | 40.79 % | 97.23 % |
| P2 | 3541 | 35.41 % | 86.81 % |
| P4 | 3508 | 35.08 % | 86.00 % |

[Cre] = 5 nM

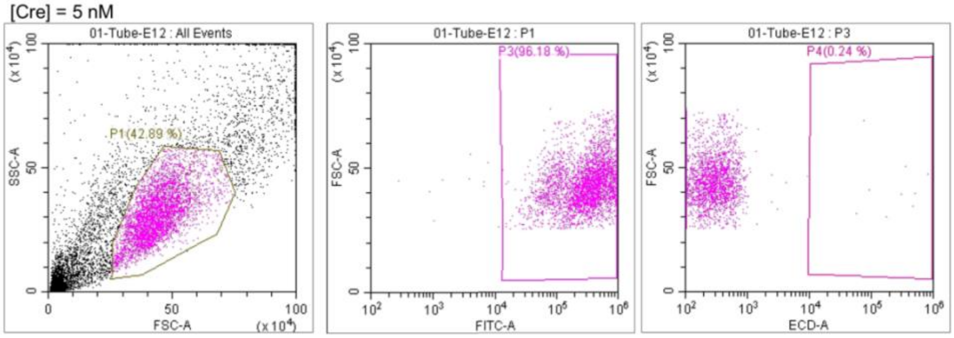


| Population | Events | % Total | % Parent |
|---|---|---|---|
| All Events | 10000 | 100.00 % | 100.00 % |
| P1 | 4289 | 42.89 % | 42.89 % |
| P3 | 4125 | 41.25 % | 96.18 % |
| P2 | 11 | 0.11 % | 0.27 % |
| P4 | 10 | 0.10 % | 0.24 % |

**Supplementary Figure 2. Representative flow cytometry analyses of HEK293-RFP Cre reporter cells treated with Lipofectamine RNAiMAX and ProTα-Cre, (-30)GFP-Cre, or Cre at 0.25 nM and 5 nM concentrations.** HEK293-RFP constitutively expresses GFP, which is gated for cell count using the FITC channel (excitation wavelength = 488 nm; emission collected at 525 nm with a 40-nm bandpass filter.) Red fluorescence is detected via the ECD channel (excitation wavelength = 561 nm; emission collected at 610 nm with a 20 nm bandpass filter.)

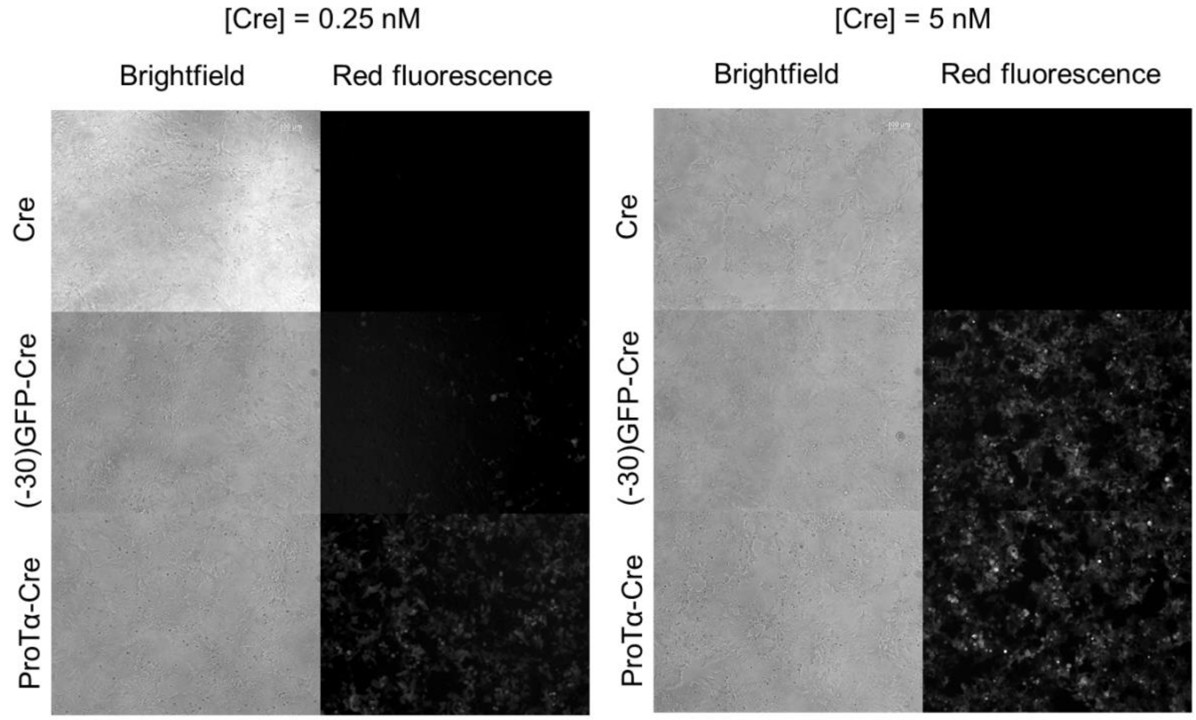


**Supplementary Figure 3. Microscopy of HEK293-RFP Cre reporter cells treated with Lipofectamine RNAiMAX and ProTα-Cre, (-30)GFP-Cre, or Cre at 0.25 nM and 5 nM concentrations.** While both ProTα-Cre and (-30)GFP-Cre facilitate lipid-mediated delivery of Cre at 5 nM, only ProTα-Cre enables detectable delivery at 0.25 nM protein concentration.

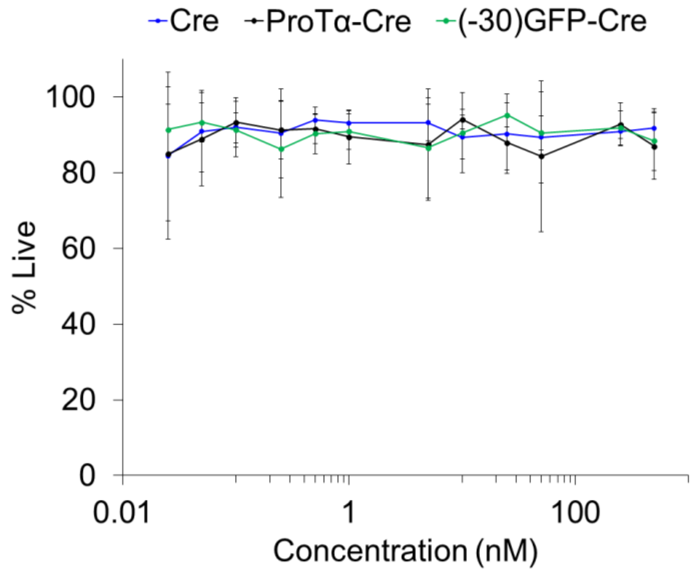


**Supplementary Figure 4. Cytotoxicity of ProTα- and Lipofectamine RNAiMAX-mediated Cre delivery to HEK293-RFP cells.** Trypan blue was used to quantify the percentage of live cells at the end of the experiment following incubation for 3 days with each of the three protein constructs shown at a variety of concentration. Minimal toxicity was observed in all samples. Values and error bars represent the mean and standard deviation of three independent biological replicates performed on different days.

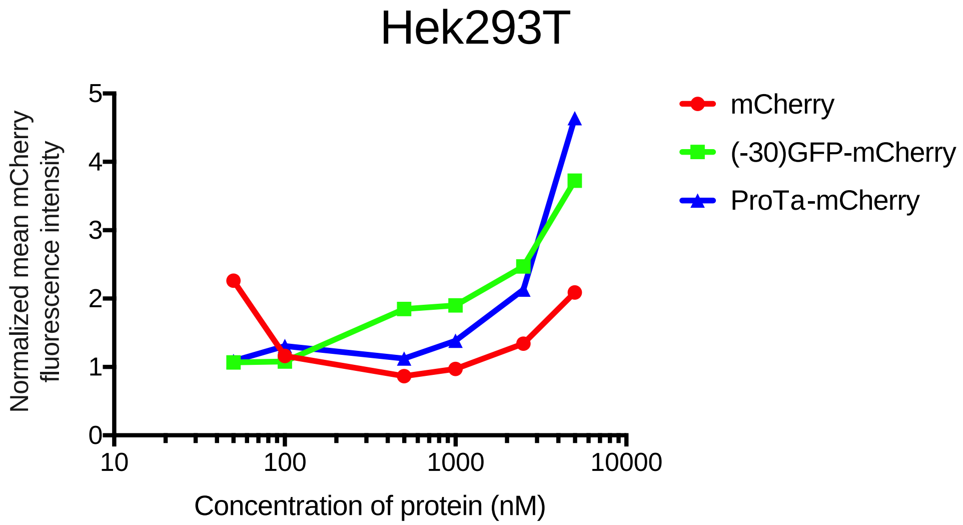


**Supplementary Figure 5. Delivery of mCherry fluorescent protein into HEK293T cells.** Flow cytometry analysis of HEK293T cells treated with cationic lipid nanoparticles complexed with ProTα–mCherry, (-30)GFP–mCherry, or unfused mCherry. ProTα or (-30)GFP fusion resulted in increased mCherry delivery across a wide range of protein concentrations compared to unfused mCherry, normalized to the fluorescence of untreated HEK293T cells.

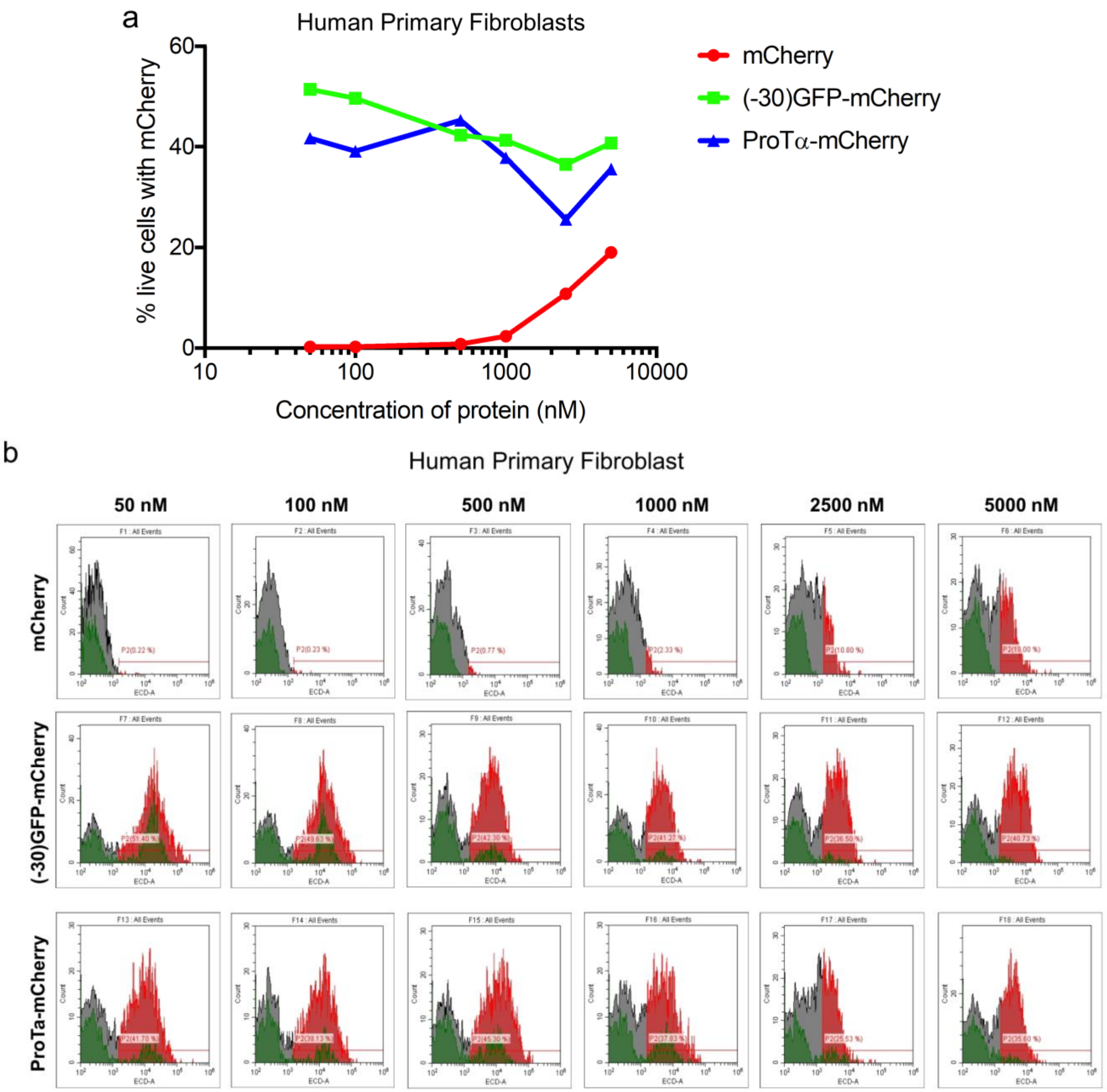


**Supplementary Figure 6. Delivery of mCherry fluorescent protein into human primary fibroblasts. a.** Flow cytometry analysis of human primary fibroblasts treated with cationic lipid nanoparticles complexed with ProTα–mCherry, (-30)GFP–mCherry, or unfused mCherry. ProTα or -30(GFP) fusion resulted in much more potent mCherry delivery compared to unfused mCherry. **b.** Flow cytometry plots of mCherry delivery into human primary fibroblasts. mCherry signal is gated using the ECD-A channel (excitation wavelength = 561 nm; emission collected at 610 nm with a 20 nm bandpass filter) Grey: all cells; green: live cells; red: cells with mCherry fluorescence.

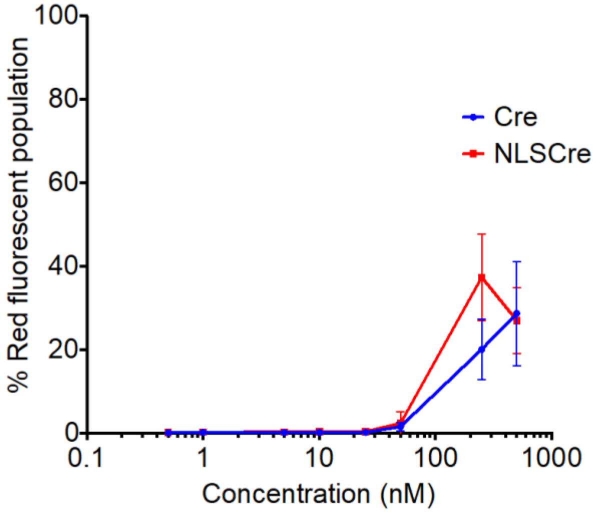


**Supplementary Figure 7. Cre does not benefit from an additional NLS.** Addition of an SV40 NLS to the N-terminus of Cre (NLSCre) does not significantly enhance the percentage of red fluorescent cells when delivered to HEK293-RFP cells using Lipofectamine RNAiMAX. Values and error bars represent the mean and standard deviation of three independent biological replicates performed on different days.

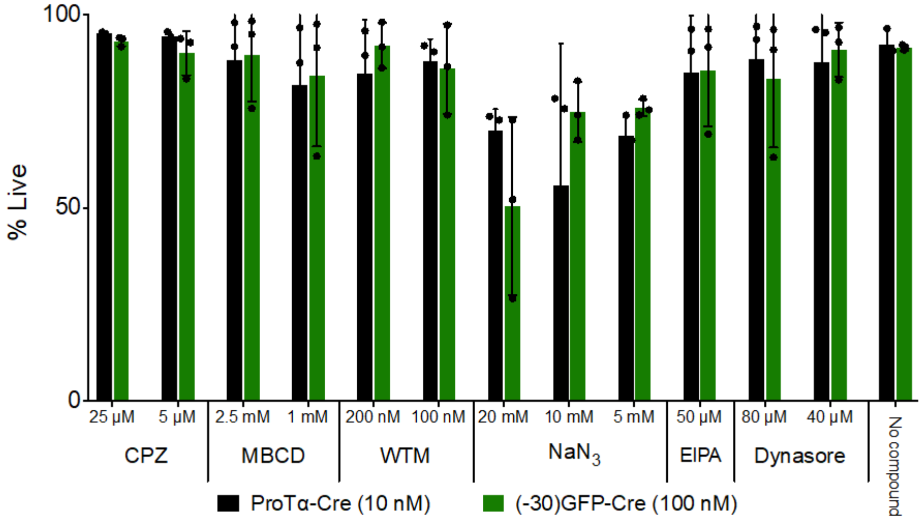


**Supplementary Figure 8. Cytotoxicity of HEK293-RFP cells with various endocytosis inhibitors after lipid-mediated delivery of ProTα-Cre or (-30)GFP-Cre.** Various endocytosis inhibitors were used as described in Methods. After the experiment, % live cells were determined by a trypan blue assay. At the doses tested inhibitors other than sodium azide did not result in substantial cell death. Values and error bars represent the mean and standard deviation of three independent biological replicates performed on different days.

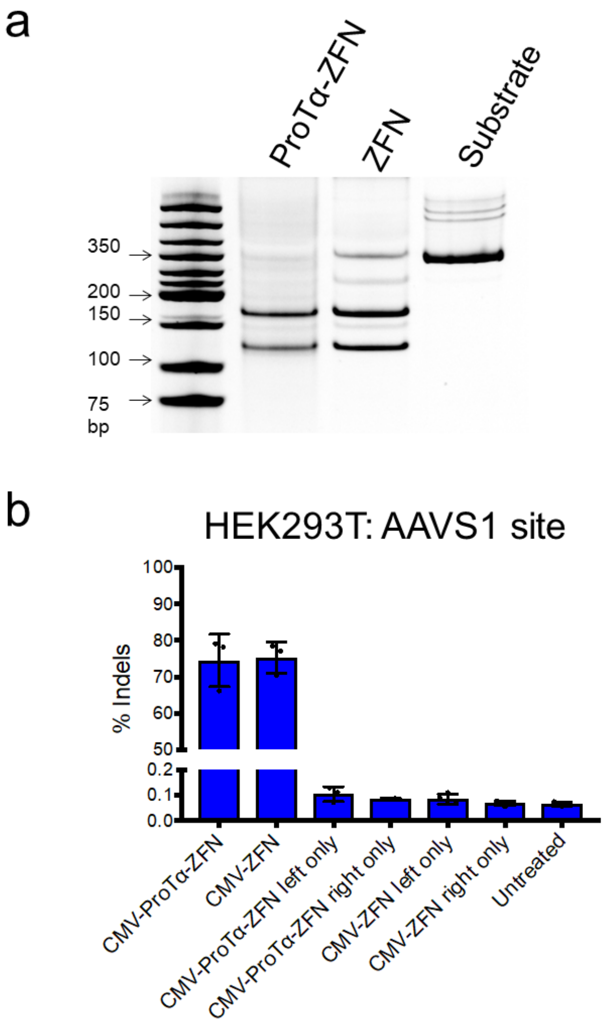


**Supplementary Figure 9. Fusion to ProTα does not impair ZFN activity. a.** *In vitro* DNA cleavage assay of ZFN and ProTα-ZFN on a purified DNA fragment containing a human AAVS1 sequence. **b.** Plasmid transfection of various constructs under constitutive CMV promoter in HEK293T cells. After 3 d, ZFN and ProTα-ZFN both induce comparable indels when delivered in DNA plasmid form. Values and error bars represent the mean and standard deviation of three independent biological replicates performed on different days.

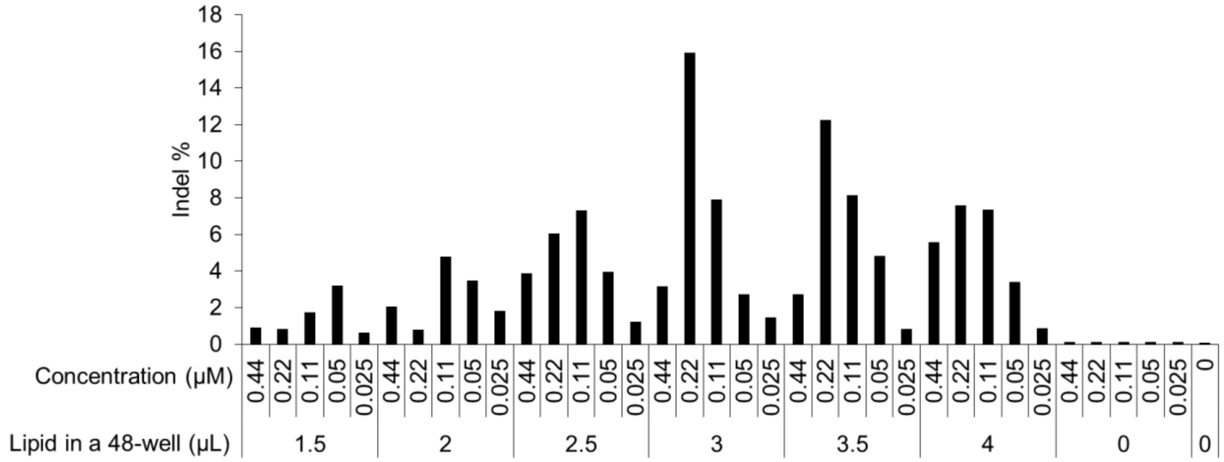


**Supplementary Figure 10. Optimization of protein and lipid concentrations used for ZFN delivery into HEK293T cells.** Various concentrations of left and right ProTα-ZFNs were complexed with a range of Lipofectamine RNAiMAX reagent amounts and incubated with HEK293T cells. After 2 d, indel percentage was quantified by HTS. Maximal indel was observed with protein concentration of ~200 nM and ~3 to 3.5 μL of lipid per well.

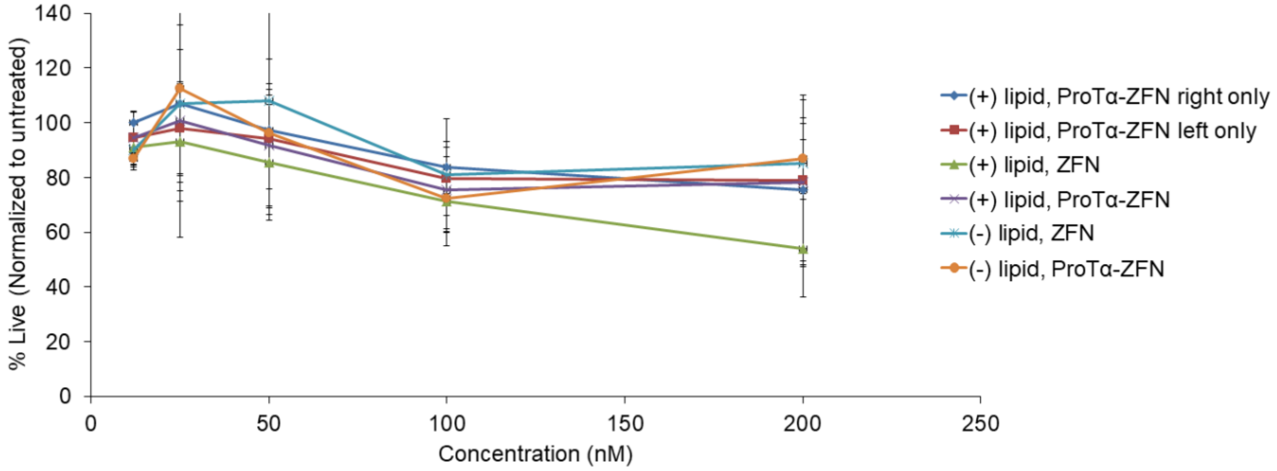


**Supplementary Figure 11. Cytotoxicity of ZFN protein delivery.** HEK293T cells were treated with ZFN variants with or without Lipofectamine RNAiMAX lipid. 2 d after treatment, Cell Titer Glo (Promega) was used to quantify the amount of live cells in each well, normalized to a well containing cells that received no treatment. Values and error bars represent the mean and standard deviation of three independent biological replicates performed on different days.

**Supplementary Note 1. Amino acid sequences of proteins used in this study.ProTα-Cre:**
MGASDAAVDTSSEITTKDLKEKKEVVEEAENGRDAPANGNAENEENGEQEADNEVDEEEE
EGGEEEEEEEEGDGEEEDGDEDEEAESATGKRAAEDDEDDDVDTKKQKTDEDDTGGSGG
SGGSGGSGGSGGSGGSGGSGGTASNLLTVHQNLPALPVDATSDEVRKNLMDMFRDRQAF
SEHTWKMLLSVCRSWAAWCKLNNRKWFPAEPEDVRDYLLYLQARGLAVKTIQQHLGQLNM
LHRRSGLPRPSDSNAVSLVMRRIRKENVDAGERAKQALAFERTDFDQVRSLMENSDRCQDI
RNLAFLGIAYNTLLRIAEIARIRVKDISRTDGGRMLIHIGRTKTLVSTAGVEKALSLGVTKLVER
WISVSGVADDPNNYLFCRVRKNGVAAPSATSQLSTRALEGIFEATHRLIYGAKDDSGQRYLA
WSGHSARVGAARDMARAGVSIPEIMQAGGWTNVNIVMNYIRNLDSETGAMVRLLEDGDGG
SHHHHHH

**(-30)GFP-Cre:**
MGASKGEELFDGVVPILVELDGDVNGHEFSVRGEGEGDATEGELTLKFICTTGELPVPWPTL
VTTLTYGVQCFSDYPDHMDQHDFFKSAMPEGYVQERTISFKDDGTYKTRAEVKFEGDTLVN
RIELKGIDFKEDGNILGHKLEYNFNSHDVYITADKQENGIKAEFEIRHNVEDGSVQLADHYQQ
NTPIGDGPVLLPDDHYLSTESALSKDPNEDRDHMVLLEFVTAAGIDHGMDELYKTGGSGGS
GGSGGSGGSGGSGGSGGSGGTASNLLTVHQNLPALPVDATSDEVRKNLMDMFRDRQAFS
EHTWKMLLSVCRSWAAWCKLNNRKWFPAEPEDVRDYLLYLQARGLAVKTIQQHLGQLNML
HRRSGLPRPSDSNAVSLVMRRIRKENVDAGERAKQALAFERTDFDQVRSLMENSDRCQDIR
NLAFLGIAYNTLLRIAEIARIRVKDISRTDGGRMLIHIGRTKTLVSTAGVEKALSLGVTKLVERWI
SVSGVADDPNNYLFCRVRKNGVAAPSATSQLSTRALEGIFEATHRLIYGAKDDSGQRYLAW
SGHSARVGAARDMARAGVSIPEIMQAGGWTNVNIVMNYIRNLDSETGAMVRLLEDGDGGS
HHHHHH

**Charge variants**
**(-30)PolyD/E-Cre:**
MGADEEESDEEELDEEEDELEEDEDTDEEGGDEELEDELDETGGSGGSGGSGGSGGSGG
SGGSGGSGGTASNLLTVHQNLPALPVDATSDEVRKNLMDMFRDRQAFSEHTWKMLLSVCR
SWAAWCKLNNRKWFPAEPEDVRDYLLYLQARGLAVKTIQQHLGQLNMLHRRSGLPRPSDS
NAVSLVMRRIRKENVDAGERAKQALAFERTDFDQVRSLMENSDRCQDIRNLAFLGIAYNTLL
RIAEIARIRVKDISRTDGGRMLIHIGRTKTLVSTAGVEKALSLGVTKLVERWISVSGVADDPNN
YLFCRVRKNGVAAPSATSQLSTRALEGIFEATHRLIYGAKDDSGQRYLAWSGHSARVGAAR
DMARAGVSIPEIMQAGGWTNVNIVMNYIRNLDSETGAMVRLLEDGDGGSHHHHHH

**(-44)PolyD/E-Cre:**
MGADEEESDEEELDEEEDELEEDEDTDEEGGDEELEDELDEDEETDDEESDEDEDEETGG
SGGSGGSGGSGGSGGSGGSGGSGGTASNLLTVHQNLPALPVDATSDEVRKNLMDMFRDR
QAFSEHTWKMLLSVCRSWAAWCKLNNRKWFPAEPEDVRDYLLYLQARGLAVKTIQQHLGQ
LNMLHRRSGLPRPSDSNAVSLVMRRIRKENVDAGERAKQALAFERTDFDQVRSLMENSDR
CQDIRNLAFLGIAYNTLLRIAEIARIRVKDISRTDGGRMLIHIGRTKTLVSTAGVEKALSLGVTKL
VERWISVSGVADDPNNYLFCRVRKNGVAAPSATSQLSTRALEGIFEATHRLIYGAKDDSGQR
YLAWSGHSARVGAARDMARAGVSIPEIMQAGGWTNVNIVMNYIRNLDSETGAMVRLLEDG
DGGSHHHHHH

**Truncated ProTα sequences**
**in B2:**
MGASDAAVDTSSEITTKDLKEKKEVVEEAENGRDAPANGNAENEENGEQEADNEVDEEEE
EGGEEEEEEEEGDGEEEDGDEDEEAESATGKRAAEDDEDDDVDT

**in B3:**
MGAESATGKRAAEDDEDDDVDTKKQKTDEDD

**in B4:**
MGAENEENGEQEADNEVDEEEEEGGEEEEEEEEEGDGEEEDGDEDEEAESATGKRAAEDD
EDDDVDTKKQKTDEDD

**in B5:**
MGASDAAVDTSSEITTKDLKEKKEVVEEAENGRDAPANGNAESATGKRAAEDDEDDDVDTK
KQKTDEDD

**NLSCre:**
MGAPKKKRKVSNLLTVHQNLPALPVDATSDEVRKNLMDMFRDRQAFSEHTWKMLLSVCRS
WAAWCKLNNRKWFPAEPEDVRDYLLYLQARGLAVKTIQQHLGQLNMLHRRSGLPRPSDSN
AVSLVMRRIRKENVDAGERAKQALAFERTDFDQVRSLMENSDRCQDIRNLAFLGIAYNTLLRI
AEIARIRVKDISRTDGGRMLIHIGRTKTLVSTAGVEKALSLGVTKLVERWISVSGVADDPNNYL
FCRVRKNGVAAPSATSQLSTRALEGIFEATHRLIYGAKDDSGQRYLAWSGHSARVGAARDM
ARAGVSIPEIMQAGGWTNVNIVMNYIRNLDSETGAMVRLLEDGDGGSHHHHHH

**ProTα-ZFN right:**
MGASDAAVDTSSEITTKDLKEKKEVVEEAENGRDAPANGNAENEENGEQEADNEVDEEEE
EGGEEEEEEEEEGDGEEEDGDEDEEAESATGKRAAEDDEDDDVDTKKQKTDEDDTGGSGG
SGGSGGSGGSGGSGGSGGSGGTAPKKKRKVGIHGVPAAMAERPFQCRICMRNFSQSSNL
ARHIRTHTGEKPFACDICGRKFARTDYLVDHTKIHTGSQKPFQCRICMRNFSYNTHLTRHIRT
HTGEKPFACDICGRKFAQGYNLAGHTKIHLRGSQLVKSELEEKKSELRHKLKYVPHEYIELIEI
ARNSTQDRILEMKVMEFFMKVYGYRGKHLGGSRKPDGAIYTVGSPIDYGVIVDTKAYSGGY
NLPIGQADEMQRYVKENQTRNKHINPNEWWKVYPSSVTEFKFLFVSGHFKGNYKAQLTRLN
HKTNCNGAVLSVEELLIGGEMIKAGTLTLEEVRRKFNNGEINFGGSHHHHHH

**ProTα-ZFN left:**
MGASDAAVDTSSEITTKDLKEKKEVVEEAENGRDAPANGNAENEENGEQEADNEVDEEEE
EGGEEEEEEEEEGDGEEEDGDEDEEAESATGKRAAEDDEDDDVDTKKQKTDEDDTGGSGG
SGGSGGSGGSGGSGGSGGSGGTAPKKKRKVGIHGVPAAMAERPFQCRICMRNFSYNWHL
QRHIRTHTGEKPFACDICGRKFARSDHLTTHTKIHTGSQKPFQCRICMRNFSHNYARDCHIR
THTGEKPFACDICGRKFAQNSTRIGHTKIHLRGSQLVKSELEEKKSELRHKLKYVPHEYIELIEI
ARNSTQDRILEMKVMEFFMKVYGYRGKHLGGSRKPDGAIYTVGSPIDYGVIVDTKAYSGGY
NLPIGQADEMERYVEENQTRNKHLNPNEWWKVYPSSVTEFKFLFVSGHFKGNYKAQLTRL
NHITNCNGAVLSVEELLIGGEMIKAGTLTLEEVRRKFNNGEINFGGSHHHHHH

**ZFN right:**
MGAPKKKRKVGIHGVPAAMAERPFQCRICMRNFSQSSNLARHIRTHTGEKPFACDICGRKF
ARTDYLVDHTKIHTGSQKPFQCRICMRNFSYNTHLTRHIRTHTGEKPFACDICGRKFAQGYN
LAGHTKIHLRGSQLVKSELEEKKSELRHKLKYVPHEYIELIEIARNSTQDRILEMKVMEFFMKV
YGYRGKHLGGSRKPDGAIYTVGSPIDYGVIVDTKAYSGGYNLPIGQADEMQRYVKENQTRN
KHINPNEWWKVYPSSVTEFKFLFVSGHFKGNYKAQLTRLNHKTNCNGAVLSVEELLIGGEMI
KAGTLTLEEVRRKFNNGEINFGGSHHHHHH

**ZFN left:**
MGAPKKKRKVGIHGVPAAMAERPFQCRICMRNFSYNWHLQRHIRTHTGEKPFACDICGRKF
ARSDHLTTHTKIHTGSQKPFQCRICMRNFSHNYARDCHIRTHTGEKPFACDICGRKFAQNST

RIGHTKIHLRGSQLVKSELEEKKSELRHKLKYVPHEYIELIEIARNSTQDRILEMKVMEFFMKV
YGYRGKHLGGSRKPDGAIYTVGSPIDYGVIVDTKAYSGGYNLPIGQADEMERYVEENQTRN
KHLNPNEWWKVYPSSVTEFKFLFVSGHFKGNYKAQLTRLNHITNCNGAVLSVEELLIGGEMI
KAGTLTLEEVRRKFNNGEINFGGSHHHHHH

**ProTα-mCherry**:
MGASDAAVDTSSEITTKDLKEKKEVVEEAENGRDAPANGNAENEENGEQEADNEVDEEEE
EGGEEEEEEEEEGDGEEEDGDEDEEAESATGKRAAEDDEDDDVDTKKQKTDEDDGGGGSG
GGGSMVSKGEEDNMAIIKEFMRFKVHMEGSVNGHEFEIEGEGEGRPYEGTQTAKLKVTKG
GPLPFAWDILSPQFMYGSKAYVKHPADIPDYLKLSFPEGFKWERVMNFEDGGVVTVTQDSS
LQDGEFIYKVKLRGTNFPSDGPVMQKKTMGWEASSERMYPEDGALKGEIKQRLKLKDGGH
YDAEVKTTYKAKKPVQLPGAYNVNIKLDITSHNEDYTIVEQYERAEGRHSTGGMDELYKHHH
HHH

**(-30)GFP-mCherry**:
MGASKGEELFDGVVPILVELDGDVNGHEFSVRGEGEGDATEGELTLKFICTTGELPVPWPTL
VTTLTYGVQCFSDYPDHMDQHDFFKSAMPEGYVQERTISFKDDGTYKTRAEVKFEGDTLVN
RIELKGIDFKEDGNILGHKLEYNFNSHDVYITADKQENGIKAEFEIRHNVEDGSVQLADHYQQ
NTPIGDGPVLLPDDHYLSTESALSKDPNEDRDHMVLLEFVTAAGIDHGMDELYKGGGGSGG
GGSMVSKGEEDNMAIIKEFMRFKVHMEGSVNGHEFEIEGEGEGRPYEGTQTAKLKVTKGG
PLPFAWDILSPQFMYGSKAYVKHPADIPDYLKLSFPEGFKWERVMNFEDGGVVTVTQDSSL
QDGEFIYKVKLRGTNFPSDGPVMQKKTMGWEASSERMYPEDGALKGEIKQRLKLKDGGHY
DAEVKTTYKAKKPVQLPGAYNVNIKLDITSHNEDYTIVEQYERAEGRHSTGGMDELYKHHHH
HH

## Supplementary Table 1. The list of primers used in this study.

| Name | Sequence | Use |
|---|---|---|
| AAVS-forward | ACACTCTTTCCCTACACGACGCTCTTCCGATCTNNNNGCCCTATGTCCACTTCAGGACAG | Amplify AAVS1 locus for HTS |
| AAVS-reverse | TGGAGTTCAGACGTGTGCTCTTCCGATCTAACCTTAGAGGTTCTGGCAA | Amplify AAVS1 locus for HTS |
| GBLK-forward | AGAAGGAGATATACCATGGGTGCT | USER cloning - amplify gblock fragment of human protein |
| GBLK-reverse | ACCAGAACCACCGCTACCACCGGT | USER cloning - amplify gblock fragment of human protein |
| PET-forward | AGCGGTGGTTCTGGTG | USER cloning - amplify pET vector backbone containing Cre |
| PET-reverse | ACCCATGGTATATCTCCTTCTT | USER cloning - amplify pET vector backbone containing Cre |
| B2-forward | ACCGGTGGTAGCGGTGGTTCTGGTGGTTCTGGTGGTAGCGG | Blunt-end cloning to make B2 |
| B2-reverse | GGTATCCACATCATCATCTTCGTCATCCTCTGCGGCACG | Blunt-end cloning to make B2 |
| B3-forward | GCCGAATCAGCTACGGGTAAACGTG | Blunt-end cloning to make B3 |
| B3-reverse | ACCCATGGTATATCTCCTTCTTAAAGTTAAACAAAATTATTTC | Blunt-end cloning to make B3 |
| B4-forward | GCCGAAAATGAAGAAAACGGCGAGCAG | Blunt-end cloning to make B4 |
| B4-reverse | ACCCATGGTATATCTCCTTCTTAAAGTTAAACAAAATTATTTC | Blunt-end cloning to make B4 |
| B5-forward | GCCGAATCAGCTACGGGTAAACGTG | Blunt-end cloning to make B5 |
| B5-reverse | GTTGCCGTTCGCCGGGGCATCACGCCCATTTTCCG | Blunt-end cloning to make B5 |

**Supplementary References**

1. Lawrence, M. S., Phillips, K. J. & Liu, D. R. Supercharging Proteins Can Impart Unusual Resilience. *Journal of the American Chemical Society* **129,** 10110–10112 (2007).
