# Supplementary material for: An anionic human protein mediates cationic liposome delivery of genome editing proteins into mammalian cells
Source: Nat Commun. 2019 Jul 2;10:2905. doi: 10.1038/s41467-019-10828-3 (PMC6606574; doi:10.1038/s41467-019-10828-3)
Supplement: Supplementary file 3 — Source data [file 41467_2019_10828_MOESM3_ESM.zip › Supplementary Figure 2/0.25nM -30GFPCre.pdf]

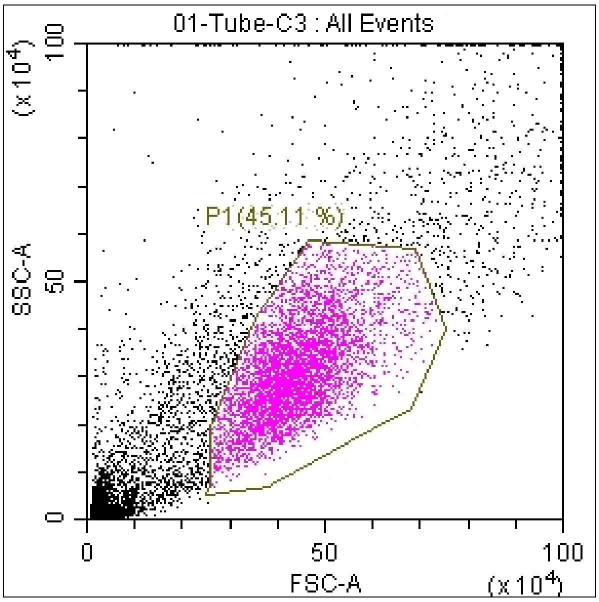

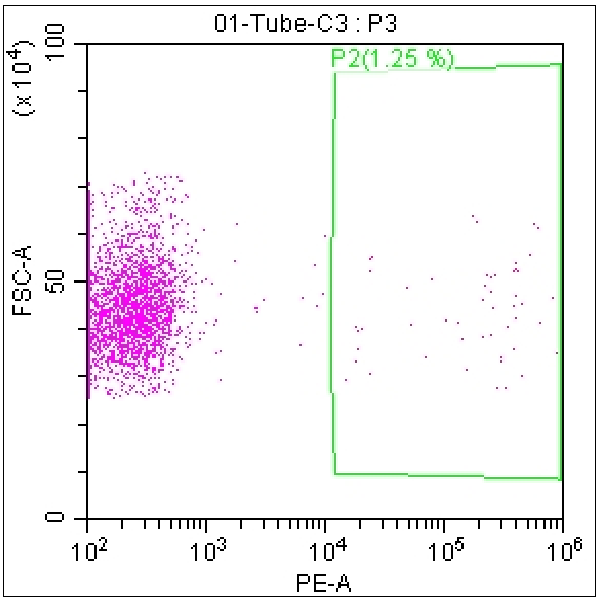

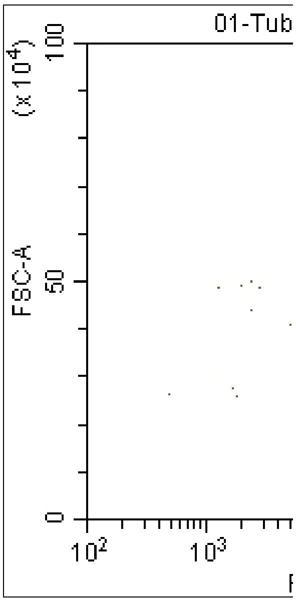

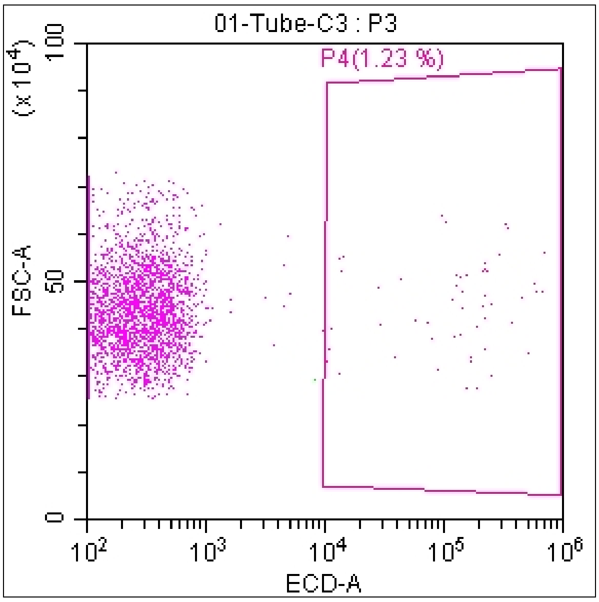

Tube Name: 01-Tube-C3

Sample ID:

Population	Events	% Total	% Parent
All Events	10000	100.00 %	100.00 %
P1	4511	45.11 %	45.11 %
P3	4463	44.63 %	98.94 %
P2	56	0.56 %	1.25 %
P4	55	0.55 %	1.23 %

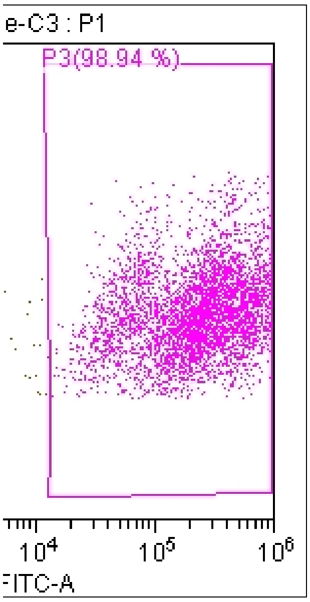

Tube Name: 01-Tube-C3

Sample ID:

Population	Events	% Total	% Parent	Mean FITC-A	Median FITC-A
All Events	10000	100.00 %	100.00 %	240788.6	74226.4
P2	56	0.56 %	1.25 %	463601.1	292473.6
P1	4511	45.11 %	45.11 %	343665.3	246607.9
P3	4463	44.63 %	98.94 %	335855.9	246633.2
P4	55	0.55 %	1.23 %	470827.7	293692.7
