# Supplementary material for: An anionic human protein mediates cationic liposome delivery of genome editing proteins into mammalian cells
Source: Nat Commun. 2019 Jul 2;10:2905. doi: 10.1038/s41467-019-10828-3 (PMC6606574; doi:10.1038/s41467-019-10828-3)
Supplement: Supplementary file 3 — Source data [file 41467_2019_10828_MOESM3_ESM.zip › Supplementary Figure 2/0.25nM Cre.pdf]

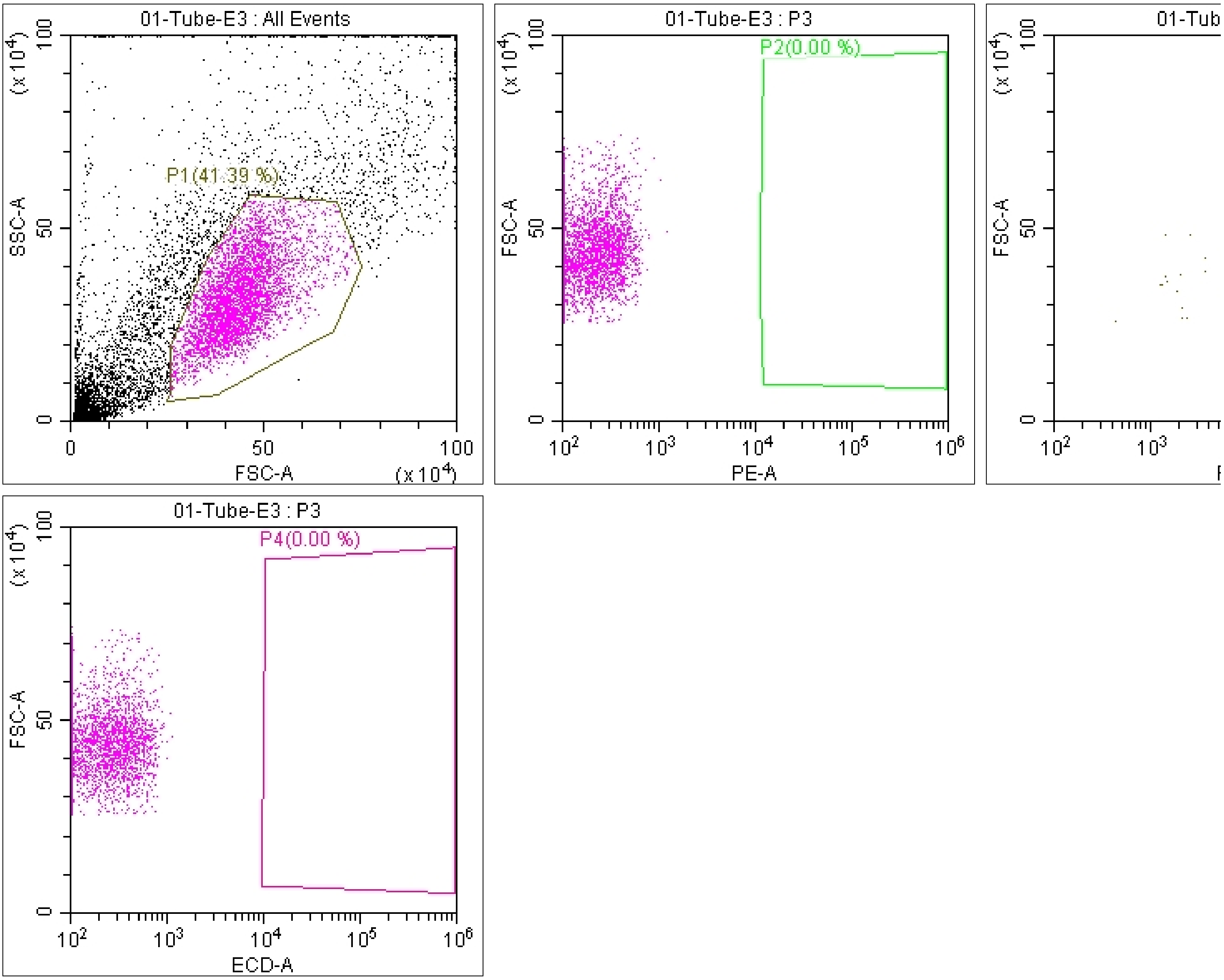

Tube Name: 01-Tube-E3

Sample ID:

Population	Events	% Total	% Parent
All Events	10000	100.00 %	100.00 %
P1	4139	41.39 %	41.39 %
P3	4093	40.93 %	98.89 %
P2	0	0.00 %	0.00 %
P4	0	0.00 %	0.00 %

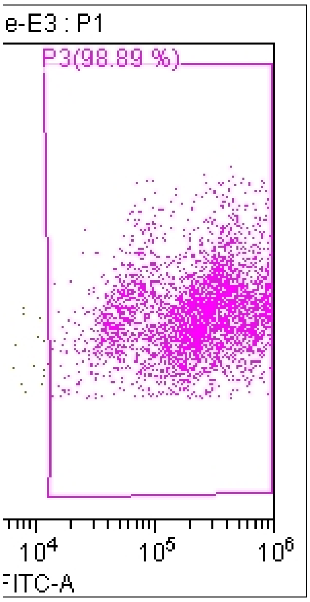

Tube Name: 01-Tube-E3
Sample ID:

Population	Events	% Total	% Parent	Mean FITC-A	Median FITC-A
All Events	10000	100.00 %	100.00 %	251004.0	60783.5
P2	0	0.00 %	0.00 %	####	####
P1	4139	41.39 %	41.39 %	361079.1	258320.6
P3	4093	40.93 %	98.89 %	355067.6	259572.7
P4	0	0.00 %	0.00 %	####	####
