# Supplementary material for: An anionic human protein mediates cationic liposome delivery of genome editing proteins into mammalian cells
Source: Nat Commun. 2019 Jul 2;10:2905. doi: 10.1038/s41467-019-10828-3 (PMC6606574; doi:10.1038/s41467-019-10828-3)
Supplement: Supplementary file 3 — Source data [file 41467_2019_10828_MOESM3_ESM.zip › Supplementary Figure 2/0.25nM ProTaCre.pdf]

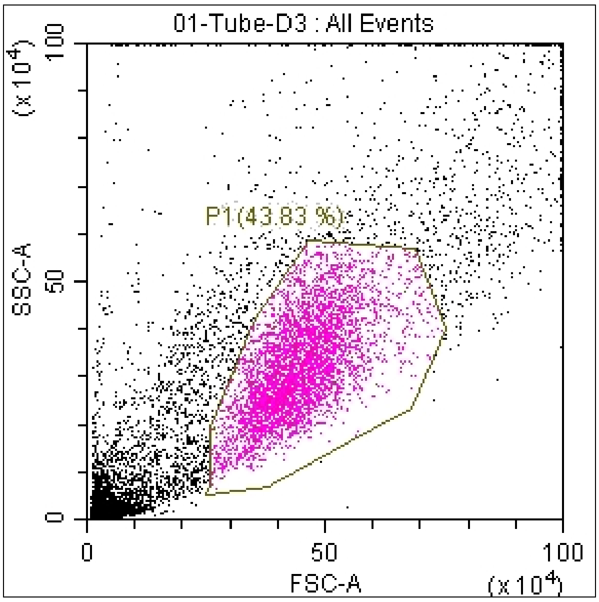

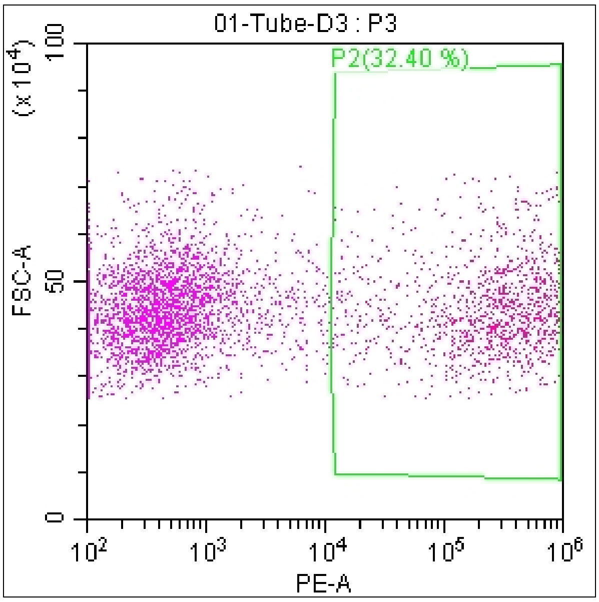

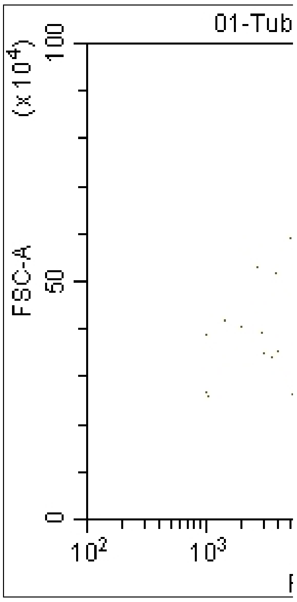

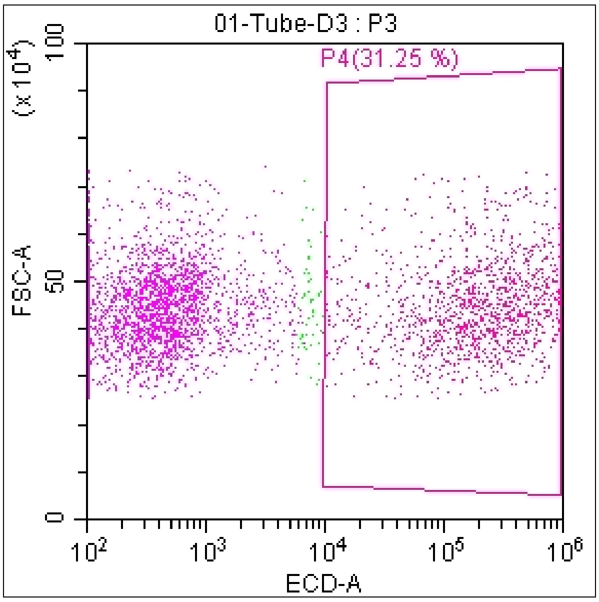

Tube Name: 01-Tube-D3

Sample ID:

Population	Events	% Total	% Parent
All Events	10000	100.00 %	100.00 %
P1	4383	43.83 %	43.83 %
P3	4336	43.36 %	98.93 %
P2	1405	14.05 %	32.40 %
P4	1355	13.55 %	31.25 %

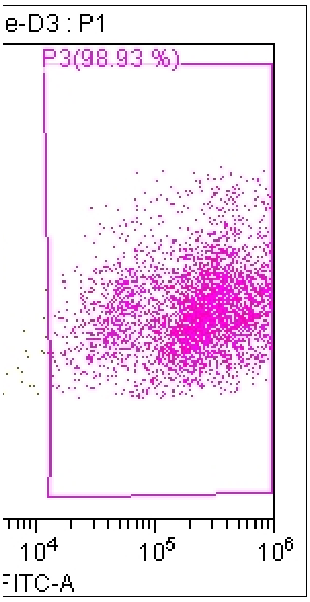

Tube Name: 01-Tube-D3

Sample ID:

Population	Events	% Total	% Parent	Mean FITC-A	Median FITC-A
All Events	10000	100.00 %	100.00 %	228920.0	66385.3
P2	1405	14.05 %	32.40 %	332842.7	236062.5
P1	4383	43.83 %	43.83 %	338726.6	244906.9
P3	4336	43.36 %	98.93 %	332227.2	245143.9
P4	1355	13.55 %	31.25 %	330758.2	235205.6
