# Supplementary material for: An anionic human protein mediates cationic liposome delivery of genome editing proteins into mammalian cells
Source: Nat Commun. 2019 Jul 2;10:2905. doi: 10.1038/s41467-019-10828-3 (PMC6606574; doi:10.1038/s41467-019-10828-3)
Supplement: Supplementary file 3 — Source data [file 41467_2019_10828_MOESM3_ESM.zip › Supplementary Figure 2/5nM -30GFPCre.pdf]

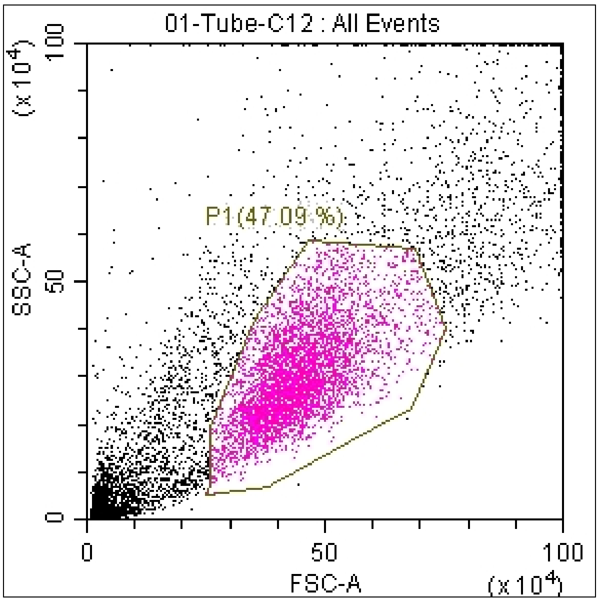

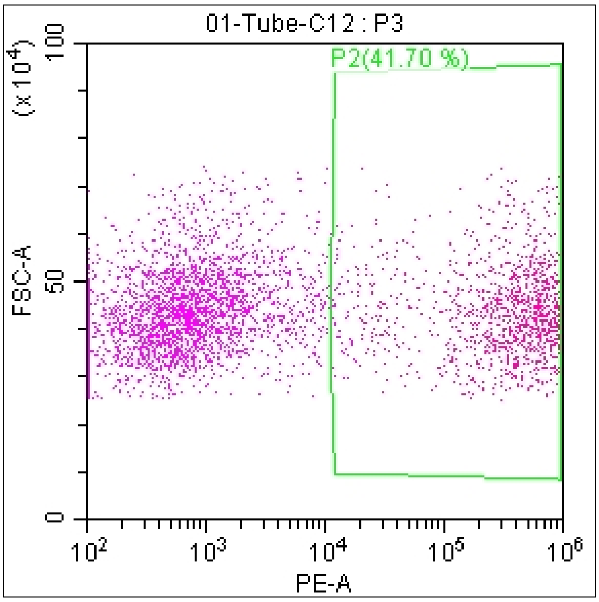

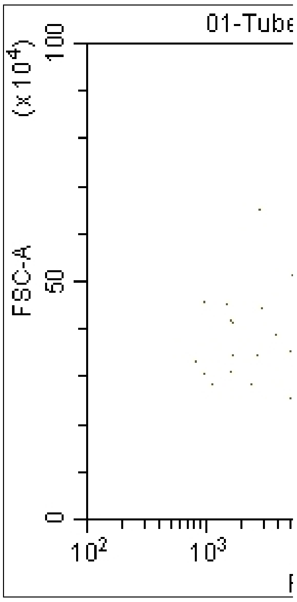

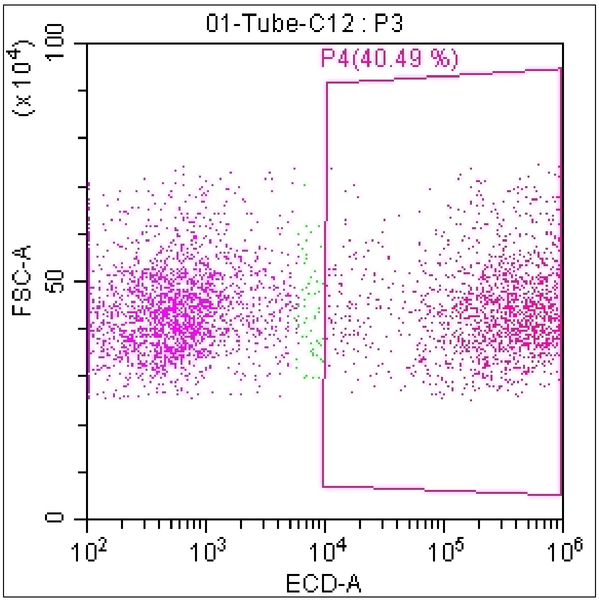

Tube Name: 01-Tube-C12

Sample ID:

Population	Events	% Total	% Parent
All Events	10000	100.00 %	100.00 %
P1	4709	47.09 %	47.09 %
P3	4626	46.26 %	98.24 %
P2	1929	19.29 %	41.70 %
P4	1873	18.73 %	40.49 %

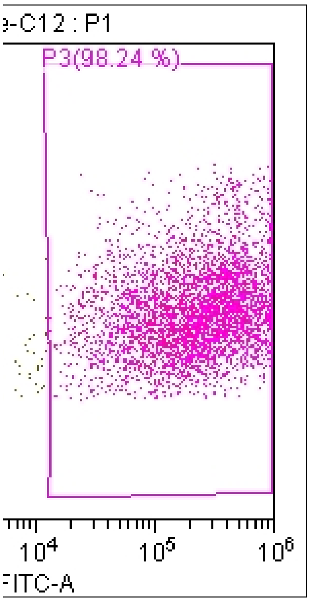

Tube Name: 01-Tube-C12

Sample ID:

Population	Events	% Total	% Parent	Mean FITC-A	Median FITC-A
All Events	10000	100.00 %	100.00 %	296077.1	108928.8
P2	1929	19.29 %	41.70 %	290805.1	189385.9
P1	4709	47.09 %	47.09 %	351665.8	247088.3
P3	4626	46.26 %	98.24 %	339046.7	247936.3
P4	1873	18.73 %	40.49 %	287698.3	187713.5
