# Supplementary material for: An anionic human protein mediates cationic liposome delivery of genome editing proteins into mammalian cells
Source: Nat Commun. 2019 Jul 2;10:2905. doi: 10.1038/s41467-019-10828-3 (PMC6606574; doi:10.1038/s41467-019-10828-3)
Supplement: Supplementary file 3 — Source data [file 41467_2019_10828_MOESM3_ESM.zip › Supplementary Figure 2/5nM Cre.pdf]

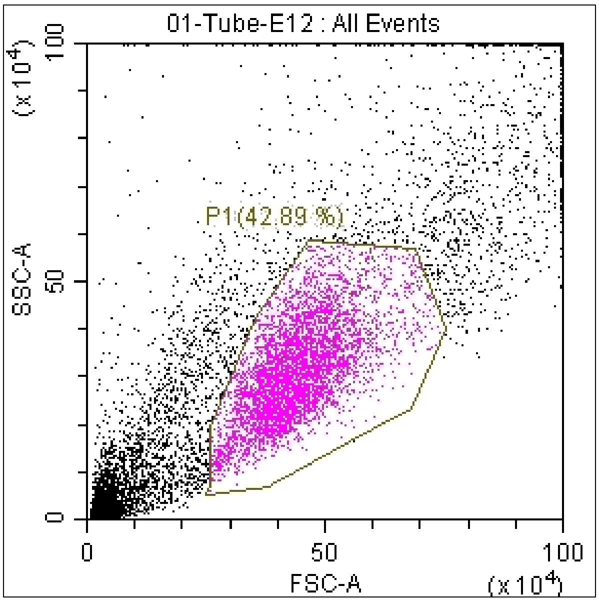

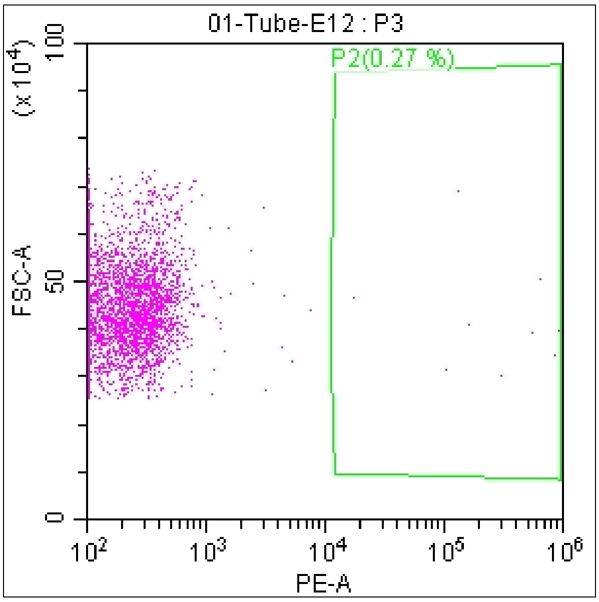

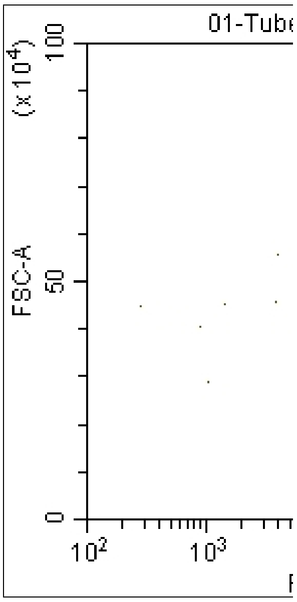

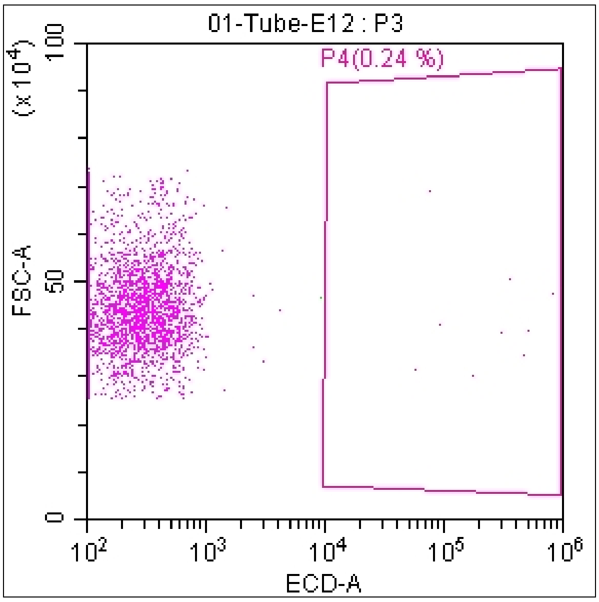

Tube Name: 01-Tube-E12

Sample ID:

Population	Events	% Total	% Parent
All Events	10000	100.00 %	100.00 %
P1	4289	42.89 %	42.89 %
P3	4125	41.25 %	96.18 %
P2	11	0.11 %	0.27 %
P4	10	0.10 %	0.24 %

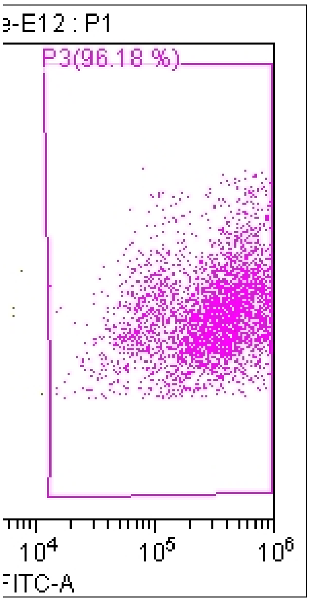

Tube Name: 01-Tube-E12

Sample ID:

Population	Events	% Total	% Parent	Mean FITC-A	Median FITC-A
All Events	10000	100.00 %	100.00 %	535272.4	188625.6
P2	11	0.11 %	0.27 %	395597.5	292491.3
P1	4289	42.89 %	42.89 %	556085.1	384008.3
P3	4125	41.25 %	96.18 %	487199.8	370911.6
P4	10	0.10 %	0.24 %	405908.2	276735.9
