# Supplementary material for: An anionic human protein mediates cationic liposome delivery of genome editing proteins into mammalian cells
Source: Nat Commun. 2019 Jul 2;10:2905. doi: 10.1038/s41467-019-10828-3 (PMC6606574; doi:10.1038/s41467-019-10828-3)
Supplement: Supplementary file 3 — Source data [file 41467_2019_10828_MOESM3_ESM.zip › Supplementary Figure 2/5nM ProTaCre.pdf]

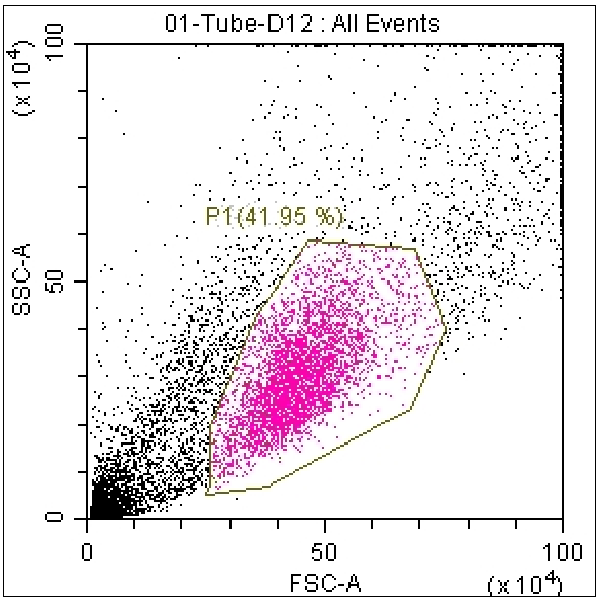

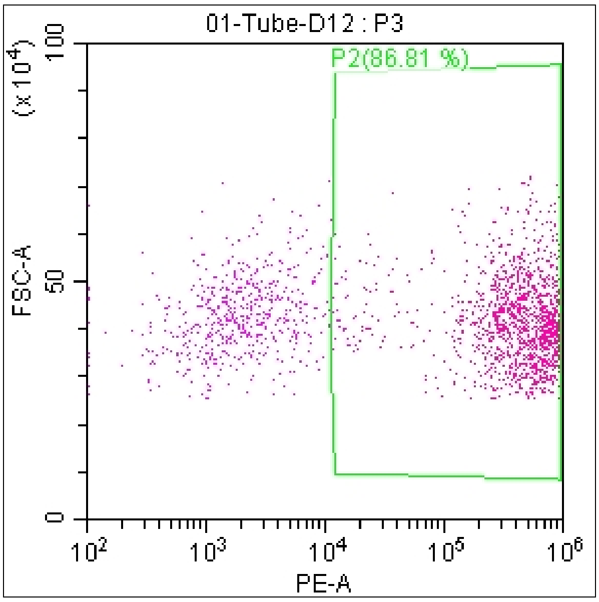

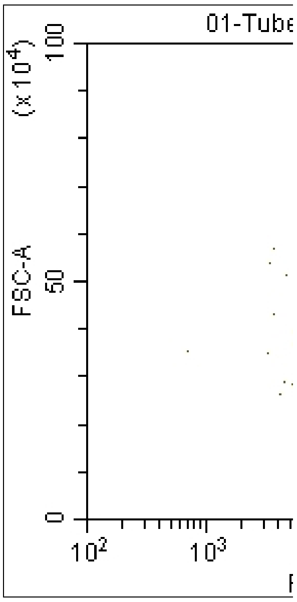

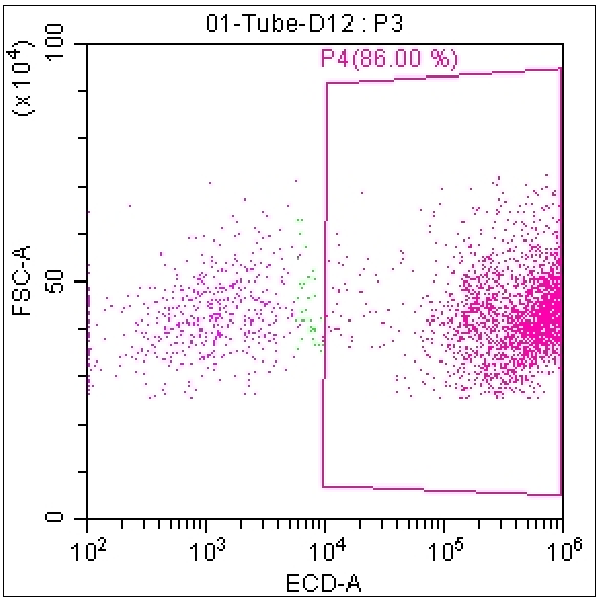

Tube Name: 01-Tube-D12

Sample ID:

Population	Events	% Total	% Parent
All Events	10000	100.00 %	100.00 %
P1	4195	41.95 %	41.95 %
P3	4079	40.79 %	97.23 %
P2	3541	35.41 %	86.81 %
P4	3508	35.08 %	86.00 %

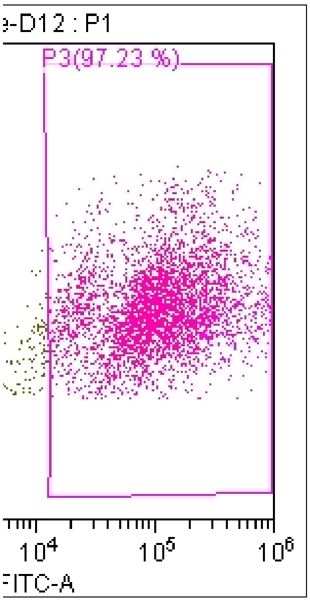

Tube Name: 01-Tube-D12
Sample ID:

Population	Events	% Total	% Parent	Mean FITC-A	Median FITC-A
All Events	10000	100.00 %	100.00 %	126443.7	34097.4
P2	3541	35.41 %	86.81 %	135538.7	97149.0
P1	4195	41.95 %	41.95 %	167662.6	106096.3
P3	4079	40.79 %	97.23 %	167521.9	108569.7
P4	3508	35.08 %	86.00 %	132988.6	96565.3
