# Supplementary material for: An anionic human protein mediates cationic liposome delivery of genome editing proteins into mammalian cells
Source: Nat Commun. 2019 Jul 2;10:2905. doi: 10.1038/s41467-019-10828-3 (PMC6606574; doi:10.1038/s41467-019-10828-3)
Supplement: Supplementary file 3 — Source data [file 41467_2019_10828_MOESM3_ESM.zip › Supplementary Figures 5 and 6/F0.pdf]

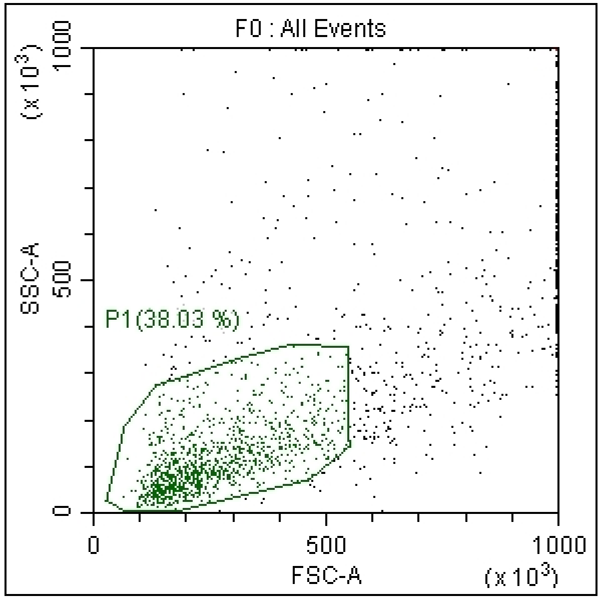

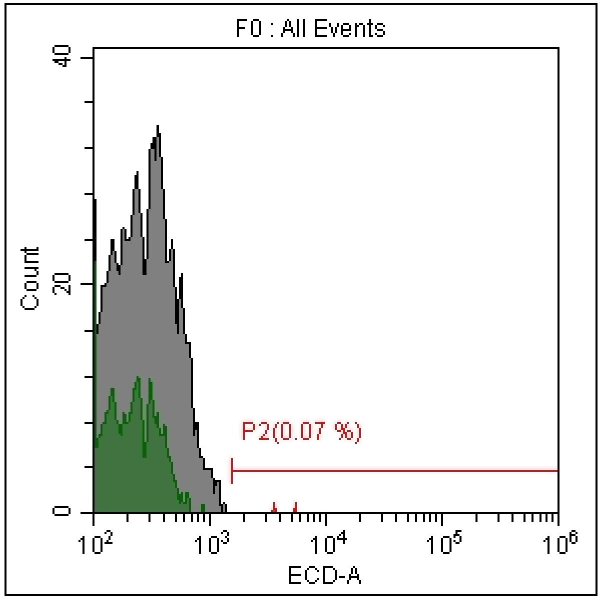

Experiment Name: KZ.20190422

Tube Name: F0

Sample ID:

Volume(μL): 93.2

Population	Mean FITC-A	Events	% Parent	Events/μL(V)	Median FITC-A	rCV FITC-A	...
All Events	13746.8	3000	100.00 %	32.19	2732.8	154.15 %	...
P2	368125.3	2	0.07 %	0.02	368125.3	51.63 %	...
P1	607.1	1141	38.03 %	12.24	448.4	145.55 %	...
