# Supplementary material for: An anionic human protein mediates cationic liposome delivery of genome editing proteins into mammalian cells
Source: Nat Commun. 2019 Jul 2;10:2905. doi: 10.1038/s41467-019-10828-3 (PMC6606574; doi:10.1038/s41467-019-10828-3)
Supplement: Supplementary file 3 — Source data [file 41467_2019_10828_MOESM3_ESM.zip › Supplementary Figures 5 and 6/F1.pdf]

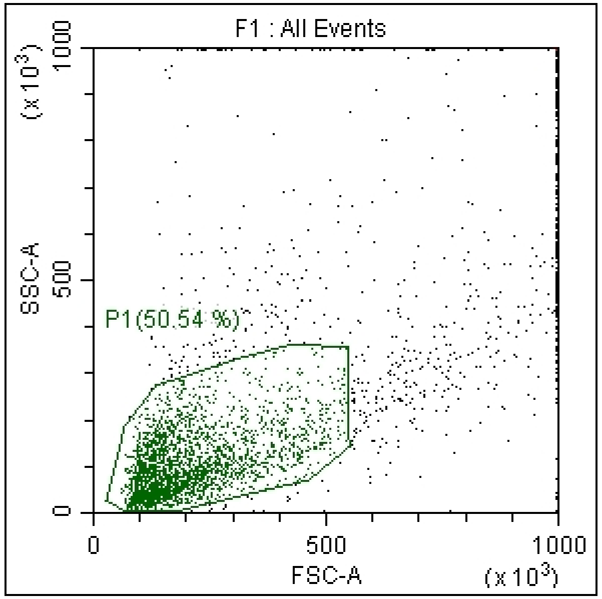

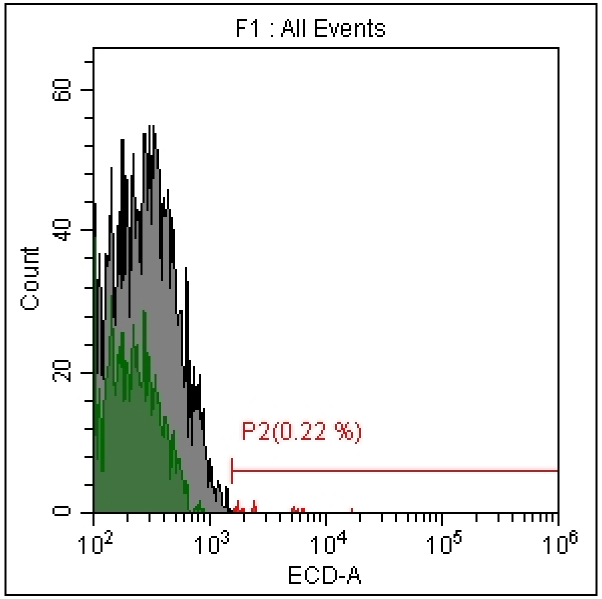

Experiment Name: KZ.20190422

Tube Name: F1

Sample ID:

Volume(µL): 294.2

Population	Mean FITC-A	Events	% Parent	Events/µL(V)	Median FITC-A	rCV FITC-A	...
All Events	12024.3	5000	100.00 %	17.00	1909.2	149.91 %	...
P2	419614.4	11	0.22 %	0.04	279694.8	96.29 %	...
P1	879.7	2527	50.54 %	8.59	690.0	128.86 %	...
