# Supplementary material for: An anionic human protein mediates cationic liposome delivery of genome editing proteins into mammalian cells
Source: Nat Commun. 2019 Jul 2;10:2905. doi: 10.1038/s41467-019-10828-3 (PMC6606574; doi:10.1038/s41467-019-10828-3)
Supplement: Supplementary file 3 — Source data [file 41467_2019_10828_MOESM3_ESM.zip › Supplementary Figures 5 and 6/F10.pdf]

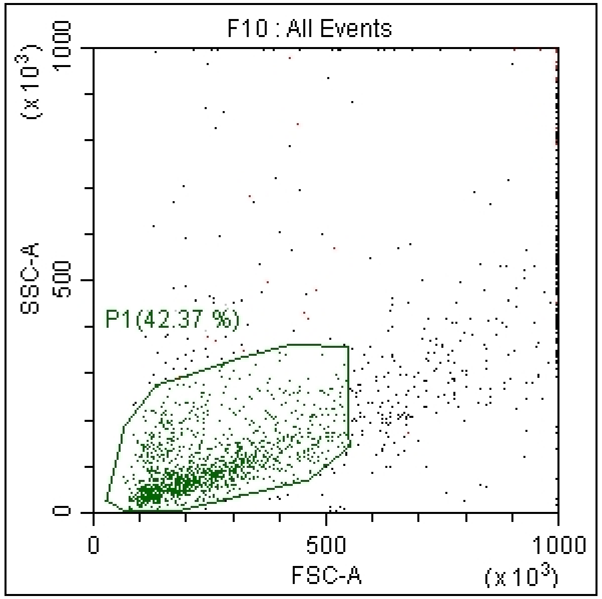

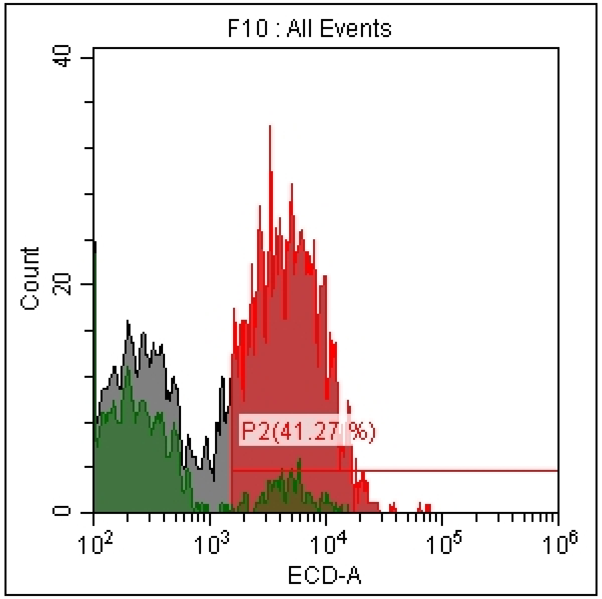

Experiment Name: KZ.20190422
Tube Name: F10
Sample ID:
Volume(µL): 219.0

Population	Mean FITC-A	Events	% Parent	Events/µL(V)	Median FITC-A	rCV FITC-A	...
All Events	40858.8	3000	100.00 %	13.70	5440.0	159.14 %	...
P2	94436.5	1238	41.27 %	5.65	50943.1	90.19 %	...
P1	19610.5	1271	42.37 %	5.80	739.4	134.20 %	...
