# Supplementary material for: An anionic human protein mediates cationic liposome delivery of genome editing proteins into mammalian cells
Source: Nat Commun. 2019 Jul 2;10:2905. doi: 10.1038/s41467-019-10828-3 (PMC6606574; doi:10.1038/s41467-019-10828-3)
Supplement: Supplementary file 3 — Source data [file 41467_2019_10828_MOESM3_ESM.zip › Supplementary Figures 5 and 6/F11.pdf]

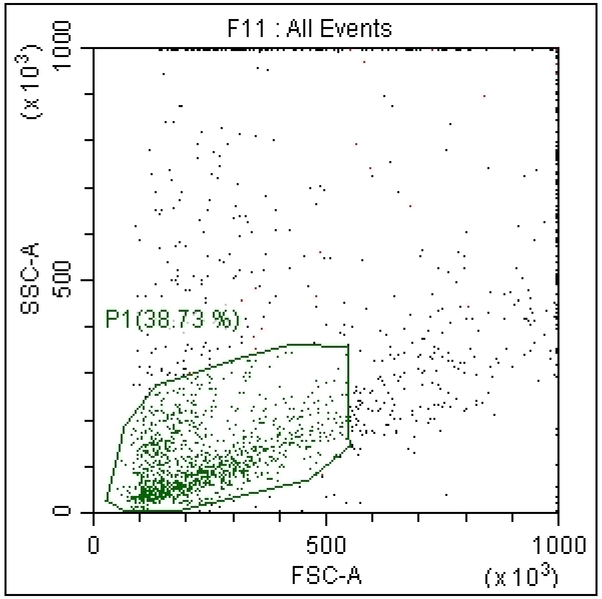

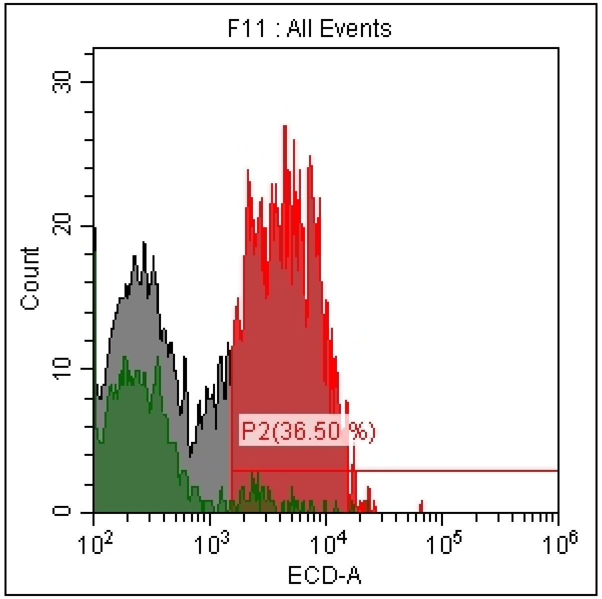

Experiment Name: KZ.20190422

Tube Name: F11

Sample ID:

Volume(μL): 147.9

Population	Parent Population	Mean ECD-A	Median ECD-A	rCV ECD-A	rSD ECD-A
All Events	####	2089.3	323.3	245.57 %	793.9
P2	All Events	5477.3	4424.6	67.00 %	2964.3
P1	All Events	193.5	33.6	727.40 %	244.4
