# Supplementary material for: An anionic human protein mediates cationic liposome delivery of genome editing proteins into mammalian cells
Source: Nat Commun. 2019 Jul 2;10:2905. doi: 10.1038/s41467-019-10828-3 (PMC6606574; doi:10.1038/s41467-019-10828-3)
Supplement: Supplementary file 3 — Source data [file 41467_2019_10828_MOESM3_ESM.zip › Supplementary Figures 5 and 6/F12.pdf]

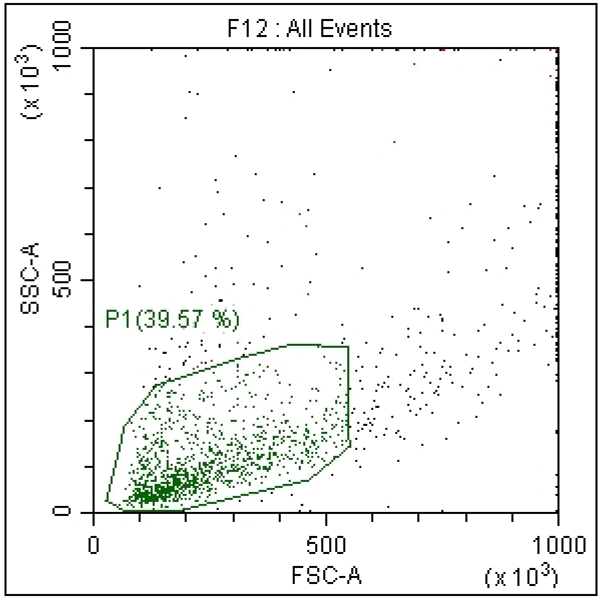

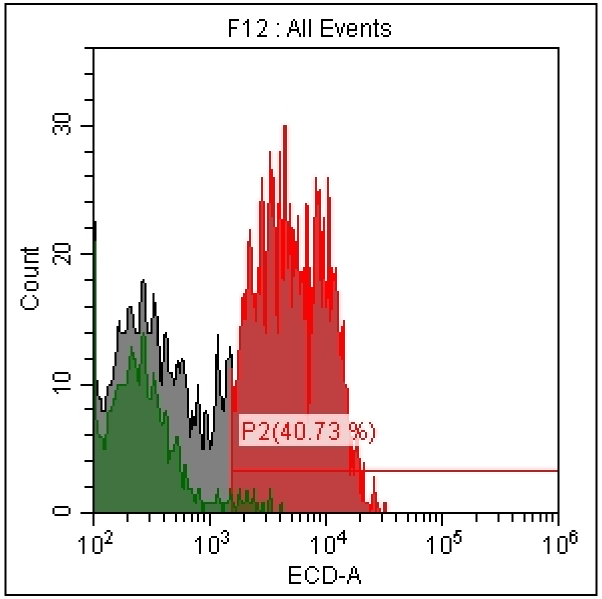

Experiment Name: KZ.20190422

Tube Name: F12

Sample ID:

Volume(μL): 187.8

Population	Mean FITC-A	Events	% Parent	Events/μL(V)	Median FITC-A	rCV FITC-A	...
All Events	18052.0	3000	100.00 %	15.97	5368.9	152.45 %	...
P2	39597.4	1222	40.73 %	6.51	28196.2	68.45 %	...
P1	4571.8	1187	39.57 %	6.32	744.0	119.58 %	...
