# Supplementary material for: An anionic human protein mediates cationic liposome delivery of genome editing proteins into mammalian cells
Source: Nat Commun. 2019 Jul 2;10:2905. doi: 10.1038/s41467-019-10828-3 (PMC6606574; doi:10.1038/s41467-019-10828-3)
Supplement: Supplementary file 3 — Source data [file 41467_2019_10828_MOESM3_ESM.zip › Supplementary Figures 5 and 6/F13.pdf]

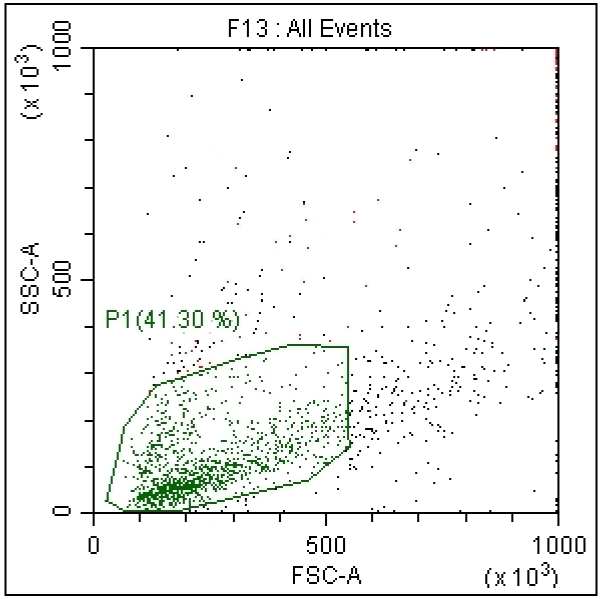

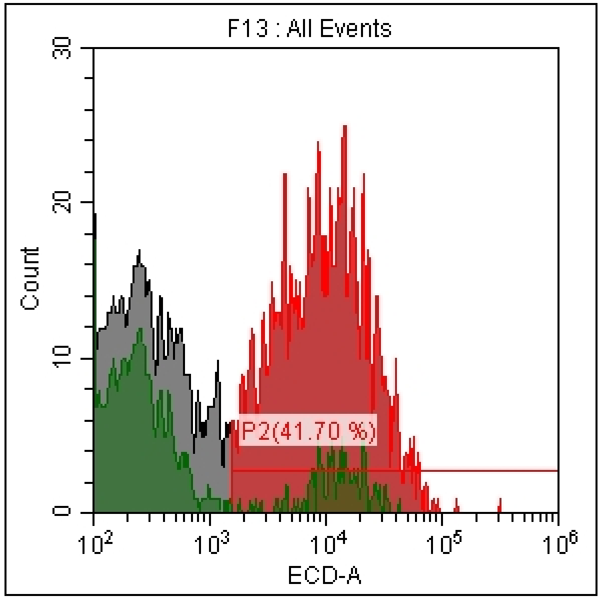

Experiment Name: KZ.20190422

Tube Name: F13

Sample ID:

Volume(μL): 209.1

Population	Mean FITC-A	Events	% Parent	Events/μL(V)	Median FITC-A	rCV FITC-A	...
All Events	12888.2	3000	100.00 %	14.35	2375.4	166.73 %	...
P2	28033.4	1251	41.70 %	5.98	21564.5	83.03 %	...
P1	746.3	1239	41.30 %	5.93	572.9	152.76 %	...
