# Supplementary material for: An anionic human protein mediates cationic liposome delivery of genome editing proteins into mammalian cells
Source: Nat Commun. 2019 Jul 2;10:2905. doi: 10.1038/s41467-019-10828-3 (PMC6606574; doi:10.1038/s41467-019-10828-3)
Supplement: Supplementary file 3 — Source data [file 41467_2019_10828_MOESM3_ESM.zip › Supplementary Figures 5 and 6/F14.pdf]

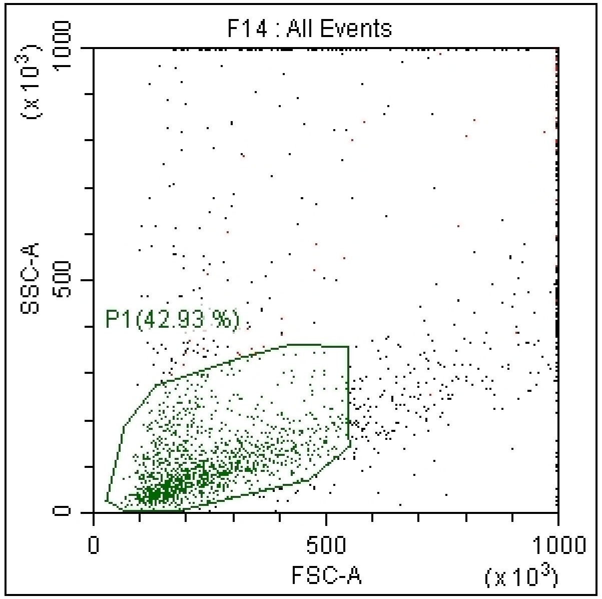

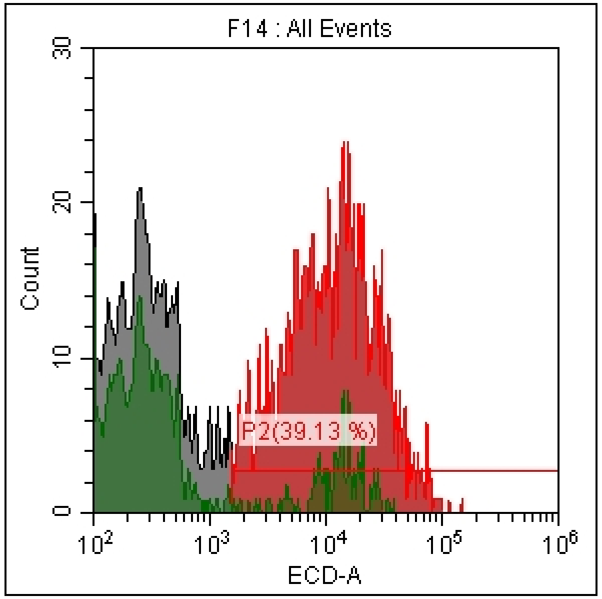

Experiment Name: KZ.20190422

Tube Name: F14

Sample ID:

Volume(μL): 205.6

Population	Mean FITC-A	Events	% Parent	Events/μL(V)	Median FITC-A	rCV FITC-A	...
All Events	13785.8	3000	100.00 %	14.59	1898.6	159.05 %	...
P2	32753.6	1174	39.13 %	5.71	20701.9	84.48 %	...
P1	670.0	1288	42.93 %	6.27	559.4	150.29 %	...
