# Supplementary material for: An anionic human protein mediates cationic liposome delivery of genome editing proteins into mammalian cells
Source: Nat Commun. 2019 Jul 2;10:2905. doi: 10.1038/s41467-019-10828-3 (PMC6606574; doi:10.1038/s41467-019-10828-3)
Supplement: Supplementary file 3 — Source data [file 41467_2019_10828_MOESM3_ESM.zip › Supplementary Figures 5 and 6/F15.pdf]

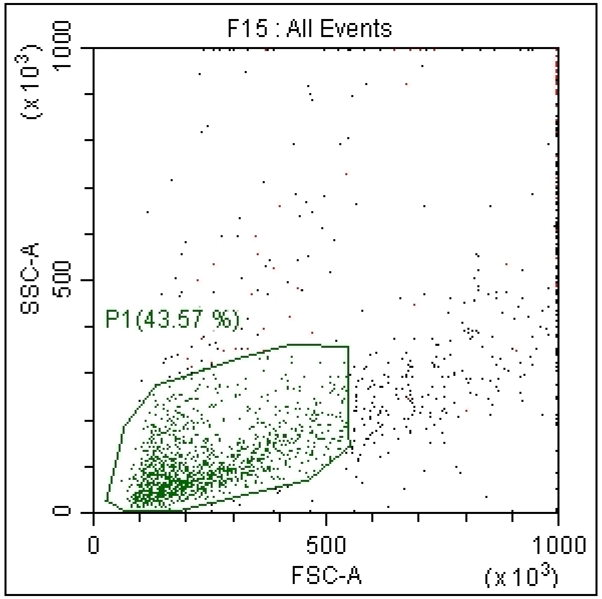

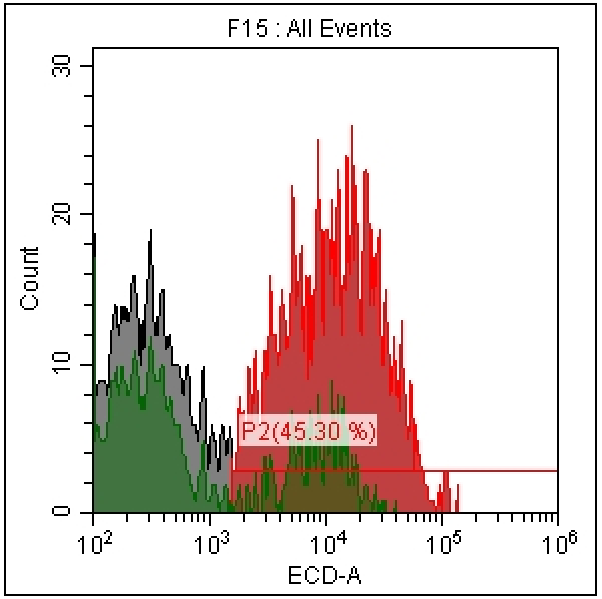

Experiment Name: KZ.20190422

Tube Name: F15

Sample ID:

Volume(μL): 210.4

Population	Mean FITC-A	Events	% Parent	Events/μL(V)	Median FITC-A	rCV FITC-A	...
All Events	12638.5	3000	100.00 %	14.26	2129.0	153.90 %	...
P2	26289.9	1359	45.30 %	6.46	18067.1	109.45 %	...
P1	800.6	1307	43.57 %	6.21	634.0	133.27 %	...
