# Supplementary material for: An anionic human protein mediates cationic liposome delivery of genome editing proteins into mammalian cells
Source: Nat Commun. 2019 Jul 2;10:2905. doi: 10.1038/s41467-019-10828-3 (PMC6606574; doi:10.1038/s41467-019-10828-3)
Supplement: Supplementary file 3 — Source data [file 41467_2019_10828_MOESM3_ESM.zip › Supplementary Figures 5 and 6/F16.pdf]

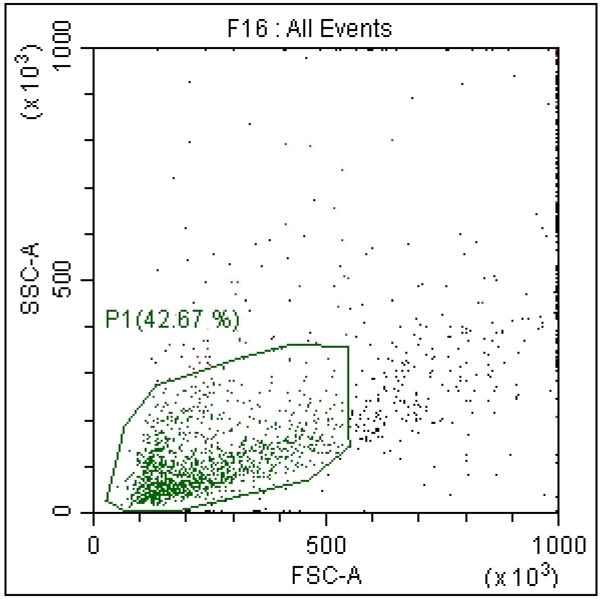

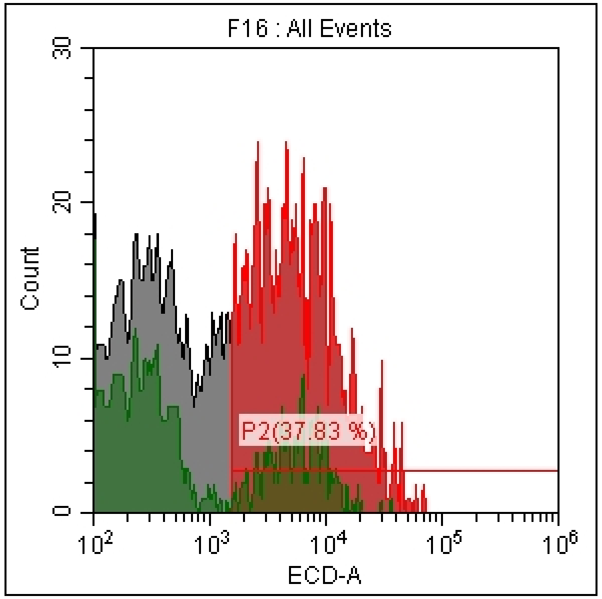

Experiment Name: KZ.20190422

Tube Name: F16

Sample ID:

Volume(μL): 215.4

Population	Mean FITC-A	Events	% Parent	Events/μL(V)	Median FITC-A	rCV FITC-A	...
All Events	13397.1	3000	100.00 %	13.93	2437.0	158.67 %	...
P2	29681.0	1135	37.83 %	5.27	23381.6	122.97 %	...
P1	761.1	1280	42.67 %	5.94	609.2	130.74 %	...
