# Supplementary material for: An anionic human protein mediates cationic liposome delivery of genome editing proteins into mammalian cells
Source: Nat Commun. 2019 Jul 2;10:2905. doi: 10.1038/s41467-019-10828-3 (PMC6606574; doi:10.1038/s41467-019-10828-3)
Supplement: Supplementary file 3 — Source data [file 41467_2019_10828_MOESM3_ESM.zip › Supplementary Figures 5 and 6/F17.pdf]

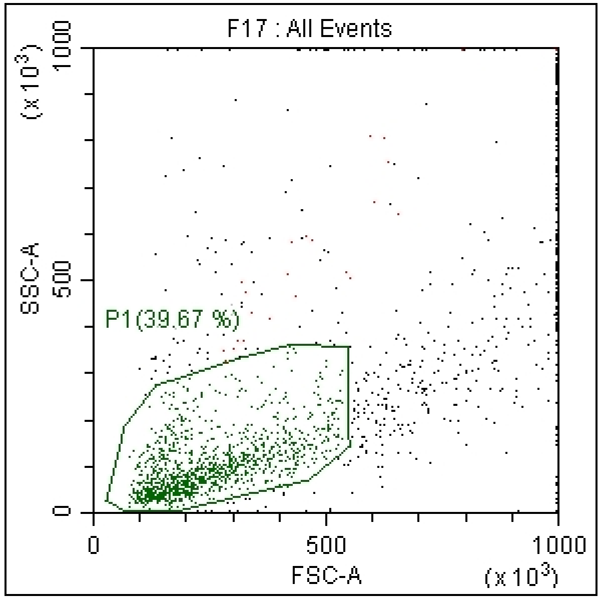

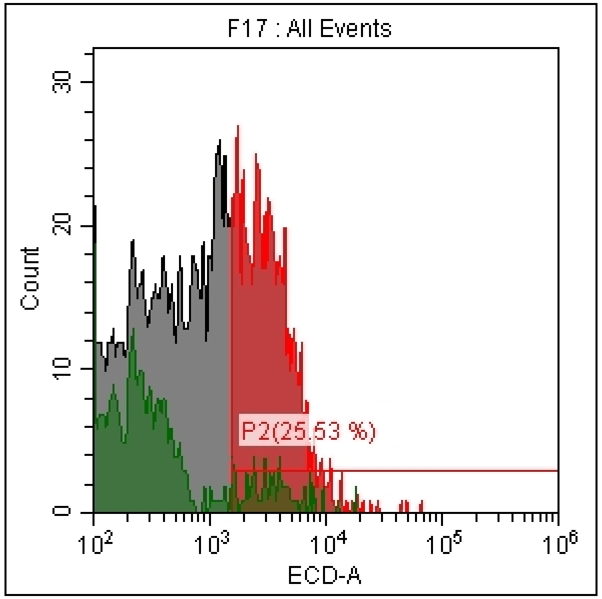

Experiment Name: KZ.20190422

Tube Name: F17

Sample ID:

Volume(µL): 194.5

Population	Mean FITC-A	Events	% Parent	Events/µL(V)	Median FITC-A	rCV FITC-A	...
All Events	13214.8	3000	100.00 %	15.42	2643.3	161.40 %	...
P2	37509.4	766	25.53 %	3.94	36602.6	64.80 %	...
P1	714.9	1190	39.67 %	6.12	566.3	153.95 %	...
