# Supplementary material for: An anionic human protein mediates cationic liposome delivery of genome editing proteins into mammalian cells
Source: Nat Commun. 2019 Jul 2;10:2905. doi: 10.1038/s41467-019-10828-3 (PMC6606574; doi:10.1038/s41467-019-10828-3)
Supplement: Supplementary file 3 — Source data [file 41467_2019_10828_MOESM3_ESM.zip › Supplementary Figures 5 and 6/F18.pdf]

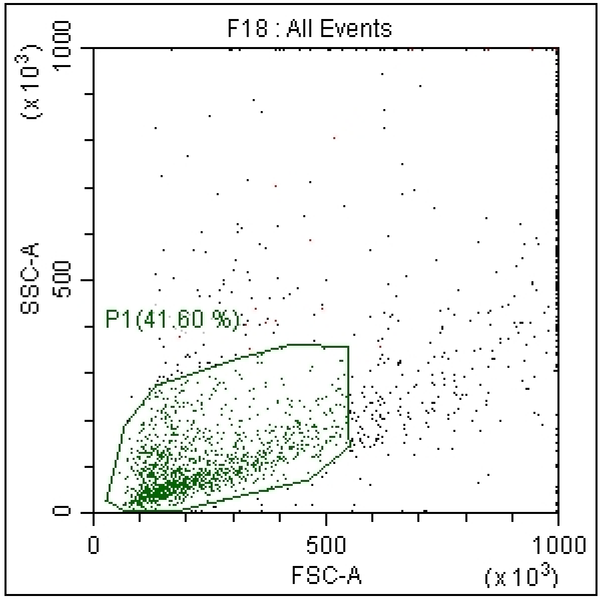

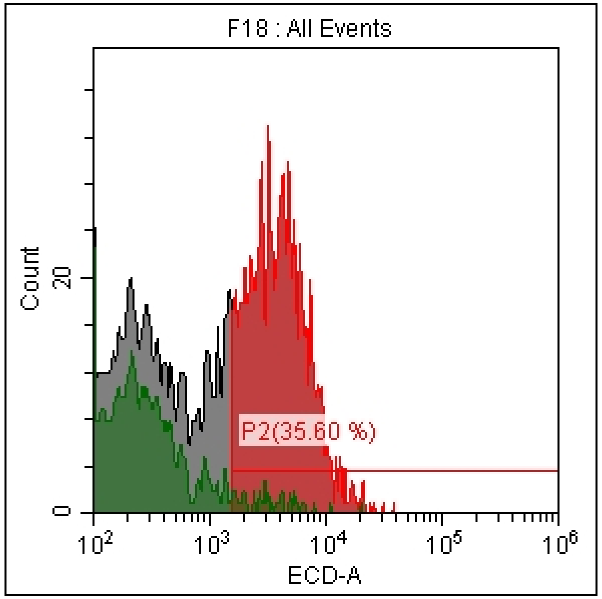

Experiment Name: KZ.20190422

Tube Name: F18

Sample ID:

Volume(μL): 207.8

Population	Mean FITC-A	Events	% Parent	Events/μL(V)	Median FITC-A	rCV FITC-A	...
All Events	12788.9	3000	100.00 %	14.44	2562.8	158.53 %	...
P2	31657.0	1068	35.60 %	5.14	24711.3	66.85 %	...
P1	844.9	1248	41.60 %	6.01	613.5	134.58 %	...
