# Supplementary material for: An anionic human protein mediates cationic liposome delivery of genome editing proteins into mammalian cells
Source: Nat Commun. 2019 Jul 2;10:2905. doi: 10.1038/s41467-019-10828-3 (PMC6606574; doi:10.1038/s41467-019-10828-3)
Supplement: Supplementary file 3 — Source data [file 41467_2019_10828_MOESM3_ESM.zip › Supplementary Figures 5 and 6/F2.pdf]

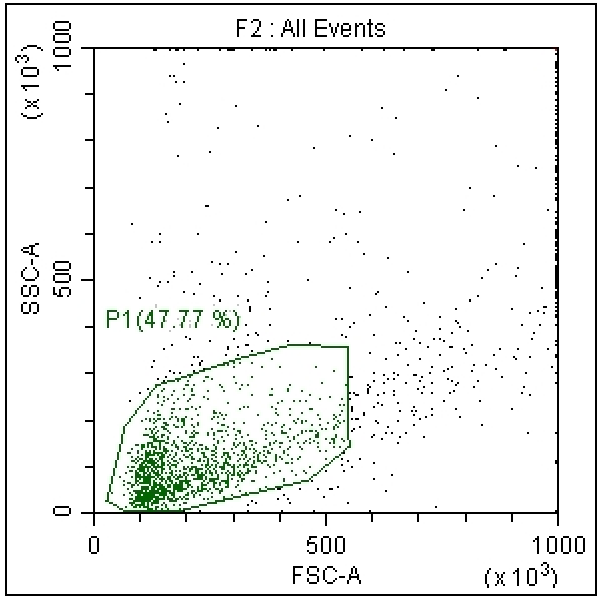

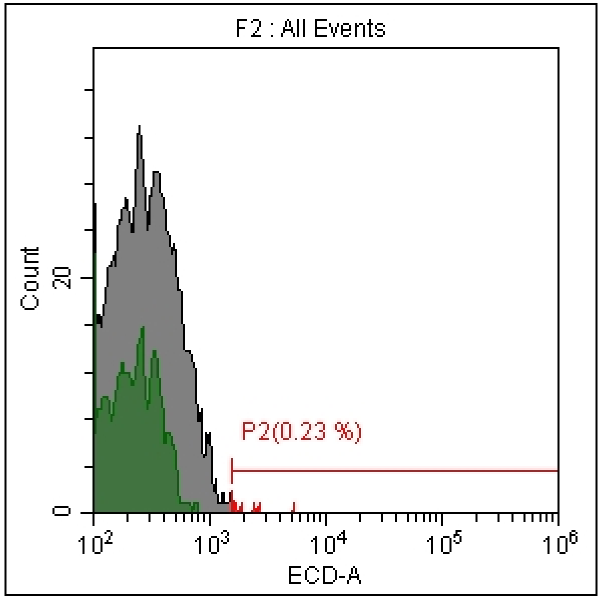

Experiment Name: KZ.20190422
Tube Name: F2
Sample ID:
Volume(μL): 164.9

Population	Mean FITC-A	Events	% Parent	Events/μL(V)	Median FITC-A	rCV FITC-A	...
All Events	11434.9	3000	100.00 %	18.19	1755.0	147.51 %	...
P2	85464.5	7	0.23 %	0.04	16218.3	671.45 %	...
P1	769.5	1433	47.77 %	8.69	625.8	129.59 %	...
