# Supplementary material for: An anionic human protein mediates cationic liposome delivery of genome editing proteins into mammalian cells
Source: Nat Commun. 2019 Jul 2;10:2905. doi: 10.1038/s41467-019-10828-3 (PMC6606574; doi:10.1038/s41467-019-10828-3)
Supplement: Supplementary file 3 — Source data [file 41467_2019_10828_MOESM3_ESM.zip › Supplementary Figures 5 and 6/F3.pdf]

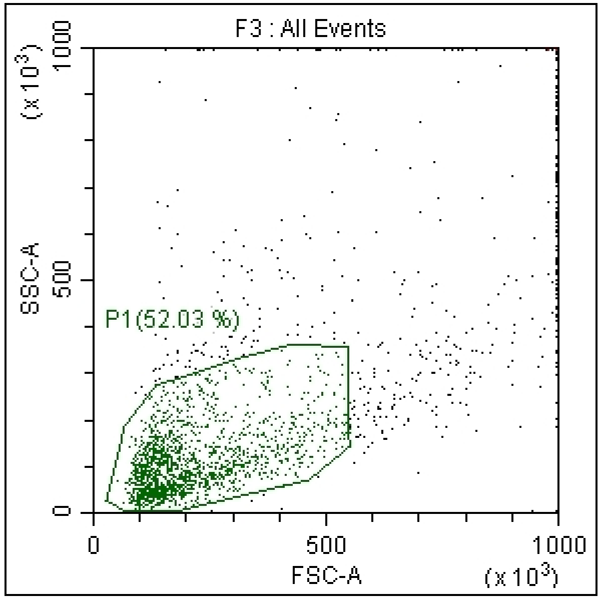

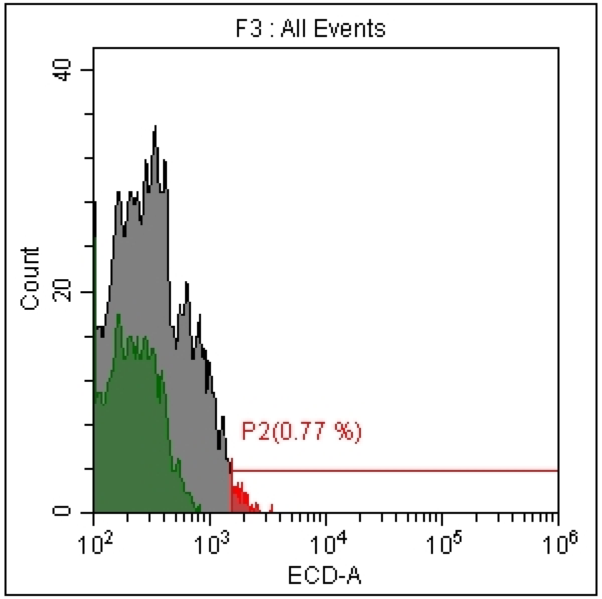

Experiment Name: KZ.20190422

Tube Name: F3

Sample ID:

Volume(μL): 184.1

Population	Mean FITC-A	Events	% Parent	Events/μL(V)	Median FITC-A	rCV FITC-A	...
All Events	11406.2	3000	100.00 %	16.29	1743.9	143.55 %	...
P2	87249.6	23	0.77 %	0.12	89766.9	24.30 %	...
P1	903.0	1561	52.03 %	8.48	807.3	112.08 %	...
