# Supplementary material for: An anionic human protein mediates cationic liposome delivery of genome editing proteins into mammalian cells
Source: Nat Commun. 2019 Jul 2;10:2905. doi: 10.1038/s41467-019-10828-3 (PMC6606574; doi:10.1038/s41467-019-10828-3)
Supplement: Supplementary file 3 — Source data [file 41467_2019_10828_MOESM3_ESM.zip › Supplementary Figures 5 and 6/F4.pdf]

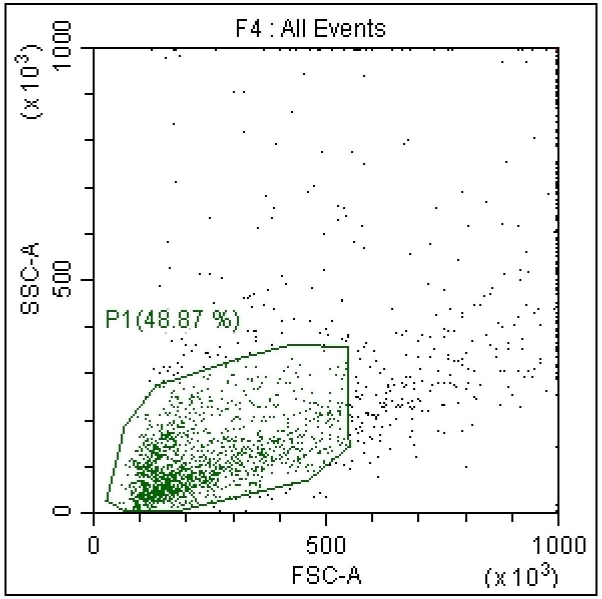

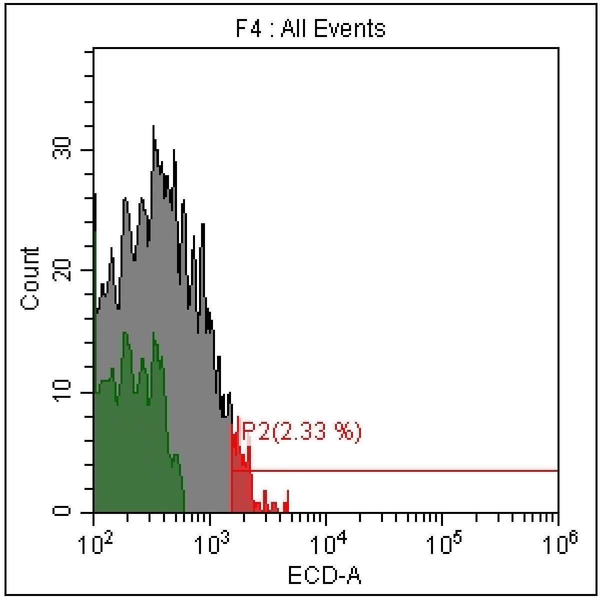

Experiment Name: KZ.20190422

Tube Name: F4

Sample ID:

Volume(μL): 188.7

Population	Mean FITC-A	Events	% Parent	Events/μL(V)	Median FITC-A	rCV FITC-A	...
All Events	11359.9	3000	100.00 %	15.90	1847.4	146.63 %	...
P2	76933.3	70	2.33 %	0.37	71281.9	32.84 %	...
P1	802.5	1466	48.87 %	7.77	664.0	126.86 %	...
