# Supplementary material for: An anionic human protein mediates cationic liposome delivery of genome editing proteins into mammalian cells
Source: Nat Commun. 2019 Jul 2;10:2905. doi: 10.1038/s41467-019-10828-3 (PMC6606574; doi:10.1038/s41467-019-10828-3)
Supplement: Supplementary file 3 — Source data [file 41467_2019_10828_MOESM3_ESM.zip › Supplementary Figures 5 and 6/F5.pdf]

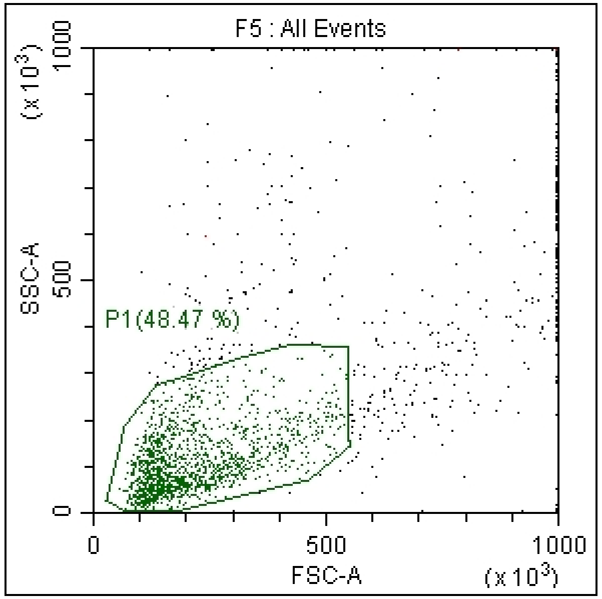

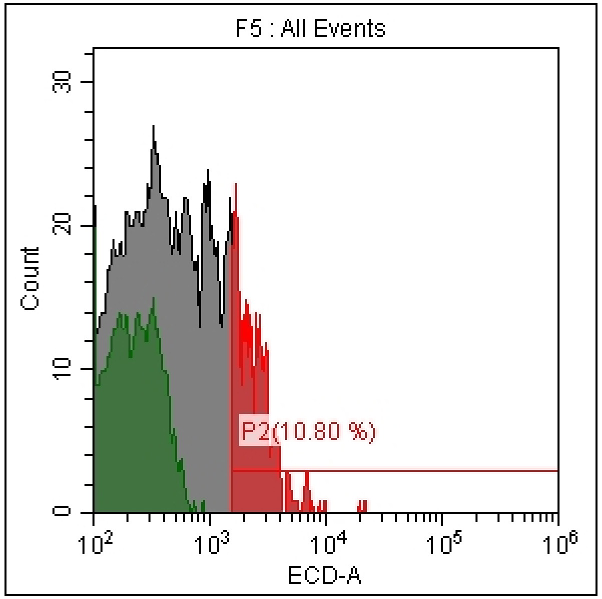

Experiment Name: KZ.20190422
Tube Name: F5
Sample ID:
Volume(μL): 185.5

Population	Mean FITC-A	Events	% Parent	Events/μL(V)	Median FITC-A	rCV FITC-A	...
All Events	12520.7	3000	100.00 %	16.17	2026.9	148.63 %	...
P2	62158.9	324	10.80 %	1.75	49600.1	47.14 %	...
P1	821.7	1454	48.47 %	7.84	680.7	126.02 %	...
