# Supplementary material for: An anionic human protein mediates cationic liposome delivery of genome editing proteins into mammalian cells
Source: Nat Commun. 2019 Jul 2;10:2905. doi: 10.1038/s41467-019-10828-3 (PMC6606574; doi:10.1038/s41467-019-10828-3)
Supplement: Supplementary file 3 — Source data [file 41467_2019_10828_MOESM3_ESM.zip › Supplementary Figures 5 and 6/F6.pdf]

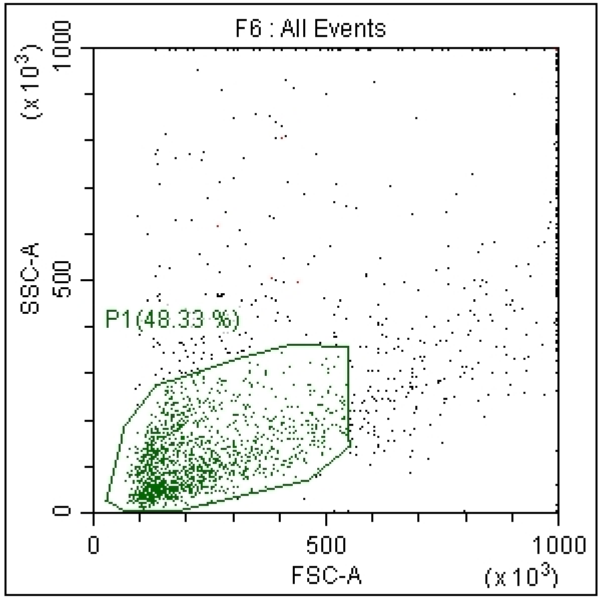

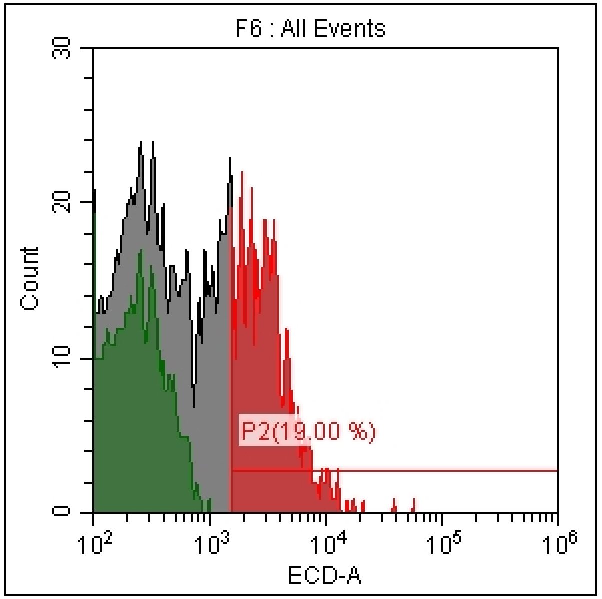

Experiment Name: KZ.20190422

Tube Name: F6

Sample ID:

Volume(µL): 210.2

Population	Mean FITC-A	Events	% Parent	Events/µL(V)	Median FITC-A	rCV FITC-A	...
All Events	13574.7	3000	100.00 %	14.27	1843.0	145.25 %	...
P2	56470.6	570	19.00 %	2.71	39241.4	61.14 %	...
P1	859.1	1450	48.33 %	6.90	713.8	125.62 %	...
