# Supplementary material for: An anionic human protein mediates cationic liposome delivery of genome editing proteins into mammalian cells
Source: Nat Commun. 2019 Jul 2;10:2905. doi: 10.1038/s41467-019-10828-3 (PMC6606574; doi:10.1038/s41467-019-10828-3)
Supplement: Supplementary file 3 — Source data [file 41467_2019_10828_MOESM3_ESM.zip › Supplementary Figures 5 and 6/F7.pdf]

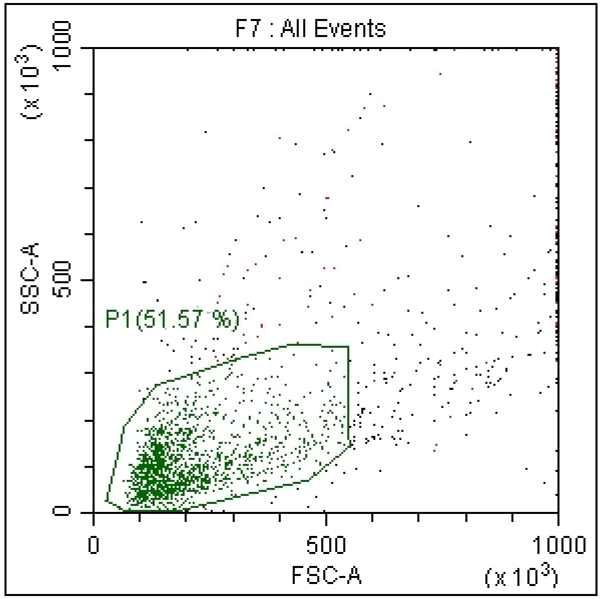

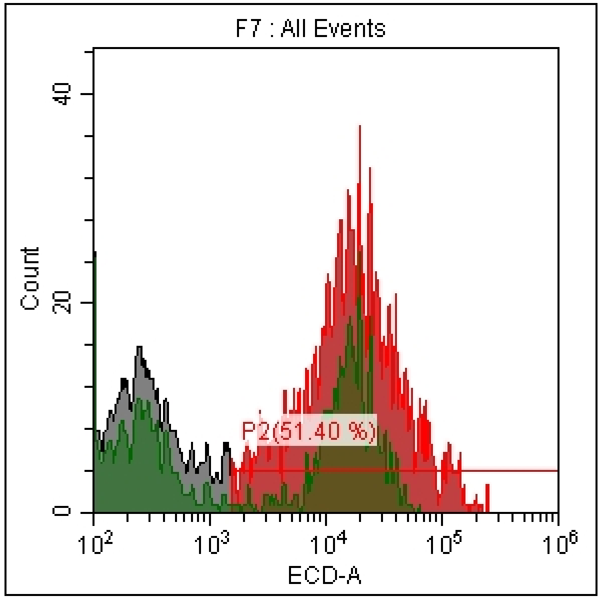

Experiment Name: KZ.20190422

Tube Name: F7

Sample ID:

Volume(μL): 166.2

Population	Mean FITC-A	Events	% Parent	Events/μL(V)	Median FITC-A	rCV FITC-A	...
All Events	573527.6	3000	100.00 %	18.05	43050.0	150.40 %	...
P2	1110311.3	1542	51.40 %	9.28	640310.4	89.09 %	...
P1	236452.6	1547	51.57 %	9.31	1983.8	181.03 %	...
