# Supplementary material for: An anionic human protein mediates cationic liposome delivery of genome editing proteins into mammalian cells
Source: Nat Commun. 2019 Jul 2;10:2905. doi: 10.1038/s41467-019-10828-3 (PMC6606574; doi:10.1038/s41467-019-10828-3)
Supplement: Supplementary file 3 — Source data [file 41467_2019_10828_MOESM3_ESM.zip › Supplementary Figures 5 and 6/F8.pdf]

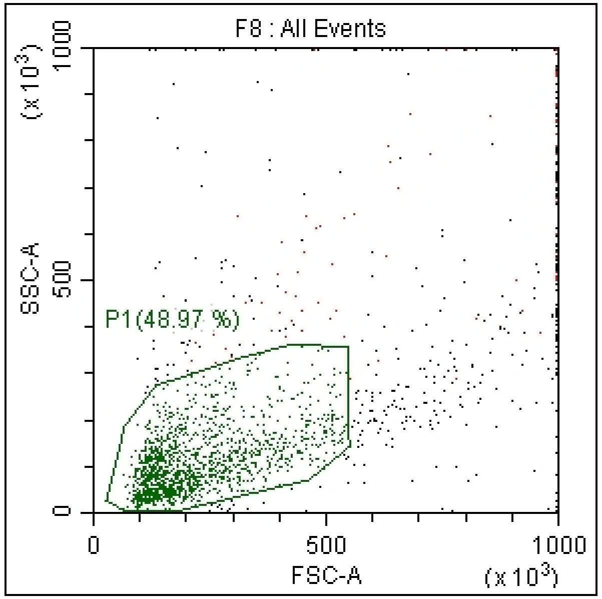

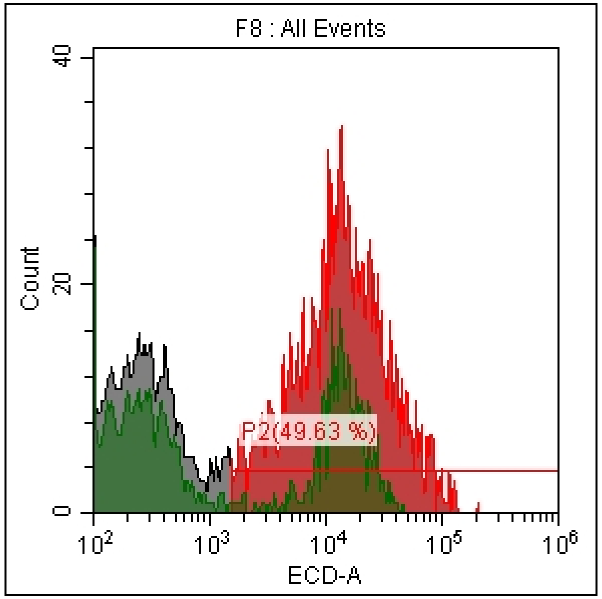

Experiment Name: KZ.20190422

Tube Name: F8

Sample ID:

Volume(μL): 182.1

Population	Mean FITC-A	Events	% Parent	Events/μL(V)	Median FITC-A	rCV FITC-A	...
All Events	309522.9	3000	100.00 %	16.47	22499.9	149.94 %	...
P2	619380.1	1489	49.63 %	8.18	382480.6	112.18 %	...
P1	154844.7	1469	48.97 %	8.07	1292.6	165.61 %	...
