# Supplementary material for: An anionic human protein mediates cationic liposome delivery of genome editing proteins into mammalian cells
Source: Nat Commun. 2019 Jul 2;10:2905. doi: 10.1038/s41467-019-10828-3 (PMC6606574; doi:10.1038/s41467-019-10828-3)
Supplement: Supplementary file 3 — Source data [file 41467_2019_10828_MOESM3_ESM.zip › Supplementary Figures 5 and 6/F9.pdf]

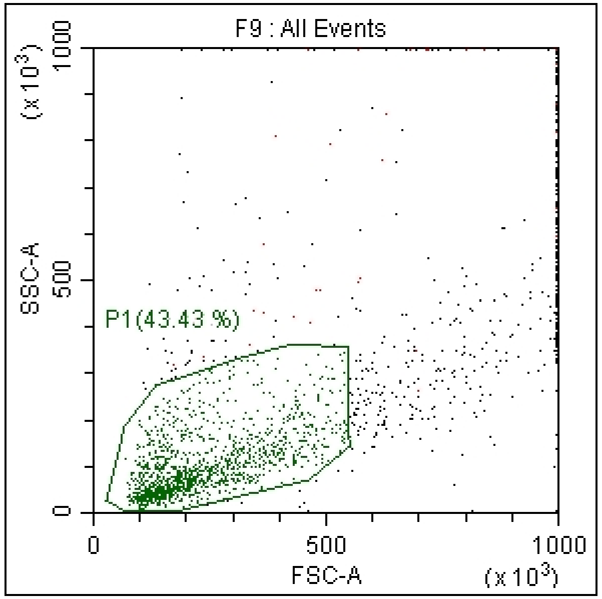

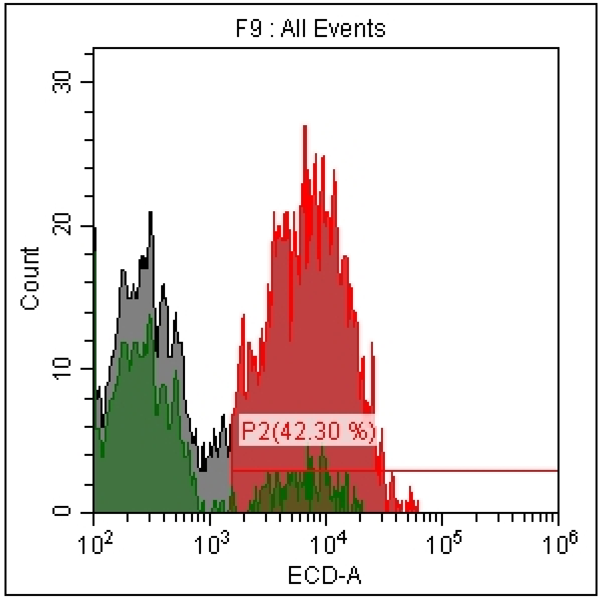

Experiment Name: KZ.20190422

Tube Name: F9

Sample ID:

Volume(μL): 182.1

Population	Mean FITC-A	Events	% Parent	Events/μL(V)	Median FITC-A	rCV FITC-A	...
All Events	57483.9	3000	100.00 %	16.48	6286.3	154.57 %	...
P2	131749.5	1269	42.30 %	6.97	75840.7	103.35 %	...
P1	27018.6	1303	43.43 %	7.16	794.9	139.75 %	...
