# Supplementary material for: An anionic human protein mediates cationic liposome delivery of genome editing proteins into mammalian cells
Source: Nat Commun. 2019 Jul 2;10:2905. doi: 10.1038/s41467-019-10828-3 (PMC6606574; doi:10.1038/s41467-019-10828-3)
Supplement: Supplementary file 3 — Source data [file 41467_2019_10828_MOESM3_ESM.zip › Supplementary Figures 5 and 6/H0.pdf]

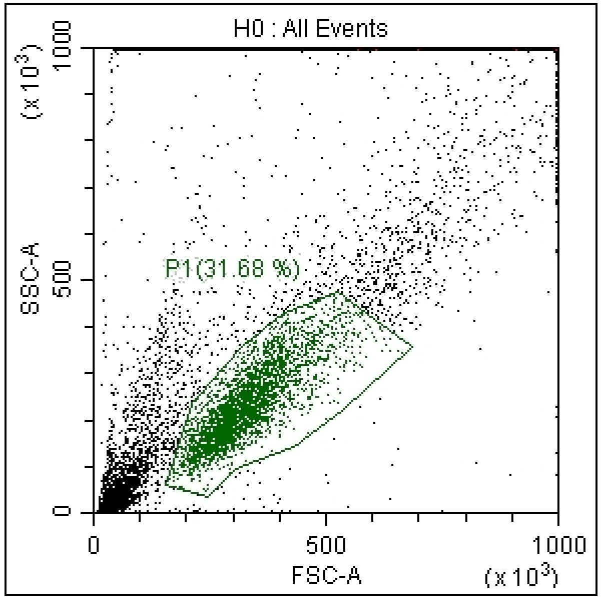

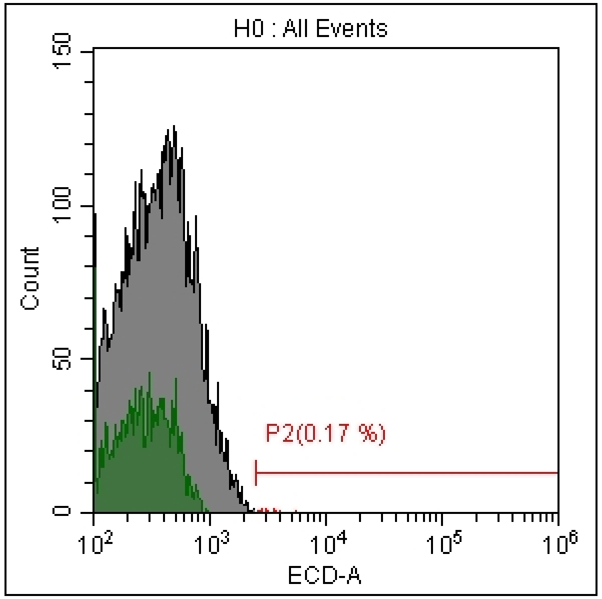

Experiment Name: KZ.20190422

Tube Name: H0

Sample ID:

Volume(μL): 95.5

Population	Mean FITC-A	Events	% Parent	Events/μL(V)	Median FITC-A	rCV FITC-A	...
All Events	20590.1	10000	100.00 %	104.68	11473.5	133.41 %	...
P2	38434.2	17	0.17 %	0.18	34951.7	67.30 %	...
P1	20296.3	3168	31.68 %	33.16	19384.6	50.38 %	...
