# Supplementary material for: An anionic human protein mediates cationic liposome delivery of genome editing proteins into mammalian cells
Source: Nat Commun. 2019 Jul 2;10:2905. doi: 10.1038/s41467-019-10828-3 (PMC6606574; doi:10.1038/s41467-019-10828-3)
Supplement: Supplementary file 3 — Source data [file 41467_2019_10828_MOESM3_ESM.zip › Supplementary Figures 5 and 6/H1.pdf]

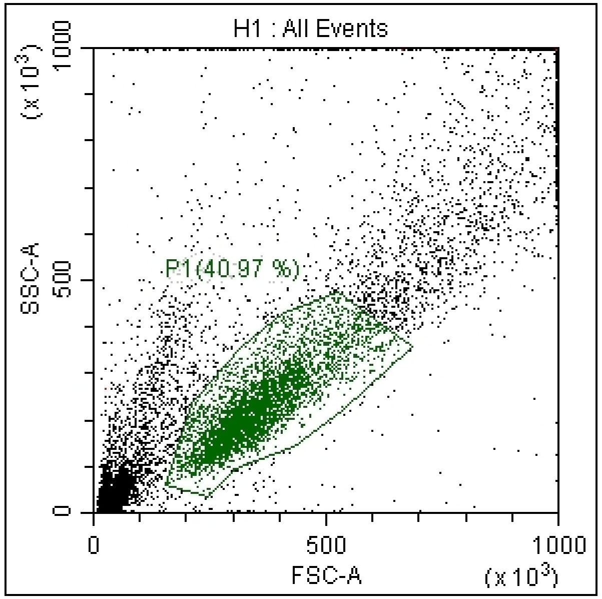

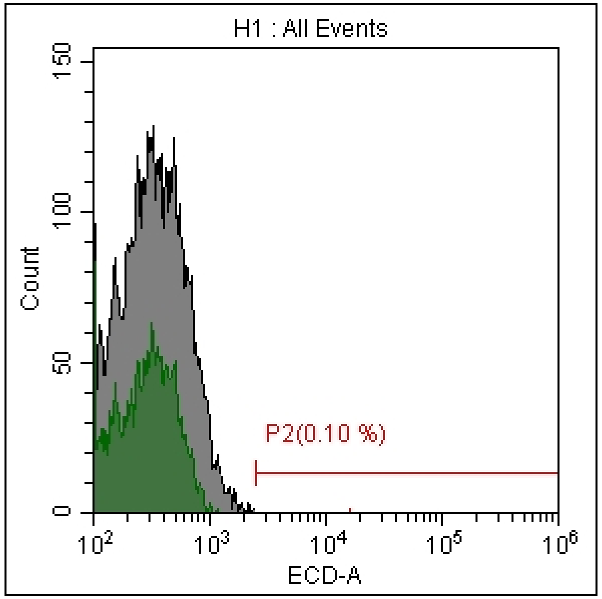

Experiment Name: KZ.20190422

Tube Name: H1

Sample ID:

Volume(μL): 223.6

Population	Mean FITC-A	Events	% Parent	Events/μL(V)	Median FITC-A	rCV FITC-A	...
All Events	38054.1	10000	100.00 %	44.71	21698.5	117.18 %	...
P2	1000758.0	10	0.10 %	0.04	838817.4	57.51 %	...
P1	24698.2	4097	40.97 %	18.32	21878.3	49.67 %	...
