# Supplementary material for: An anionic human protein mediates cationic liposome delivery of genome editing proteins into mammalian cells
Source: Nat Commun. 2019 Jul 2;10:2905. doi: 10.1038/s41467-019-10828-3 (PMC6606574; doi:10.1038/s41467-019-10828-3)
Supplement: Supplementary file 3 — Source data [file 41467_2019_10828_MOESM3_ESM.zip › Supplementary Figures 5 and 6/H10.pdf]

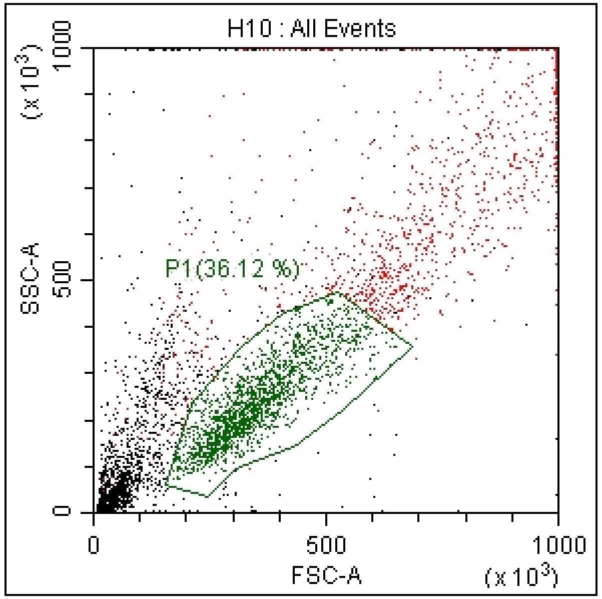

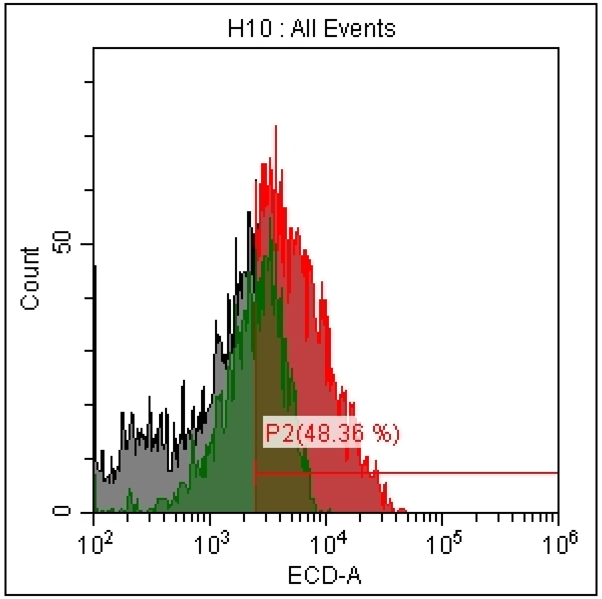

Experiment Name: KZ.20190422

Tube Name: H10

Sample ID:

Volume(µL): 106.8

Population	Mean FITC-A	Events	% Parent	Events/µL(V)	Median FITC-A	rCV FITC-A	...
All Events	51221.1	5000	100.00 %	46.80	27121.9	124.35 %	...
P2	92649.6	2418	48.36 %	22.63	67172.8	79.01 %	...
P1	29245.3	1806	36.12 %	16.90	24880.4	52.74 %	...
