# Supplementary material for: An anionic human protein mediates cationic liposome delivery of genome editing proteins into mammalian cells
Source: Nat Commun. 2019 Jul 2;10:2905. doi: 10.1038/s41467-019-10828-3 (PMC6606574; doi:10.1038/s41467-019-10828-3)
Supplement: Supplementary file 3 — Source data [file 41467_2019_10828_MOESM3_ESM.zip › Supplementary Figures 5 and 6/H11.pdf]

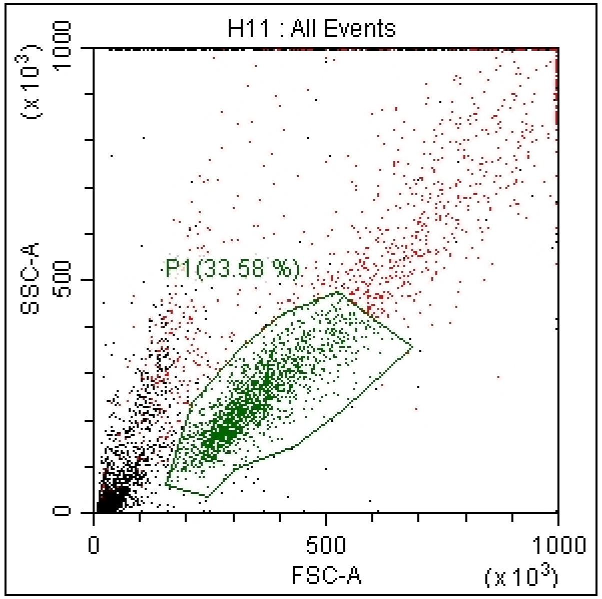

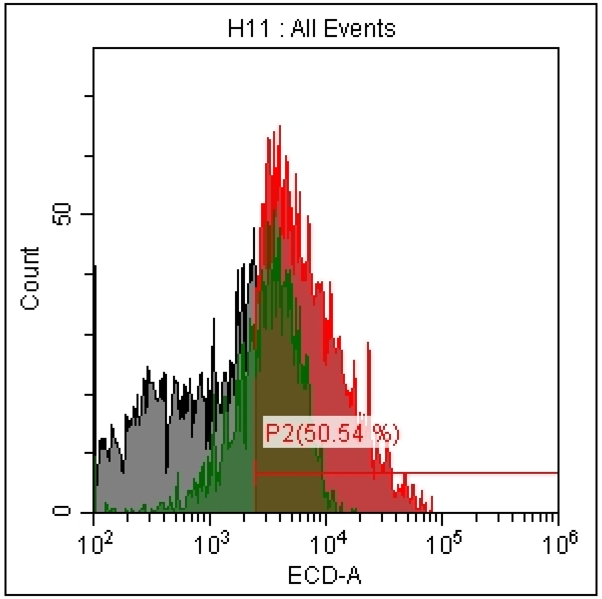

Experiment Name: KZ.20190422

Tube Name: H11

Sample ID:

Volume(μL): 101.4

Population	Mean FITC-A	Events	% Parent	Events/μL(V)	Median FITC-A	rCV FITC-A	...
All Events	49297.1	5000	100.00 %	49.30	22437.4	125.63 %	...
P2	88110.1	2527	50.54 %	24.92	51177.7	82.90 %	...
P1	28095.6	1679	33.58 %	16.56	23149.7	51.92 %	...
