# Supplementary material for: An anionic human protein mediates cationic liposome delivery of genome editing proteins into mammalian cells
Source: Nat Commun. 2019 Jul 2;10:2905. doi: 10.1038/s41467-019-10828-3 (PMC6606574; doi:10.1038/s41467-019-10828-3)
Supplement: Supplementary file 3 — Source data [file 41467_2019_10828_MOESM3_ESM.zip › Supplementary Figures 5 and 6/H12.pdf]

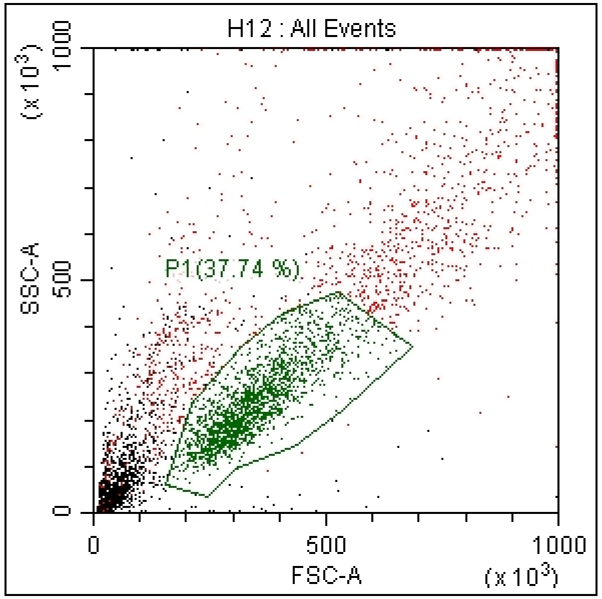

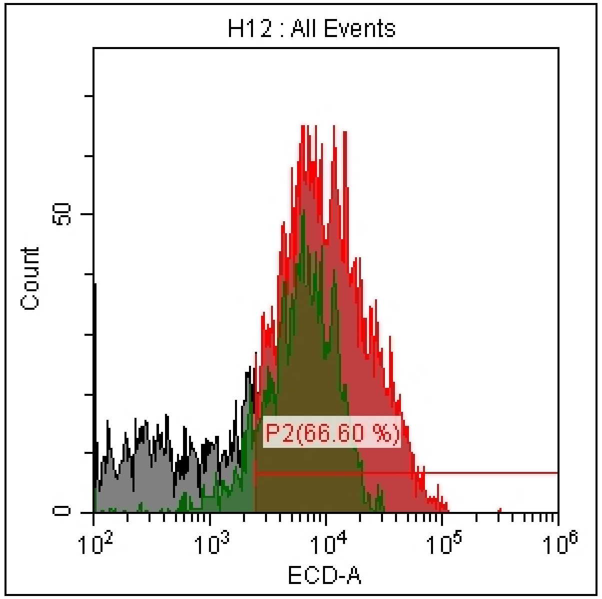

Experiment Name: KZ.20190422

Tube Name: H12

Sample ID:

Volume(μL): 122.5

Population	Mean FITC-A	Events	% Parent	Events/μL(V)	Median FITC-A	rCV FITC-A	...
All Events	56712.5	5000	100.00 %	40.80	27674.5	119.96 %	...
P2	81492.5	3330	66.60 %	27.17	45639.3	83.33 %	...
P1	29352.6	1887	37.74 %	15.40	24913.7	52.22 %	...
