# Supplementary material for: An anionic human protein mediates cationic liposome delivery of genome editing proteins into mammalian cells
Source: Nat Commun. 2019 Jul 2;10:2905. doi: 10.1038/s41467-019-10828-3 (PMC6606574; doi:10.1038/s41467-019-10828-3)
Supplement: Supplementary file 3 — Source data [file 41467_2019_10828_MOESM3_ESM.zip › Supplementary Figures 5 and 6/H13.pdf]

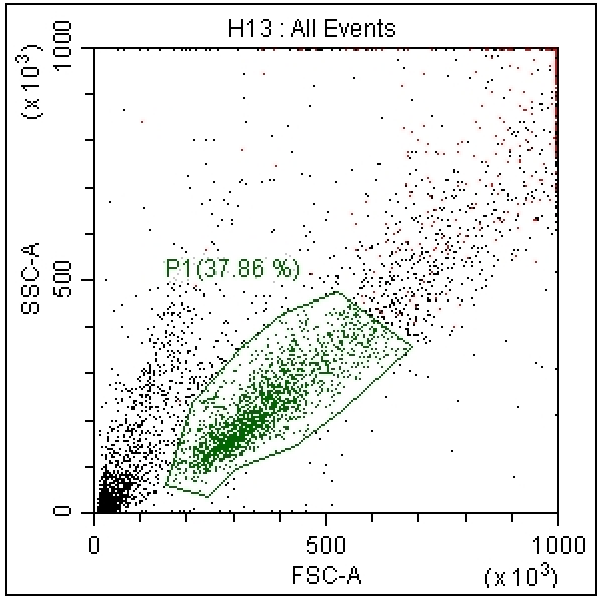

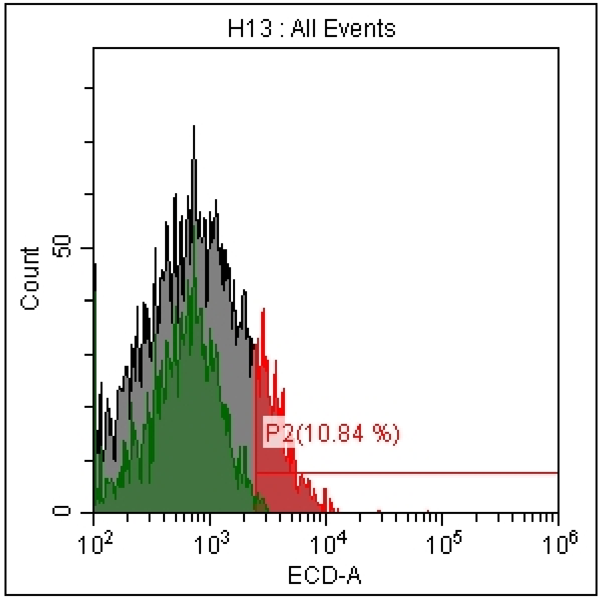

Experiment Name: KZ.20190422

Tube Name: H13

Sample ID:

Volume(µL): 89.1

Population	Mean FITC-A	Events	% Parent	Events/µL(V)	Median FITC-A	rCV FITC-A	...
All Events	47091.6	5000	100.00 %	56.11	25924.6	123.20 %	...
P2	167061.7	542	10.84 %	6.08	138019.8	56.36 %	...
P1	26689.9	1893	37.86 %	21.24	23174.9	53.90 %	...
