# Supplementary material for: An anionic human protein mediates cationic liposome delivery of genome editing proteins into mammalian cells
Source: Nat Commun. 2019 Jul 2;10:2905. doi: 10.1038/s41467-019-10828-3 (PMC6606574; doi:10.1038/s41467-019-10828-3)
Supplement: Supplementary file 3 — Source data [file 41467_2019_10828_MOESM3_ESM.zip › Supplementary Figures 5 and 6/H14.pdf]

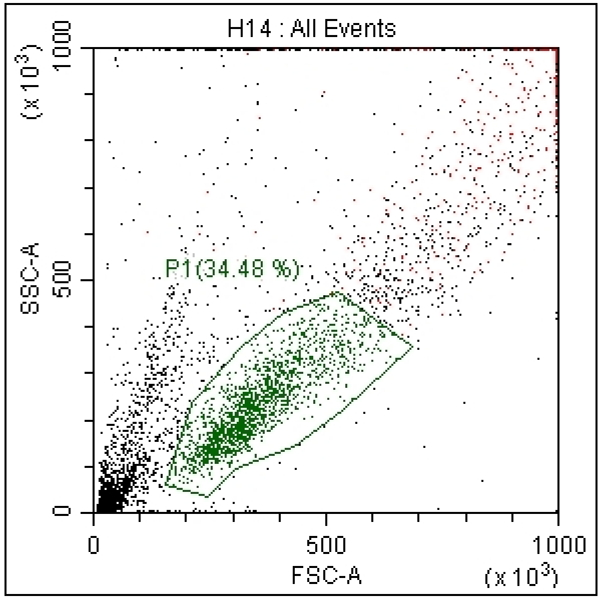

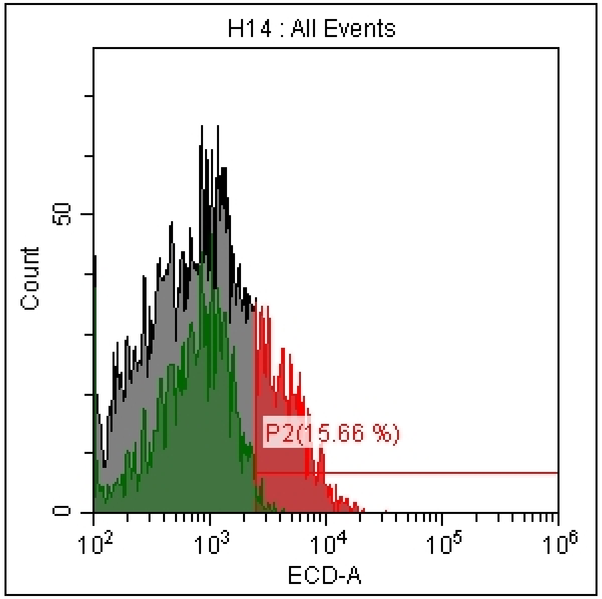

Experiment Name: KZ.20190422

Tube Name: H14

Sample ID:

Volume(µL): 120.6

Population	Mean FITC-A	Events	% Parent	Events/µL(V)	Median FITC-A	rCV FITC-A	...
All Events	45018.5	5000	100.00 %	41.45	21800.1	127.68 %	...
P2	158858.9	783	15.66 %	6.49	131893.5	53.94 %	...
P1	24908.1	1724	34.48 %	14.29	21613.1	51.48 %	...
