# Supplementary material for: An anionic human protein mediates cationic liposome delivery of genome editing proteins into mammalian cells
Source: Nat Commun. 2019 Jul 2;10:2905. doi: 10.1038/s41467-019-10828-3 (PMC6606574; doi:10.1038/s41467-019-10828-3)
Supplement: Supplementary file 3 — Source data [file 41467_2019_10828_MOESM3_ESM.zip › Supplementary Figures 5 and 6/H15.pdf]

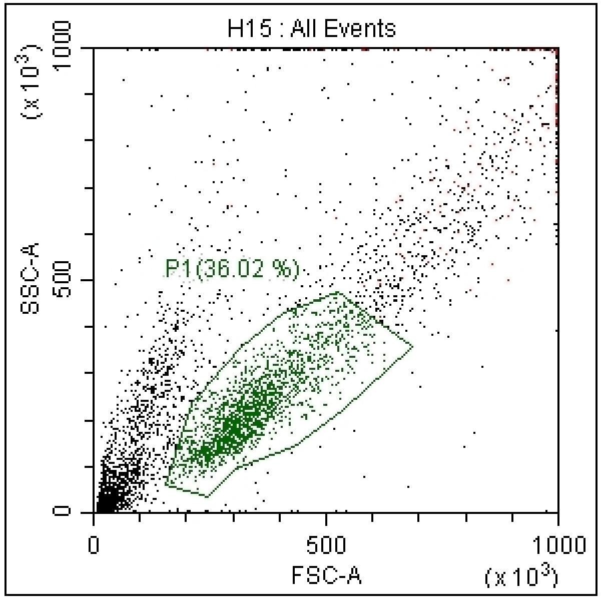

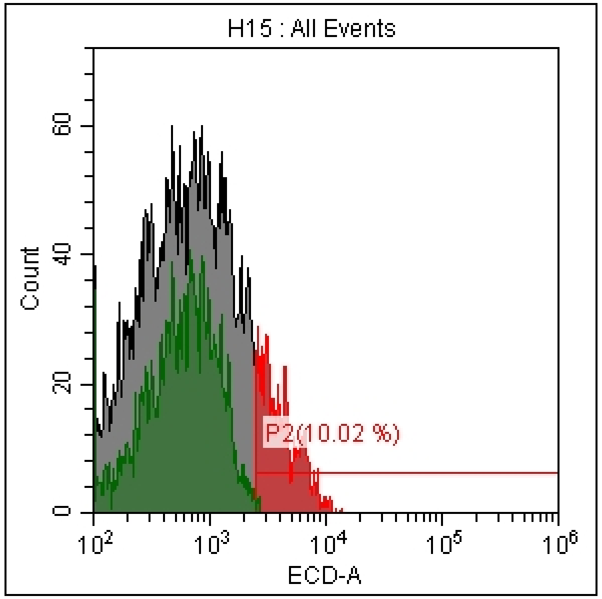

Experiment Name: KZ.20190422

Tube Name: H15

Sample ID:

Volume(μL): 112.9

Population	Mean FITC-A	Events	% Parent	Events/μL(V)	Median FITC-A	rCV FITC-A	...
All Events	45426.5	5000	100.00 %	44.27	21941.2	121.71 %	...
P2	200401.3	501	10.02 %	4.44	184132.0	50.75 %	...
P1	23938.3	1801	36.02 %	15.95	21183.8	48.17 %	...
