# Supplementary material for: An anionic human protein mediates cationic liposome delivery of genome editing proteins into mammalian cells
Source: Nat Commun. 2019 Jul 2;10:2905. doi: 10.1038/s41467-019-10828-3 (PMC6606574; doi:10.1038/s41467-019-10828-3)
Supplement: Supplementary file 3 — Source data [file 41467_2019_10828_MOESM3_ESM.zip › Supplementary Figures 5 and 6/H16.pdf]

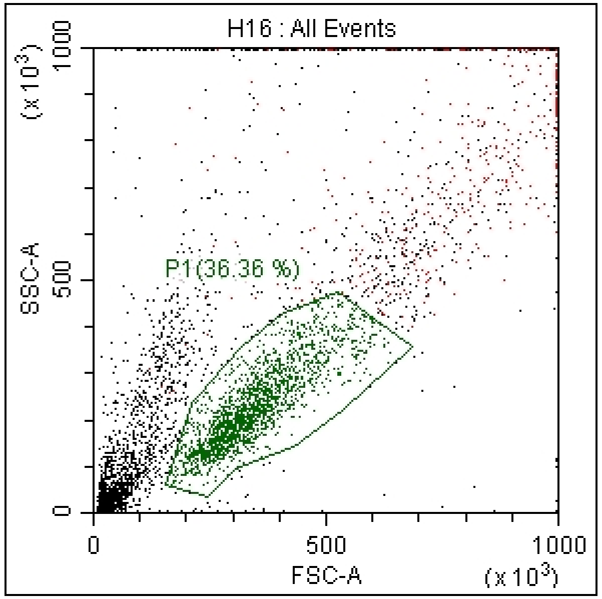

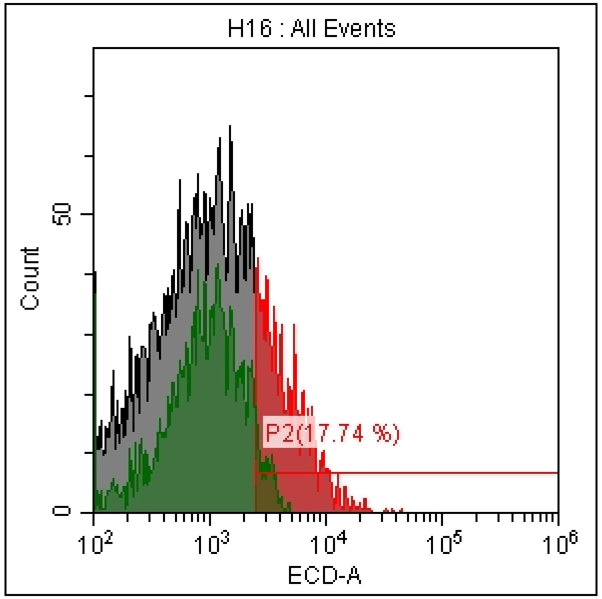

Experiment Name: KZ.20190422

Tube Name: H16

Sample ID:

Volume(μL): 102.4

Population	Mean FITC-A	Events	% Parent	Events/μL(V)	Median FITC-A	rCV FITC-A	...
All Events	37964.3	5000	100.00 %	48.84	20221.7	122.99 %	...
P2	126903.0	887	17.74 %	8.66	104189.1	65.28 %	...
P1	23542.3	1818	36.36 %	17.76	20705.7	50.67 %	...
