# Supplementary material for: An anionic human protein mediates cationic liposome delivery of genome editing proteins into mammalian cells
Source: Nat Commun. 2019 Jul 2;10:2905. doi: 10.1038/s41467-019-10828-3 (PMC6606574; doi:10.1038/s41467-019-10828-3)
Supplement: Supplementary file 3 — Source data [file 41467_2019_10828_MOESM3_ESM.zip › Supplementary Figures 5 and 6/H17.pdf]

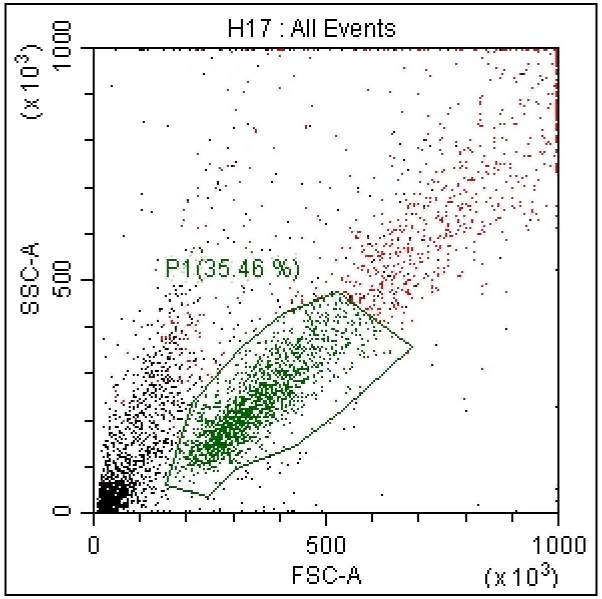

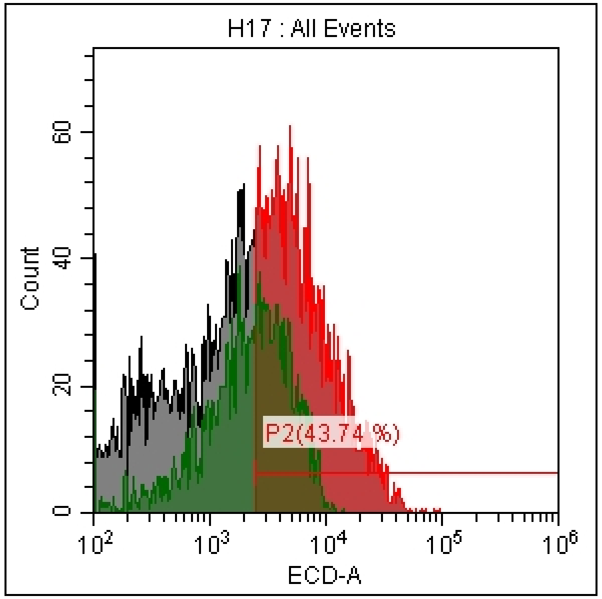

Experiment Name: KZ.20190422

Tube Name: H17

Sample ID:

Volume(µL): 107.8

Population	Mean FITC-A	Events	% Parent	Events/µL(V)	Median FITC-A	rCV FITC-A	...
All Events	44247.6	5000	100.00 %	46.37	22486.3	119.52 %	...
P2	83289.2	2187	43.74 %	20.28	55019.0	84.88 %	...
P1	23681.8	1773	35.46 %	16.44	21342.5	52.39 %	...
