# Supplementary material for: An anionic human protein mediates cationic liposome delivery of genome editing proteins into mammalian cells
Source: Nat Commun. 2019 Jul 2;10:2905. doi: 10.1038/s41467-019-10828-3 (PMC6606574; doi:10.1038/s41467-019-10828-3)
Supplement: Supplementary file 3 — Source data [file 41467_2019_10828_MOESM3_ESM.zip › Supplementary Figures 5 and 6/H18.pdf]

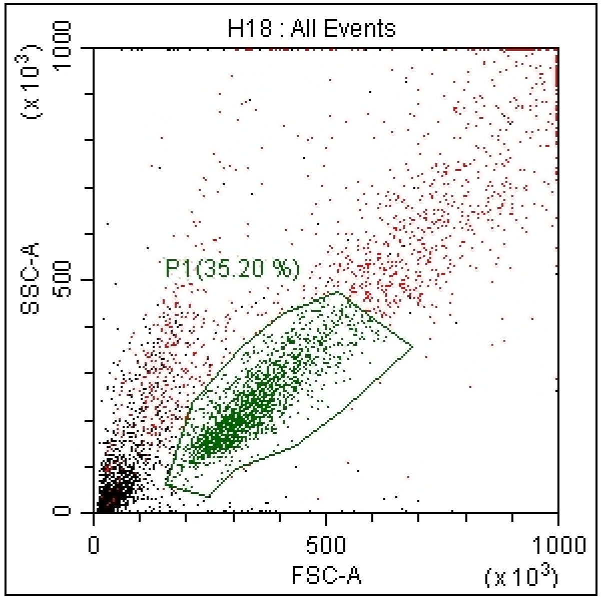

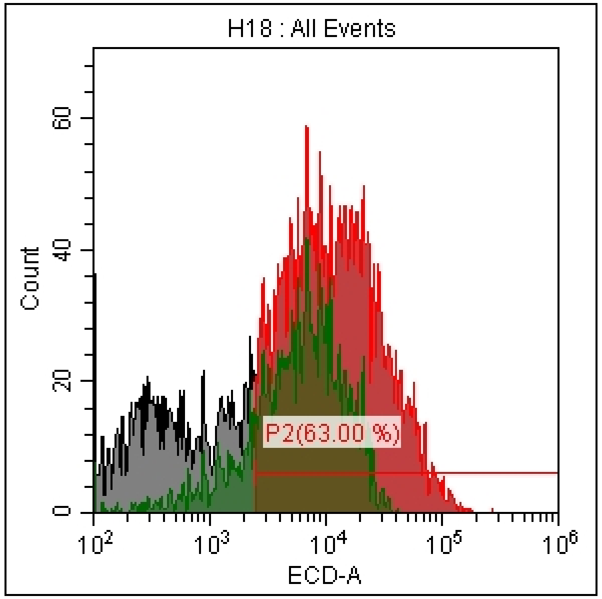

Experiment Name: KZ.20190422

Tube Name: H18

Sample ID:

Volume(μL): 145.9

Population	Mean FITC-A	Events	% Parent	Events/μL(V)	Median FITC-A	rCV FITC-A	...
All Events	43747.4	5000	100.00 %	34.27	22362.6	121.31 %	...
P2	65537.5	3150	63.00 %	21.59	38367.5	84.63 %	...
P1	23266.0	1760	35.20 %	12.06	21006.4	50.78 %	...
