# Supplementary material for: An anionic human protein mediates cationic liposome delivery of genome editing proteins into mammalian cells
Source: Nat Commun. 2019 Jul 2;10:2905. doi: 10.1038/s41467-019-10828-3 (PMC6606574; doi:10.1038/s41467-019-10828-3)
Supplement: Supplementary file 3 — Source data [file 41467_2019_10828_MOESM3_ESM.zip › Supplementary Figures 5 and 6/H2.pdf]

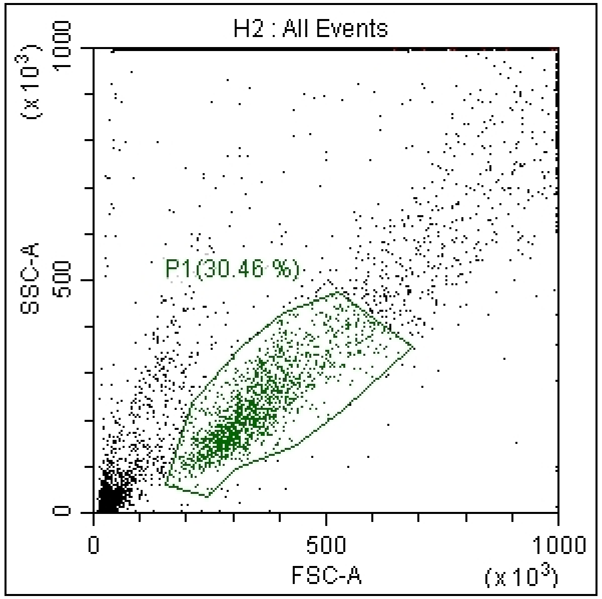

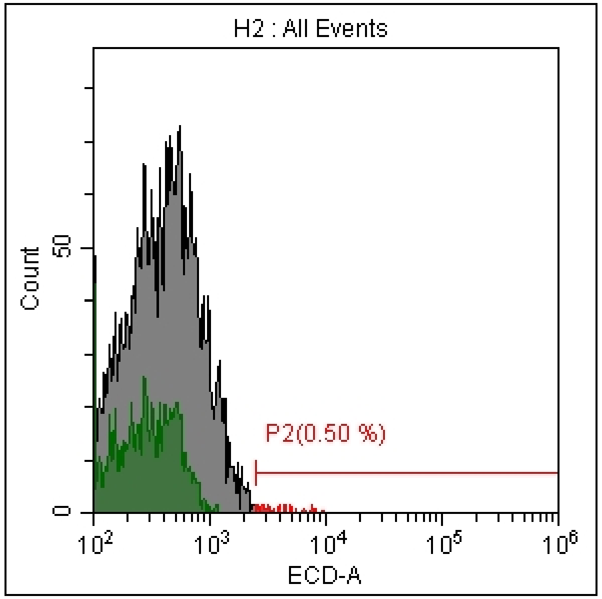

Experiment Name: KZ.20190422

Tube Name: H2

Sample ID:

Volume(μL): 100.8

Population	Mean FITC-A	Events	% Parent	Events/μL(V)	Median FITC-A	rCV FITC-A	...
All Events	30397.8	5000	100.00 %	49.63	15235.1	132.11 %	...
P2	104825.7	25	0.50 %	0.25	42848.3	104.81 %	...
P1	23392.8	1523	30.46 %	15.12	21046.6	52.42 %	...
