# Supplementary material for: An anionic human protein mediates cationic liposome delivery of genome editing proteins into mammalian cells
Source: Nat Commun. 2019 Jul 2;10:2905. doi: 10.1038/s41467-019-10828-3 (PMC6606574; doi:10.1038/s41467-019-10828-3)
Supplement: Supplementary file 3 — Source data [file 41467_2019_10828_MOESM3_ESM.zip › Supplementary Figures 5 and 6/H3.pdf]

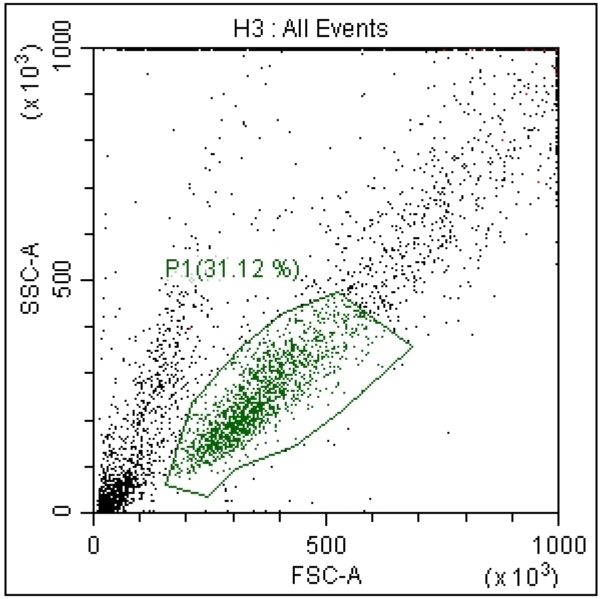

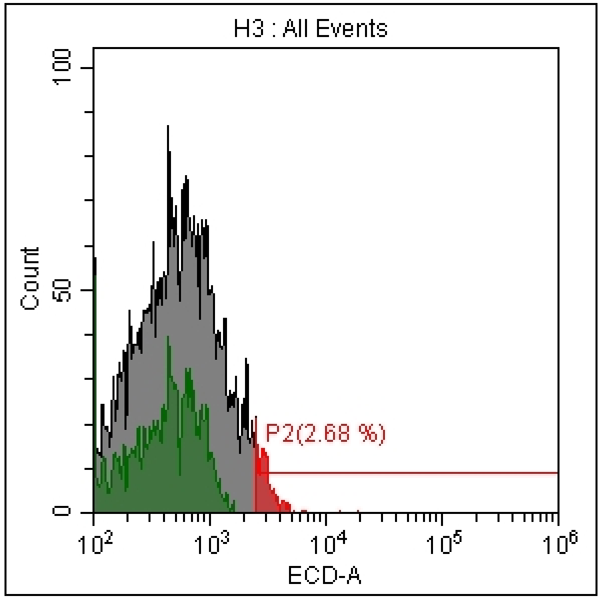

Experiment Name: KZ.20190422

Tube Name: H3

Sample ID:

Volume(µL): 91.5

Population	Mean FITC-A	Events	% Parent	Events/µL(V)	Median FITC-A	rCV FITC-A	...
All Events	39511.0	5000	100.00 %	54.66	20007.5	125.39 %	...
P2	234173.1	134	2.68 %	1.46	224947.0	38.24 %	...
P1	24083.3	1556	31.12 %	17.01	21707.7	49.50 %	...
