# Supplementary material for: An anionic human protein mediates cationic liposome delivery of genome editing proteins into mammalian cells
Source: Nat Commun. 2019 Jul 2;10:2905. doi: 10.1038/s41467-019-10828-3 (PMC6606574; doi:10.1038/s41467-019-10828-3)
Supplement: Supplementary file 3 — Source data [file 41467_2019_10828_MOESM3_ESM.zip › Supplementary Figures 5 and 6/H4.pdf]

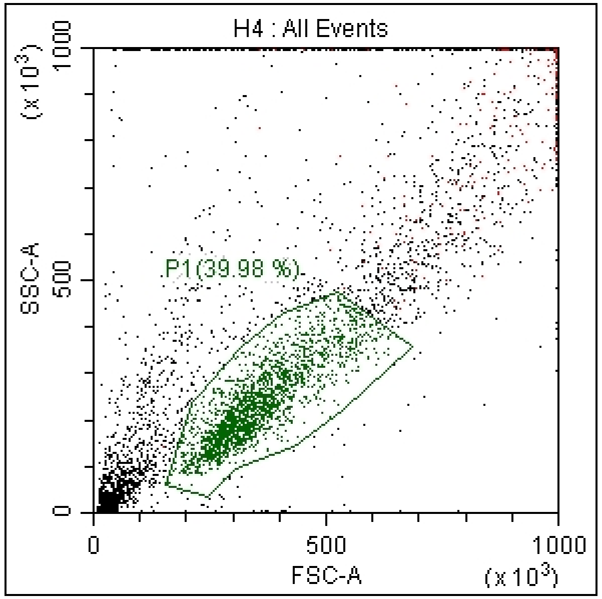

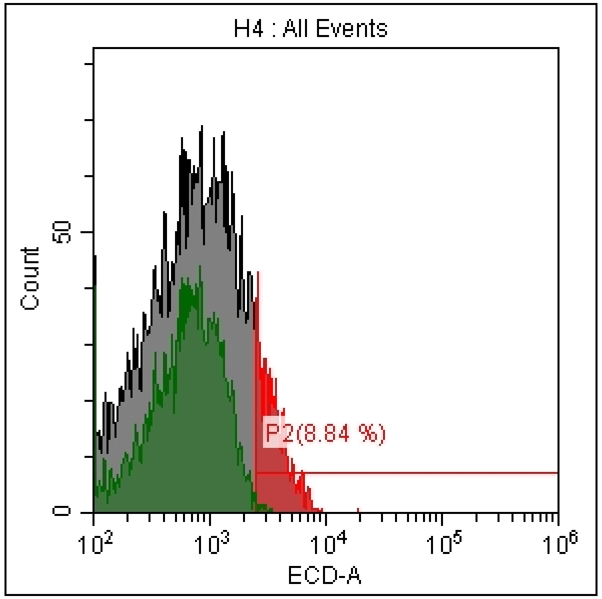

Experiment Name: KZ.20190422

Tube Name: H4

Sample ID:

Volume(μL): 101.5

Population	Mean FITC-A	Events	% Parent	Events/μL(V)	Median FITC-A	rCV FITC-A	...
All Events	41406.3	5000	100.00 %	49.24	23519.6	119.95 %	...
P2	151349.7	442	8.84 %	4.35	138403.0	48.79 %	...
P1	24987.0	1999	39.98 %	19.69	22071.3	50.43 %	...
