# Supplementary material for: An anionic human protein mediates cationic liposome delivery of genome editing proteins into mammalian cells
Source: Nat Commun. 2019 Jul 2;10:2905. doi: 10.1038/s41467-019-10828-3 (PMC6606574; doi:10.1038/s41467-019-10828-3)
Supplement: Supplementary file 3 — Source data [file 41467_2019_10828_MOESM3_ESM.zip › Supplementary Figures 5 and 6/H5.pdf]

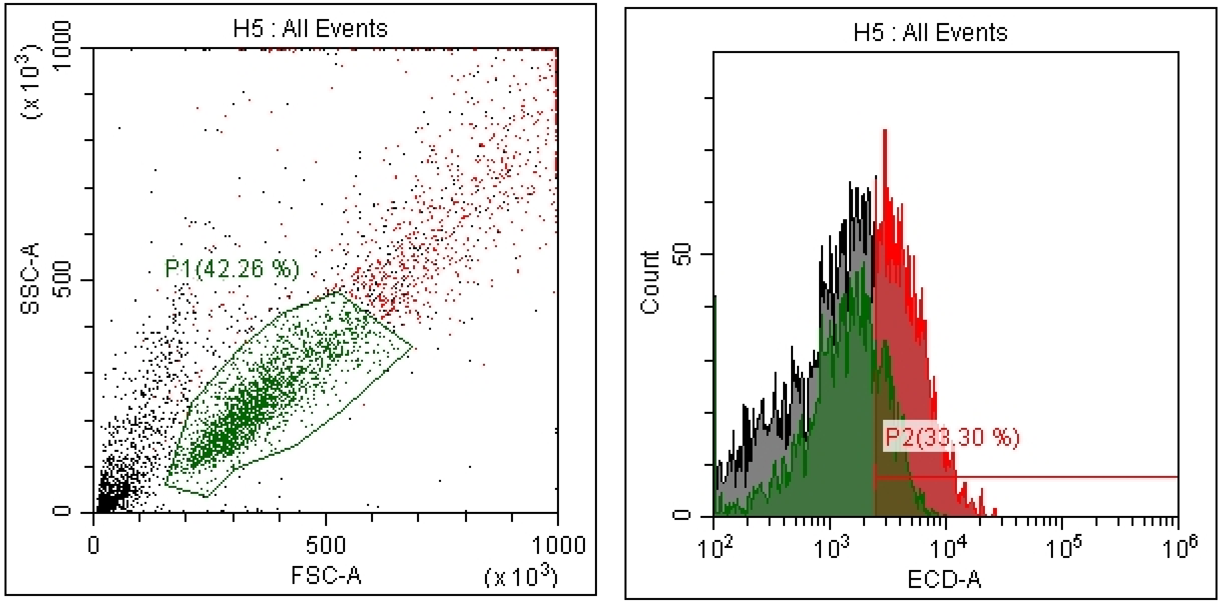

Experiment Name: KZ.20190422

Tube Name: H5

Sample ID:

Volume(μL): 100.5

Population	Median FITC-A	rCV FITC-A	rSD FITC-A	CV FITC-A	SD FITC-A	% Total	P...
All Events	26261.6	111.51 %	29283.1	128.38 %	55853.0	100.0...	#...
P2	72149.0	71.04 %	51257.2	78.95 %	70267.8	33.30 %	Al...
P1	22430.1	52.80 %	11844.2	56.11 %	14264.5	42.26 %	Al...
