# Supplementary material for: An anionic human protein mediates cationic liposome delivery of genome editing proteins into mammalian cells
Source: Nat Commun. 2019 Jul 2;10:2905. doi: 10.1038/s41467-019-10828-3 (PMC6606574; doi:10.1038/s41467-019-10828-3)
Supplement: Supplementary file 3 — Source data [file 41467_2019_10828_MOESM3_ESM.zip › Supplementary Figures 5 and 6/H6.pdf]

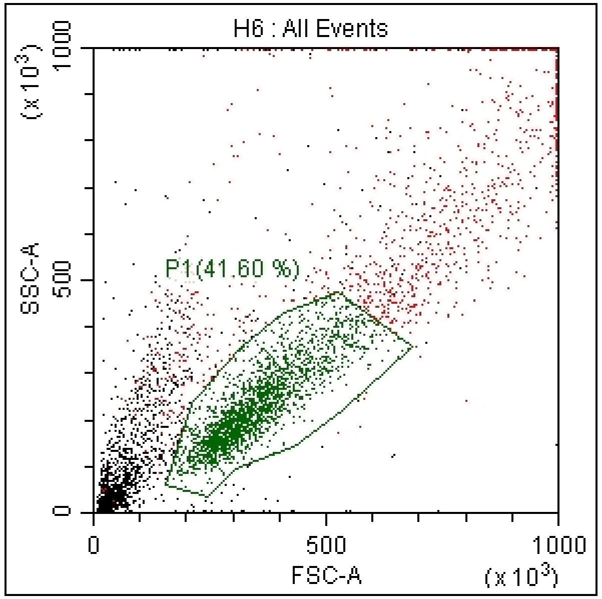

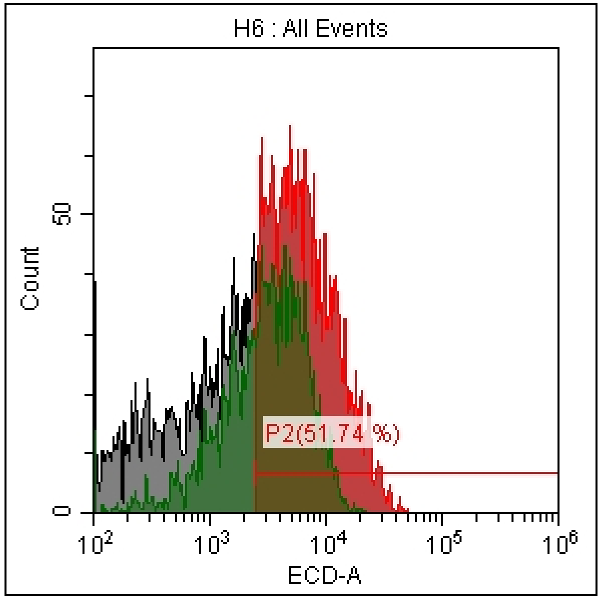

Experiment Name: KZ.20190422

Tube Name: H6

Sample ID:

Volume(μL): 104.0

Population	Mean FITC-A	Events	% Parent	Events/μL(V)	Median FITC-A	rCV FITC-A	...
All Events	36885.8	5000	100.00 %	48.09	22241.9	116.41 %	...
P2	61516.1	2587	51.74 %	24.88	44298.0	79.39 %	...
P1	24537.9	2080	41.60 %	20.01	21411.1	50.48 %	...
