# Supplementary material for: An anionic human protein mediates cationic liposome delivery of genome editing proteins into mammalian cells
Source: Nat Commun. 2019 Jul 2;10:2905. doi: 10.1038/s41467-019-10828-3 (PMC6606574; doi:10.1038/s41467-019-10828-3)
Supplement: Supplementary file 3 — Source data [file 41467_2019_10828_MOESM3_ESM.zip › Supplementary Figures 5 and 6/H7.pdf]

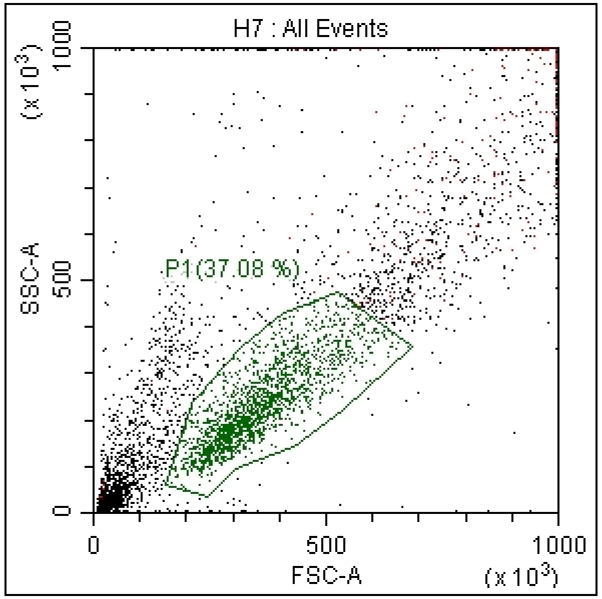

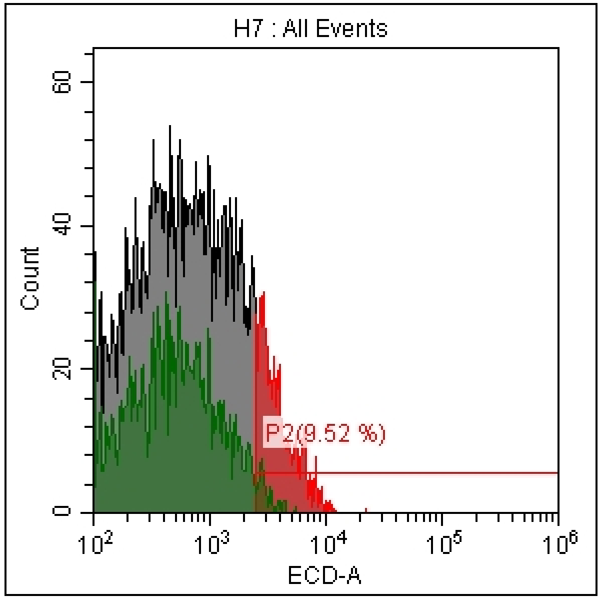

Experiment Name: KZ.20190422

Tube Name: H7

Sample ID:

Volume(μL): 119.5

Population	Mean FITC-A	Events	% Parent	Events/μL(V)	Median FITC-A	rCV FITC-A	...
All Events	93834.1	5000	100.00 %	41.84	38649.5	135.71 %	...
P2	417417.9	476	9.52 %	3.98	391895.7	43.19 %	...
P1	57603.0	1854	37.08 %	15.52	35817.5	77.28 %	...
