# Supplementary material for: An anionic human protein mediates cationic liposome delivery of genome editing proteins into mammalian cells
Source: Nat Commun. 2019 Jul 2;10:2905. doi: 10.1038/s41467-019-10828-3 (PMC6606574; doi:10.1038/s41467-019-10828-3)
Supplement: Supplementary file 3 — Source data [file 41467_2019_10828_MOESM3_ESM.zip › Supplementary Figures 5 and 6/H8.pdf]

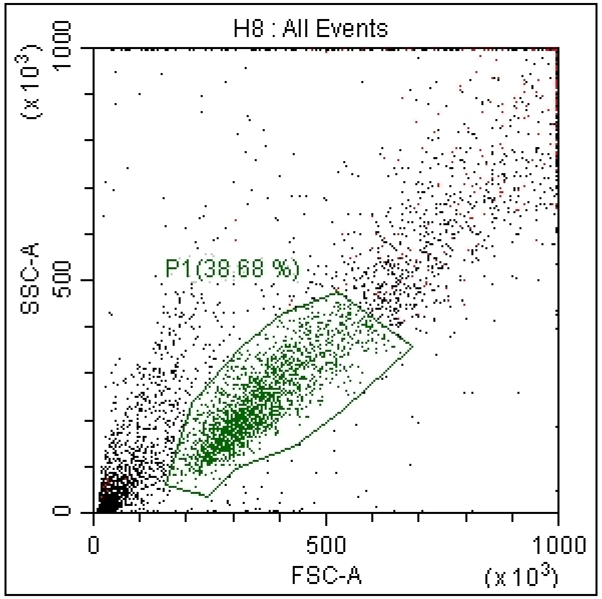

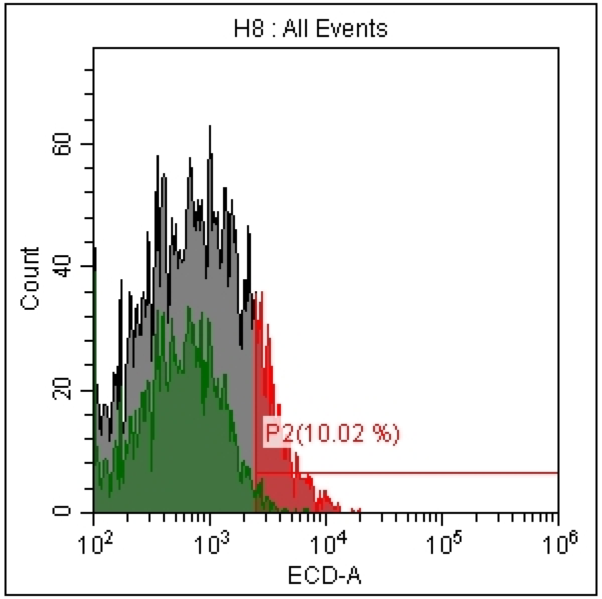

Experiment Name: KZ.20190422

Tube Name: H8

Sample ID:

Volume(µL): 84.9

Population	Mean FITC-A	Events	% Parent	Events/µL(V)	Median FITC-A	rCV FITC-A	...
All Events	88306.9	5000	100.00 %	58.86	39805.8	130.01 %	...
P2	379771.3	501	10.02 %	5.90	329908.7	40.33 %	...
P1	51040.2	1934	38.68 %	22.77	36192.4	71.54 %	...
