# Supplementary material for: An anionic human protein mediates cationic liposome delivery of genome editing proteins into mammalian cells
Source: Nat Commun. 2019 Jul 2;10:2905. doi: 10.1038/s41467-019-10828-3 (PMC6606574; doi:10.1038/s41467-019-10828-3)
Supplement: Supplementary file 3 — Source data [file 41467_2019_10828_MOESM3_ESM.zip › Supplementary Figures 5 and 6/H9.pdf]

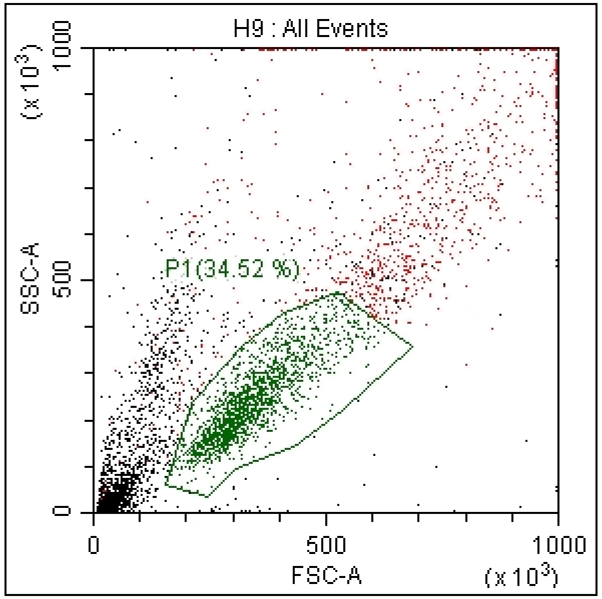

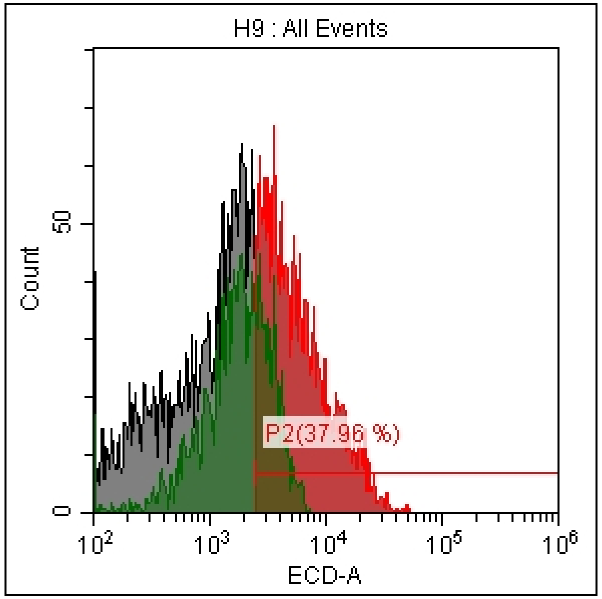

Experiment Name: KZ.20190422

Tube Name: H9

Sample ID:

Volume(μL): 102.3

Population	Mean FITC-A	Events	% Parent	Events/μL(V)	Median FITC-A	rCV FITC-A	...
All Events	54893.6	5000	100.00 %	48.88	25915.9	126.63 %	...
P2	117262.2	1898	37.96 %	18.55	80945.7	79.17 %	...
P1	28309.0	1726	34.52 %	16.87	24144.1	51.16 %	...
