# Supplementary figures and images for: An anionic human protein mediates cationic liposome delivery of genome editing proteins into mammalian cells
Source: Nat Commun. 2019 Jul 2;10:2905. doi: 10.1038/s41467-019-10828-3 (PMC6606574; doi:10.1038/s41467-019-10828-3)

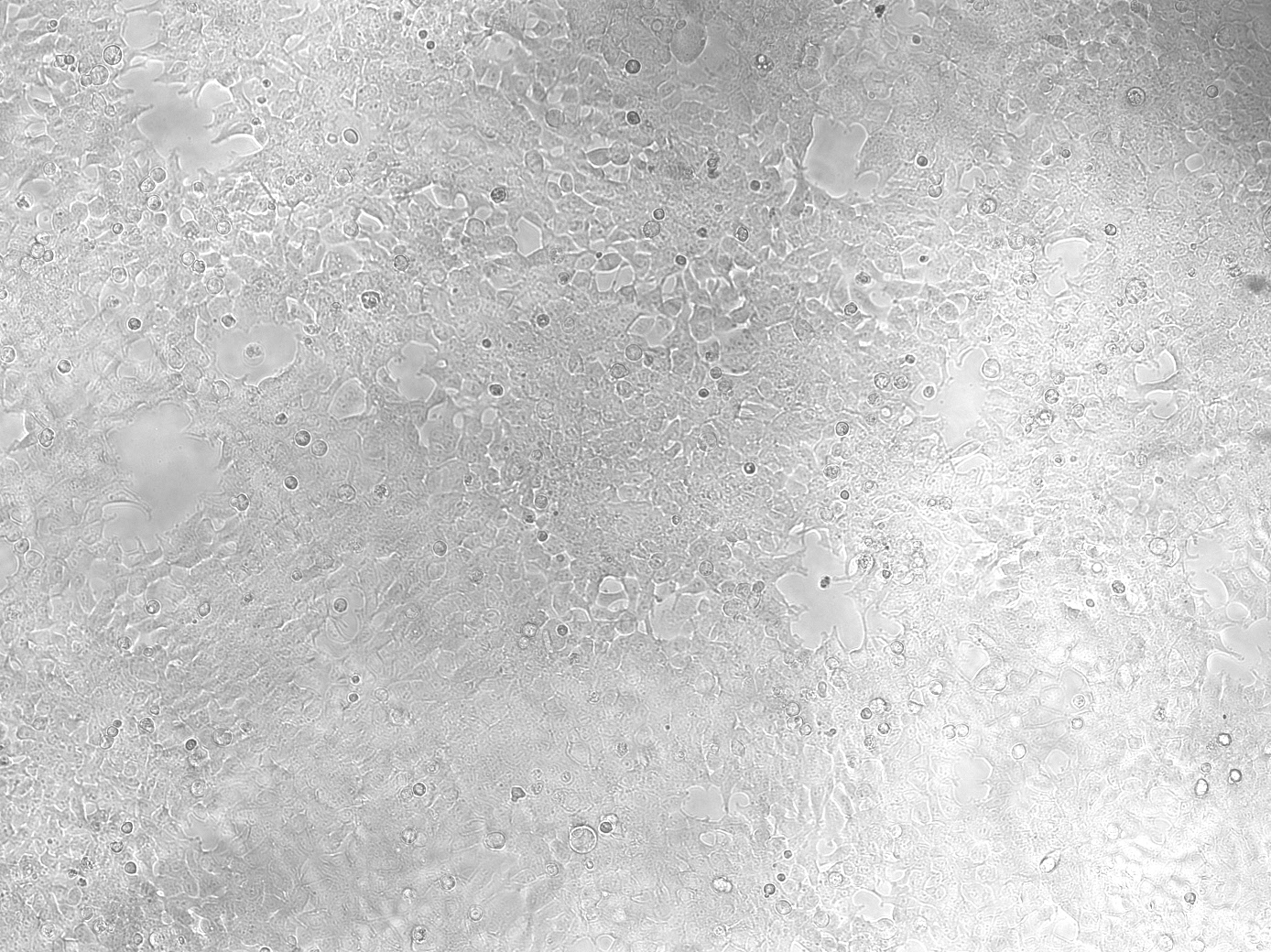

Supplement: Supplementary file 3 — Source data [file 41467_2019_10828_MOESM3_ESM.zip › Supplementary Figure 3/cre4-br.jpg]

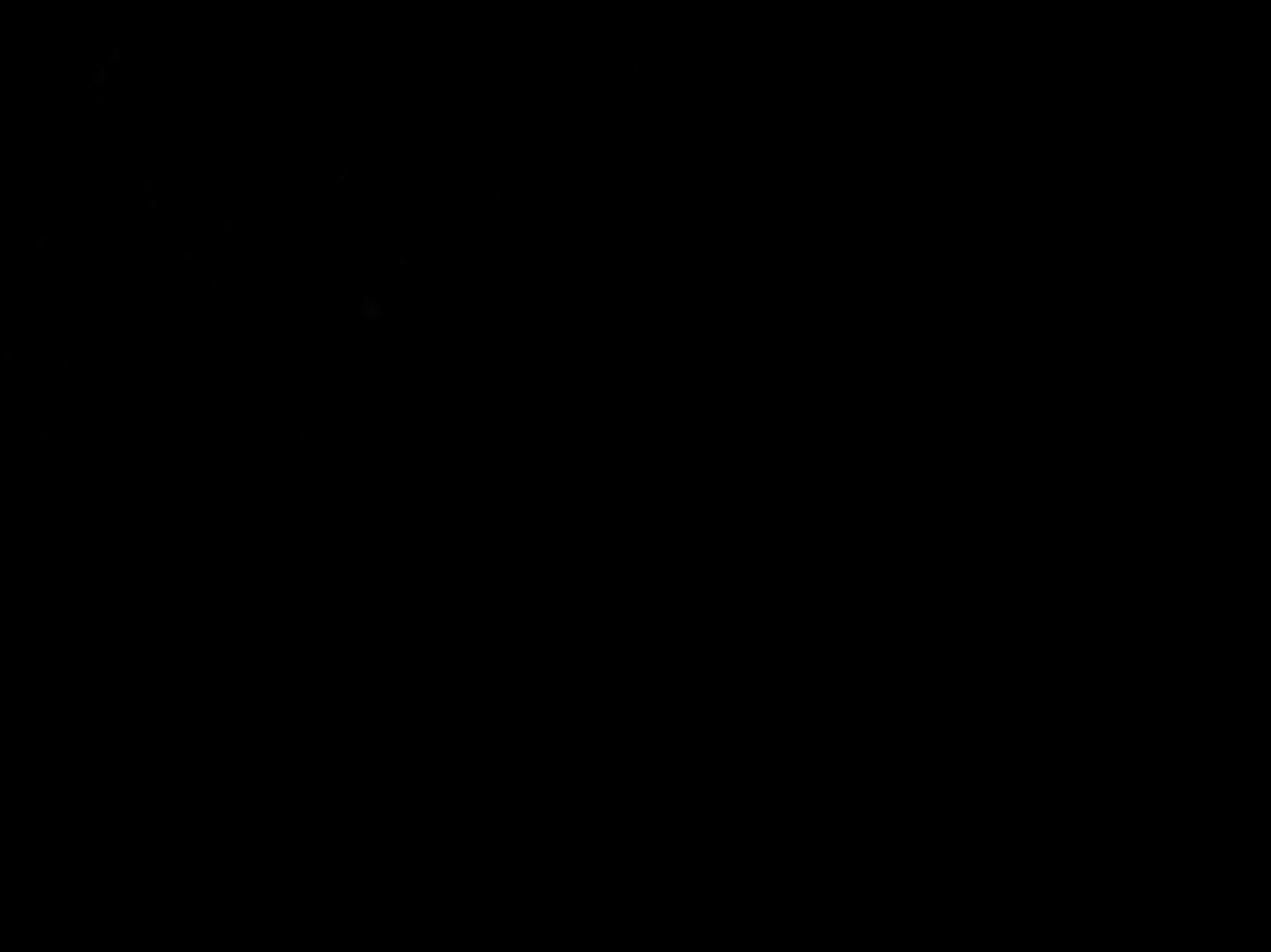

Supplement: Supplementary file 3 — Source data [file 41467_2019_10828_MOESM3_ESM.zip › Supplementary Figure 3/cre4-r.jpg]

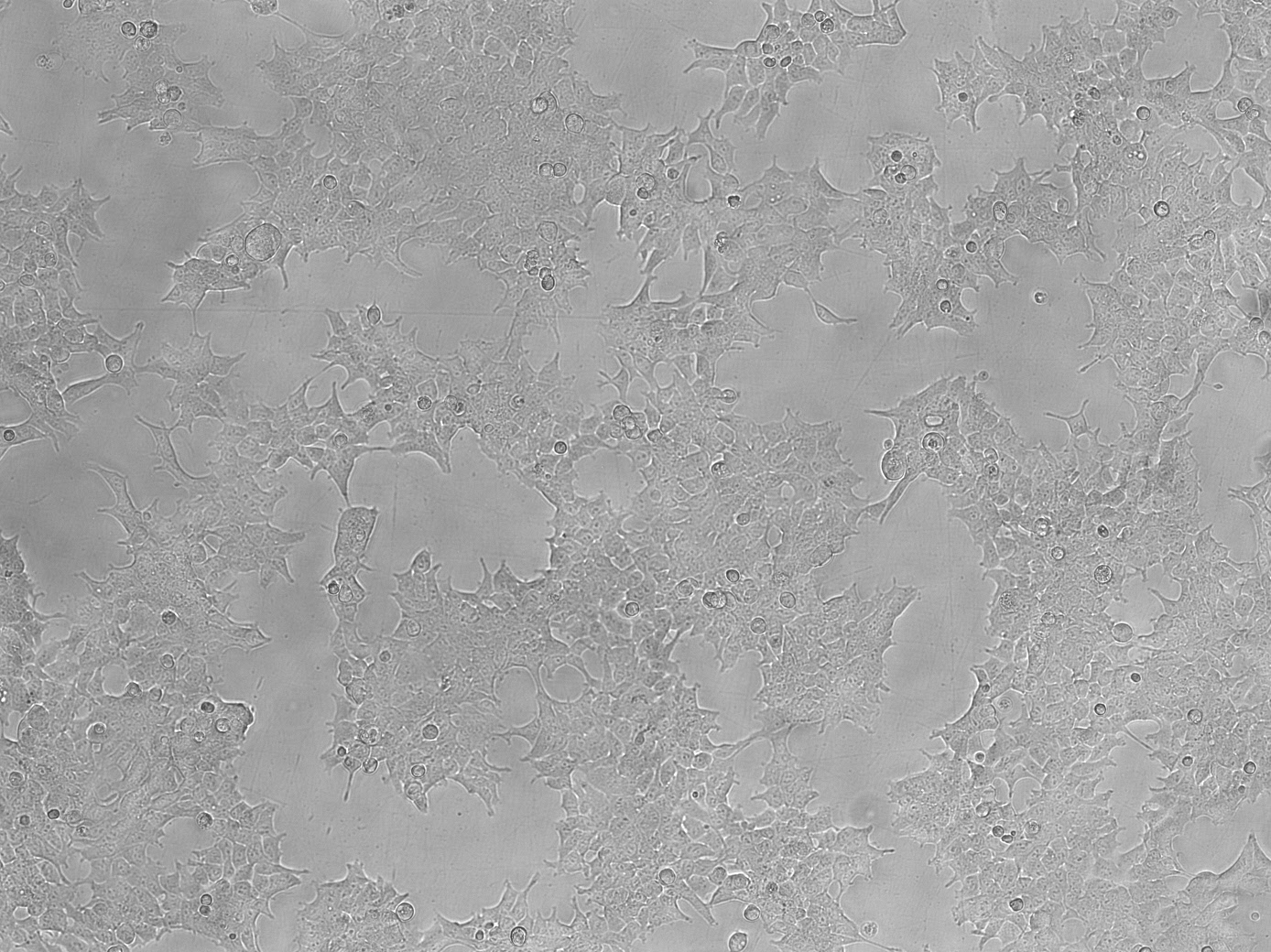

Supplement: Supplementary file 3 — Source data [file 41467_2019_10828_MOESM3_ESM.zip › Supplementary Figure 3/cre7-br.jpg]

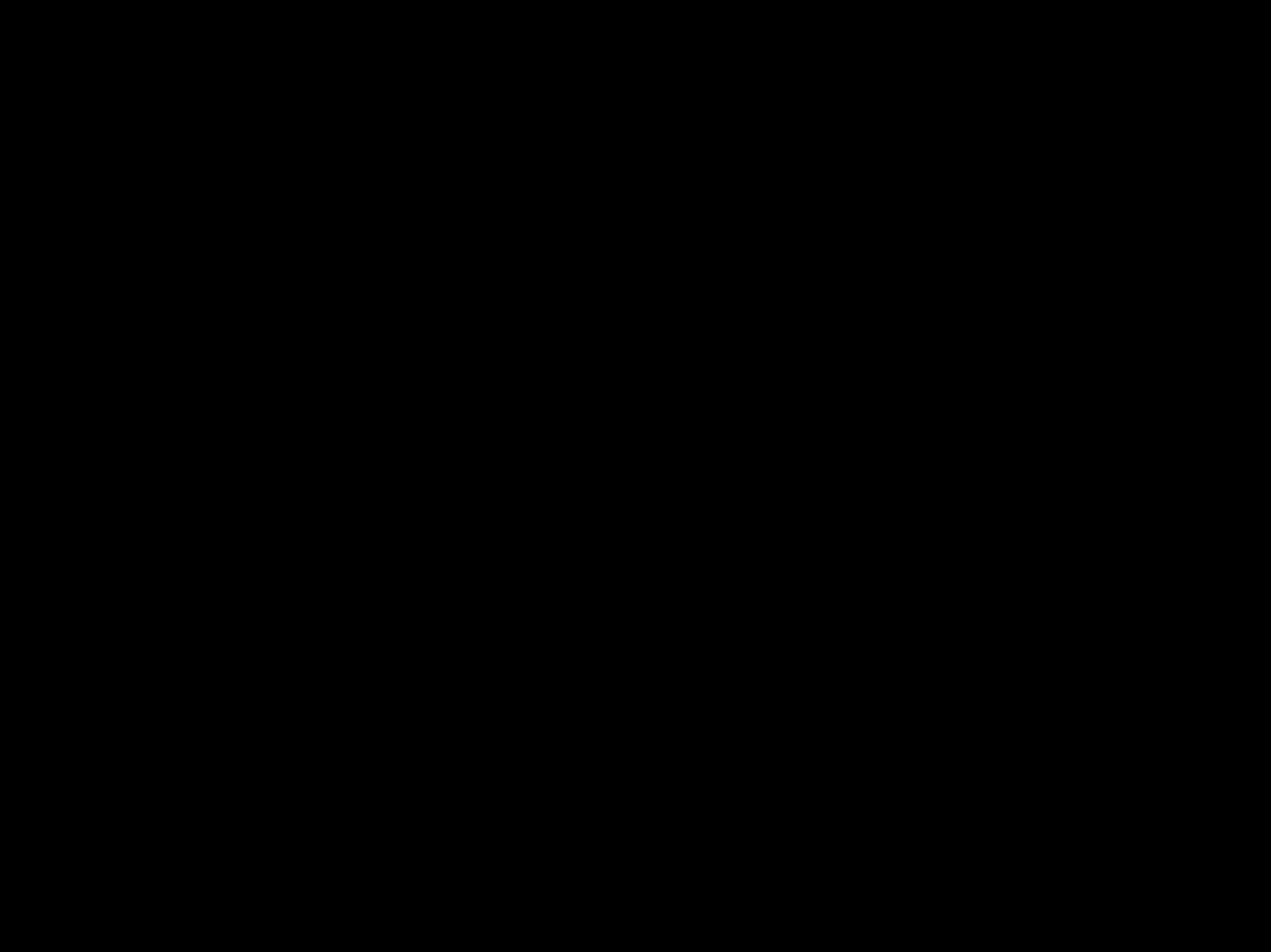

Supplement: Supplementary file 3 — Source data [file 41467_2019_10828_MOESM3_ESM.zip › Supplementary Figure 3/cre7-r.jpg]

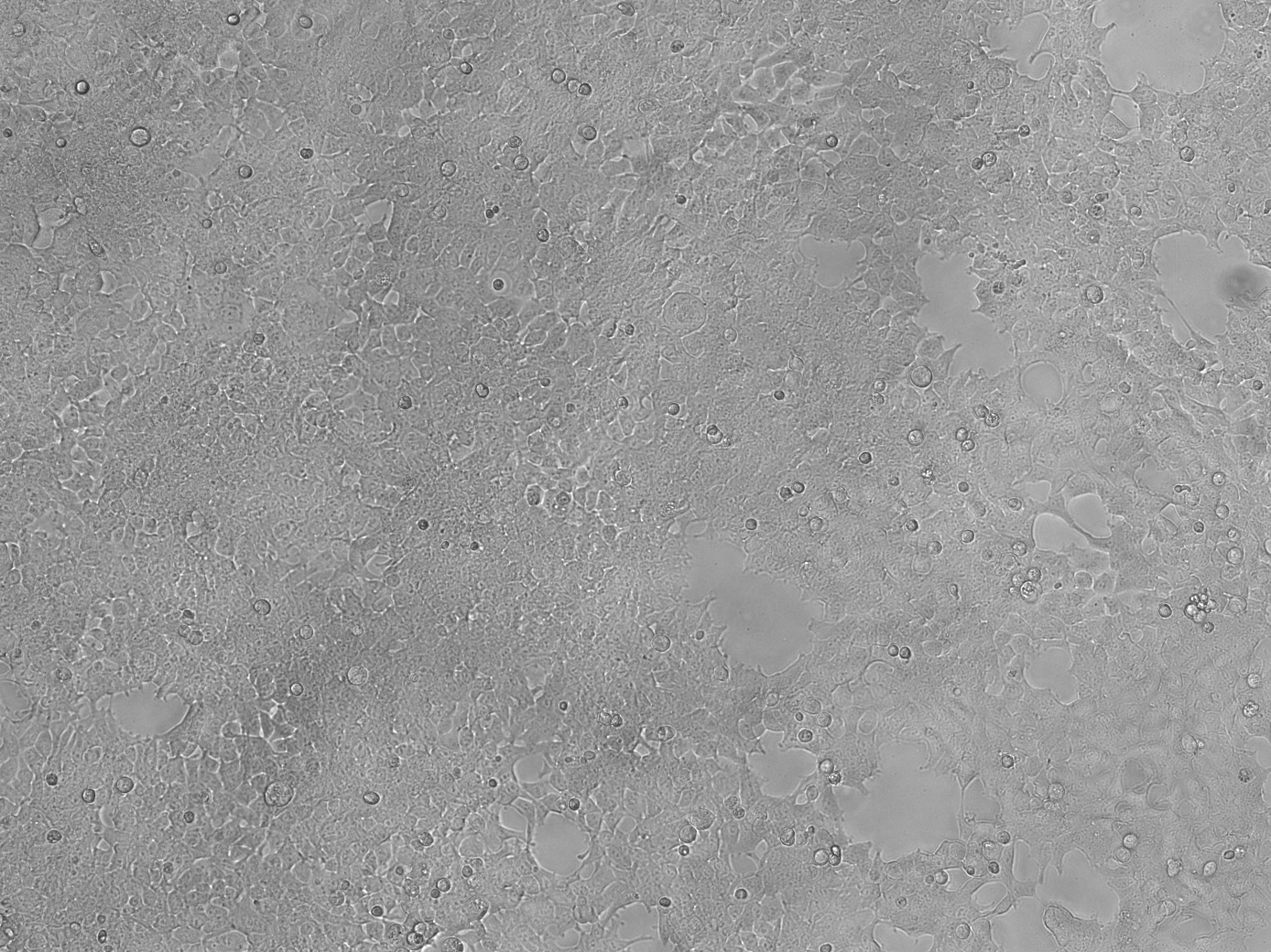

Supplement: Supplementary file 3 — Source data [file 41467_2019_10828_MOESM3_ESM.zip › Supplementary Figure 3/gfp4-br.jpg]

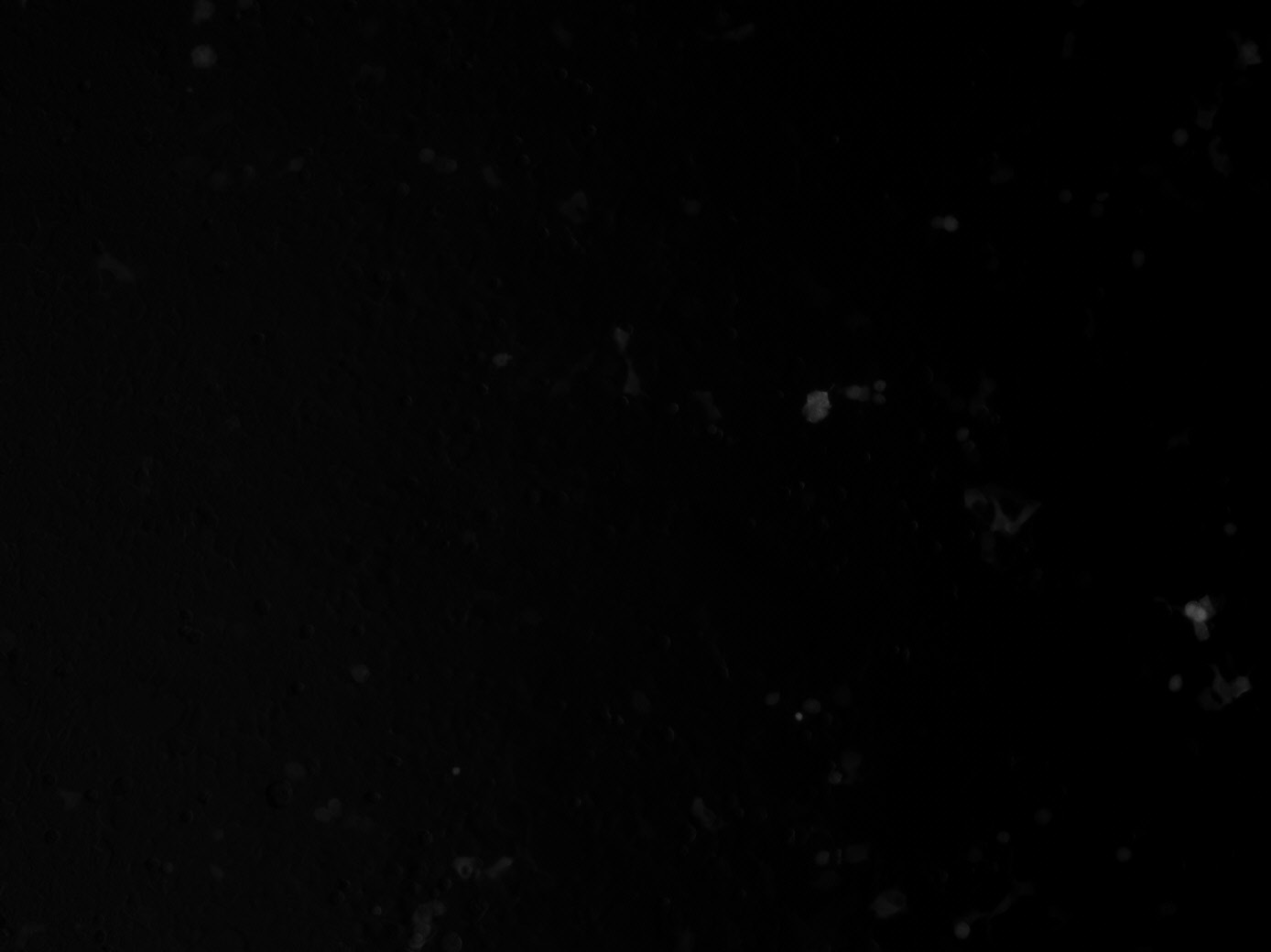

Supplement: Supplementary file 3 — Source data [file 41467_2019_10828_MOESM3_ESM.zip › Supplementary Figure 3/gfp4-r.jpg]

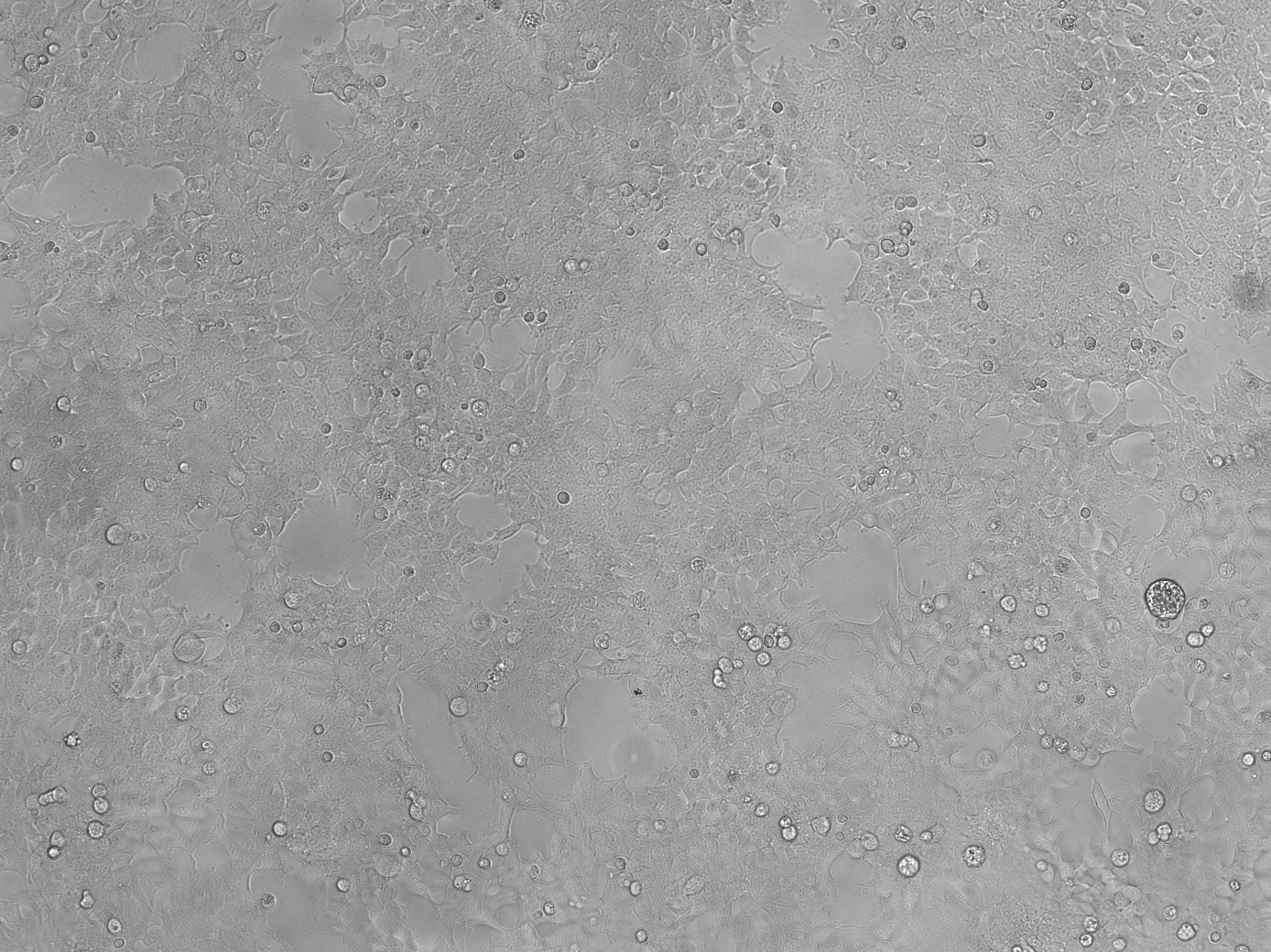

Supplement: Supplementary file 3 — Source data [file 41467_2019_10828_MOESM3_ESM.zip › Supplementary Figure 3/gfp7-br.jpg]

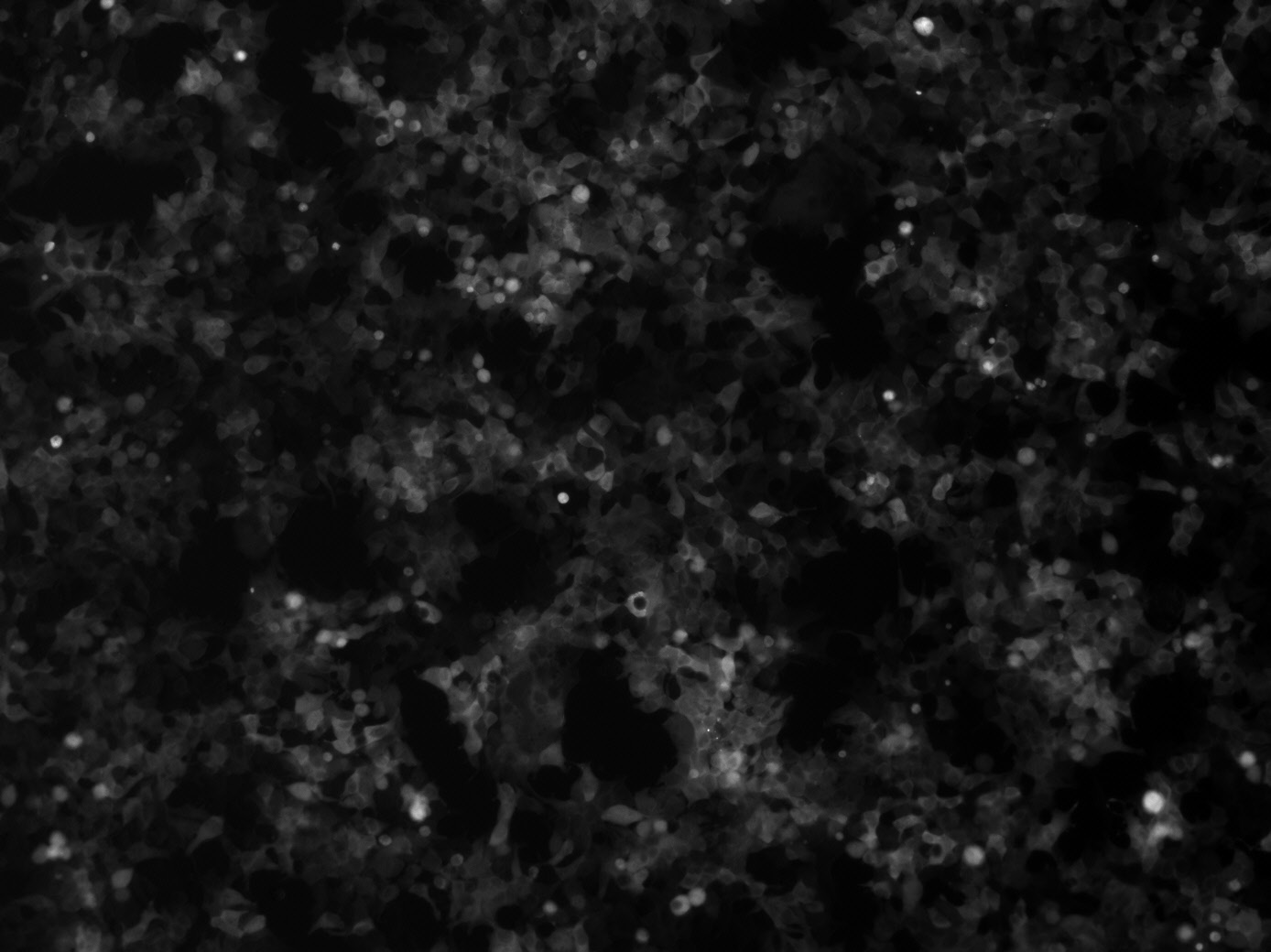

Supplement: Supplementary file 3 — Source data [file 41467_2019_10828_MOESM3_ESM.zip › Supplementary Figure 3/gfp7-r.jpg]

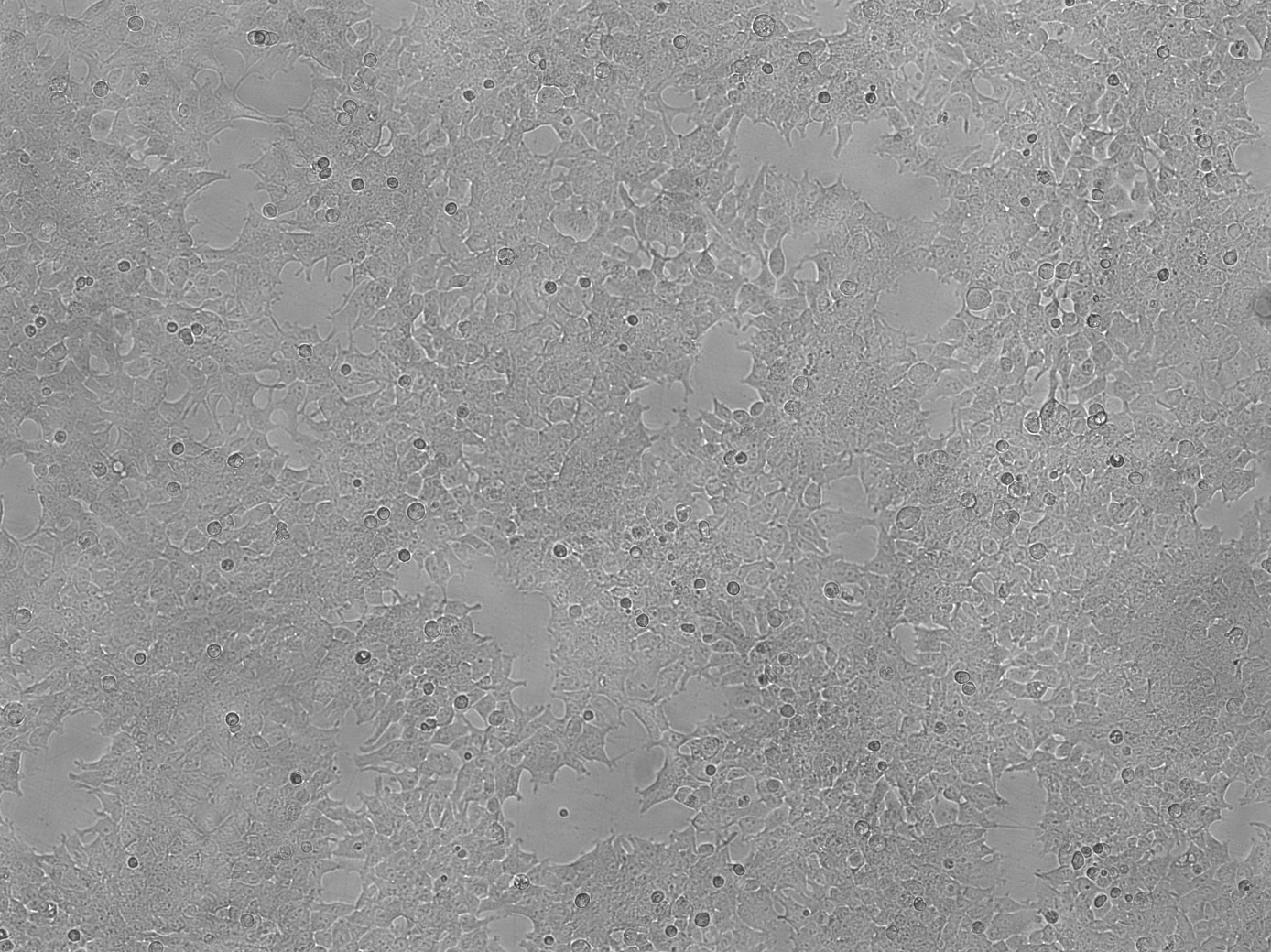

Supplement: Supplementary file 3 — Source data [file 41467_2019_10828_MOESM3_ESM.zip › Supplementary Figure 3/pt4-br.jpg]

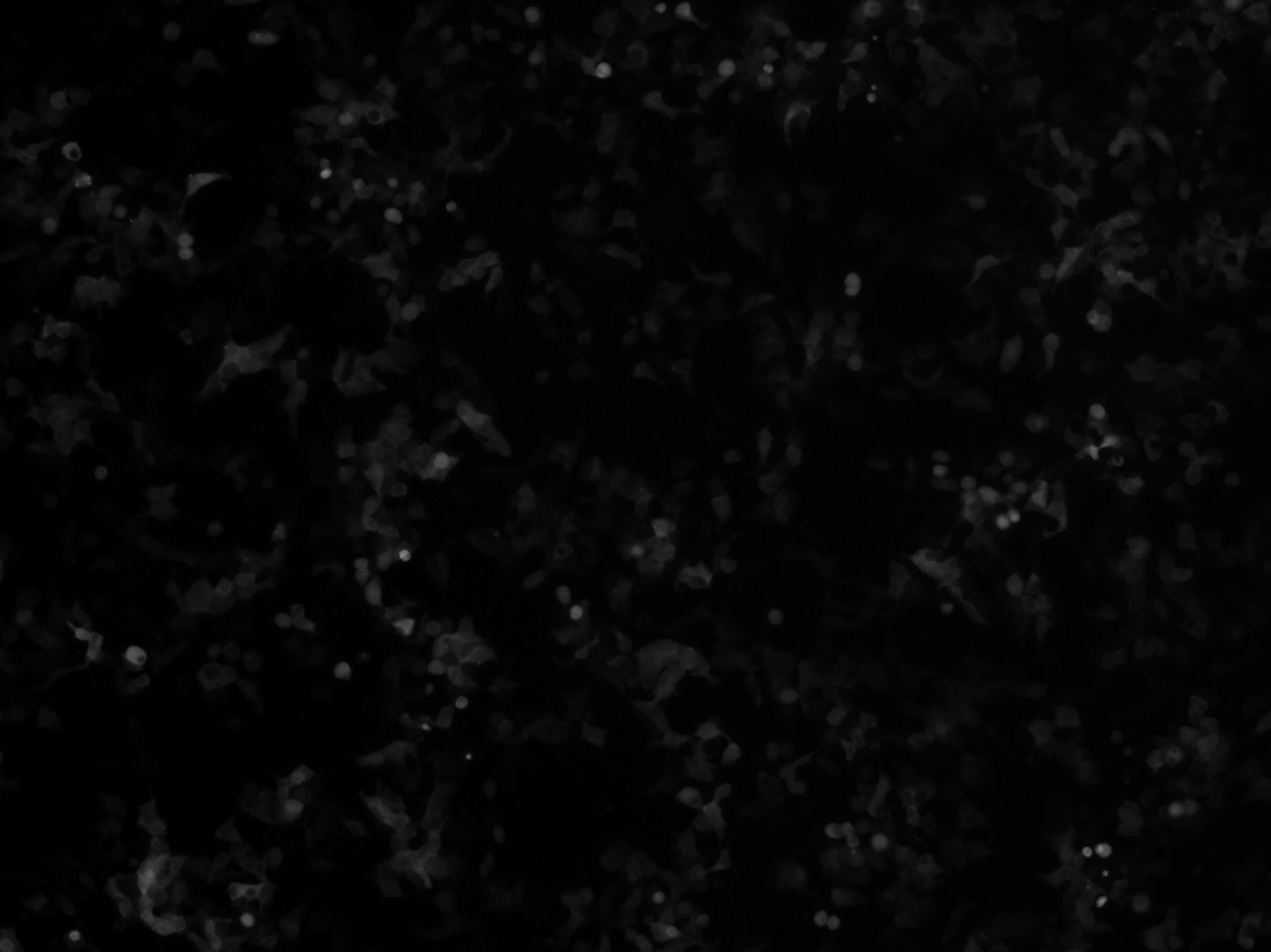

Supplement: Supplementary file 3 — Source data [file 41467_2019_10828_MOESM3_ESM.zip › Supplementary Figure 3/pt4-r.jpg]

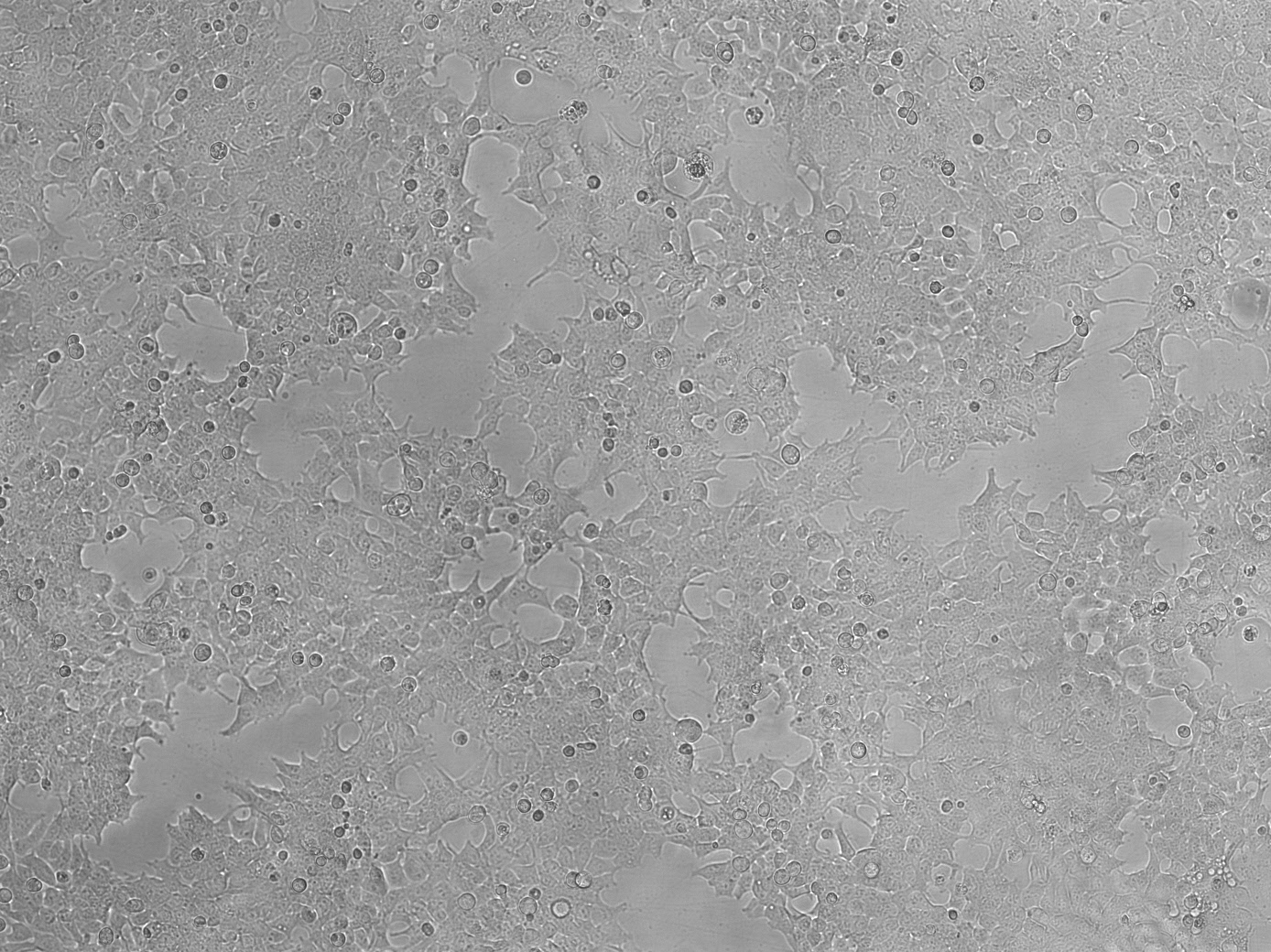

Supplement: Supplementary file 3 — Source data [file 41467_2019_10828_MOESM3_ESM.zip › Supplementary Figure 3/pt7-br.jpg]

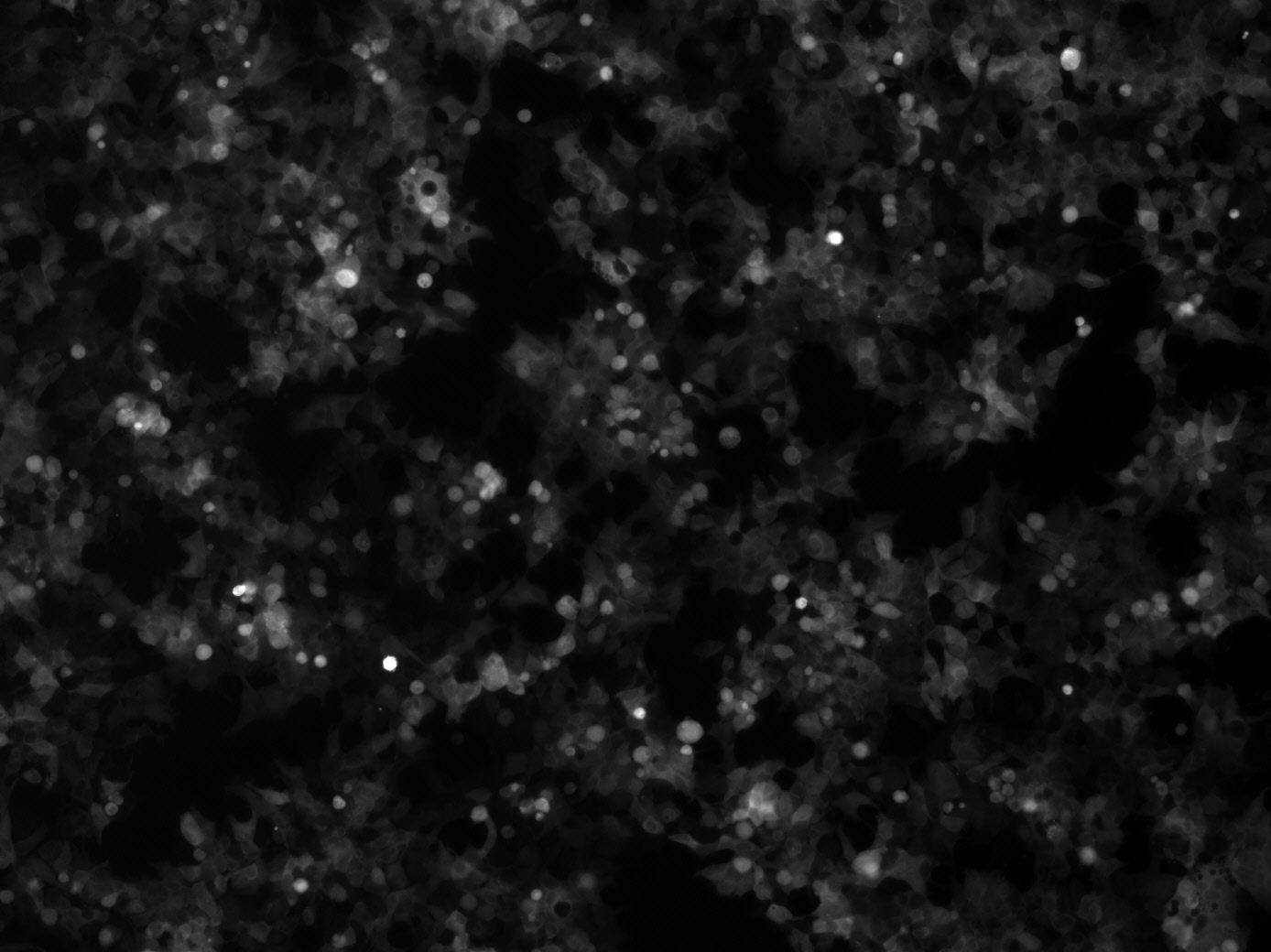

Supplement: Supplementary file 3 — Source data [file 41467_2019_10828_MOESM3_ESM.zip › Supplementary Figure 3/pt7-r.jpg]
